# A Straightforward Approach Towards Phosphadecalones by Microwave-Assisted Diels–Alder Reaction

**DOI:** 10.3390/molecules30112338

**Published:** 2025-05-27

**Authors:** Elżbieta Łastawiecka, Anna E. Kozioł, K. Michał Pietrusiewicz

**Affiliations:** Faculty of Chemistry, Maria Curie-Skłodowska University, 20-031 Lublin, Poland; kazimierz.pietrusiewicz@mail.umcs.pl

**Keywords:** phosphadecalone, P-stereogenic, phosphinanes, [4+2] cycloaddition, 1-phenylphosphin-2-en-4-one 1-oxide, optically active phosphines

## Abstract

A stereoselective and scalable strategy for the synthesis of phosphorus-containing bicyclic and tricyclic compounds from 1-phenylphosphin-2-en-4-one 1-oxide is presented. This activated dienophile, available in both racemic and enantiopure forms, undergoes smooth [4+2] cycloadditions with acyclic and cyclic dienes, affording products with excellent yields and controlled stereochemistry. Notably, the *cis*/*trans*-fusion of the cycloadducts (phosphadecalones and phosphahexahydrochrysene) can be selectively controlled by fine-tuning the conditions of microwave-assisted cycloaddition reaction. The influence of temperature, time, and steric effects on *cis*/*trans* and *endo*/*exo* selectivity was examined in detail. The molecular structure, including the absolute configuration, of eight products has been determined by X-ray crystallography. These analyses further established the *endo*-selective nature of the cycloaddition, favoring the P=O face of the dienophile. Post-cycloaddition transformations of selected *P*-stereogenic phosphadecalone, such as isomerization, reduction and deoxygenation, demonstrate the synthetic versatility of the resulting products.

## 1. Introduction

The bicyclo[4.4.0]decanone (decalone) motif is the scaffold in a number of synthetic and natural compounds showing a variety of biological activities. These include salvinorin B [1], myrrhanone B [2], prednisolone [3], dehydrocholic acid [4], dexamethasone [5] and cortisone [6]. Although N- and O-heterocyclic decalone analogs are widely represented and have been extensively investigated [7,8,9], their phosphorus-containing counterparts (phosphadecalones) remain relatively underexplored, despite their potential in both medicinal and coordination chemistry.

**Figure 1:**
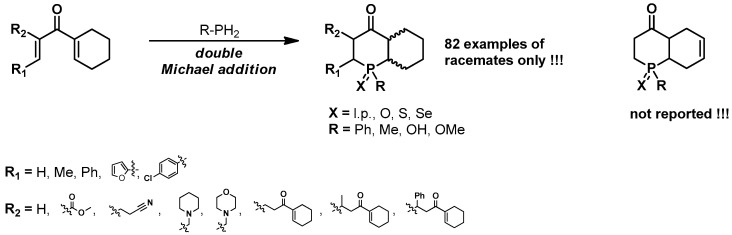
Examples of syntheses of phosphadecalones by double Michael addition [10,11,12,13].

This limited representation can be attributed primarily to synthetic challenges. One of the most popular synthetic strategies used to obtain phosphadecalone core is to employ a double Michael addition of primary phosphines RPH_2_ to vinyl cyclohexenyl ketone derivatives (Figure 1) [10,11,12]. Another established route involves the conjugate addition of methyl 2-methoxycarbonylethylphosphinate to methyl cyclohex-1-ene 1-carboxylate followed by Claisen-type cyclization [13]. Unfortunately, the scope of these methodologies has so far been limited to the synthesis of fully saturated, racemic derivatives, with access typically limited to a single *cis*-fused stereoisomer.

At the same time, six-membered phosphorus heterocycles (phosphinanes) have attracted considerable attention due to their expanding utility across multiple areas of chemistry [14,15]. These systems effectively integrate the electronic and coordinative properties of phosphorus with the structural rigidity of heterocycles, enabling their application as chiral ligands in homogeneous catalysis [16,17,18,19], pharmacophore in drug discovery [20,21,22,23,24], and potential as versatile synthons for the construction of complex polycyclic scaffolds [25,26,27,28,29,30,31,32,33].

Despite the increasing relevance of phosphinanes in synthetic chemistry, their application as dienophiles in [4+2] cycloadditions remains largely unexplored. Existing literature mainly describes their use as dienes [34,35]. To date, only one example has been reported in which a methyl-4-chloro-1,2-dihydrophosphinine oxides acted as a dienophile in a self-dimerization process [36]. No intermolecular Diels–Alder reactions involving phosphinanes as dienophiles with either acyclic or cyclic dienes have been reported.

**Figure 2:**
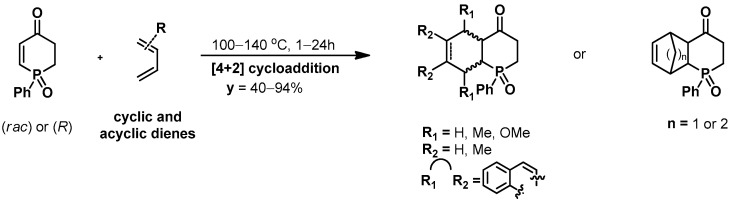
Proposed strategies for the synthesis of new racemic and enantiopure phosphadecalones.

In this context, the development of modular and stereoselective strategies for the synthesis of bi- and polycyclic phosphorus-containing compounds is of significant interest. Herein, we report a versatile strategy based on [4+2] cycloaddition reactions of 1-phenylphosphin-2-en-4-one 1-oxide with a variety of dienes, enabling access to both racemic and enantiomerically pure fused phosphorus heterocycles (Figure 2).

1-Phenylphosphin-2-en-4-one 1-oxide was selected as a synthetically accessible dienophile bearing a conjugated C=C–C=O unit, which is known to enhance reactivity in Diels–Alder reactions through electronic activation [37]. While the five-membered phosphorus-containing counterpart (1-phenylphosphol-2-ene 1-oxide) has previously been employed in [4+2] cycloadditions, it requires harsh conditions and exhibits limited reactivity, highlighting the need for improved scaffolds [38,39,40]. Notably, both enantiomers of 1-phenylphosphin-2-en-4-one 1-oxide are readily available in optically pure form through separating the formed diastereoisomeric molecular complexes with TADDOL, allowing for gram-scale quantities in technically simple conditions [41]. Consequently, the methodology developed herein can be readily extended to the synthesis of chiral bi- and polycyclic phosphorus compounds. The ability to access these frameworks in optically pure form is particularly relevant in the context of their potential applications in asymmetric catalysis and pharmaceutical development.

## 2. Results and Discussion

### 2.1. Synthesis of Bicyclic Phenylphosphin-2-en-4-one Derivatives

**Scheme 1:**
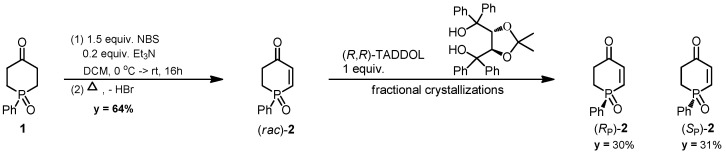
Synthesis and resolution of 1-phenylphosphin-2-en-4-one 1-oxide (**2**) using (*R*,*R*)-TADDOL [37,41].

The ready availability of racemic 1-phenylphosphin-2-en-4-one 1-oxide (*rac*-**2**) was a key starting point in our exploration of stereoselective phosphorus heterocycle synthesis via Diels–Alder cycloaddition. Starting from 1-phenylphosphine-4-one 1-oxide, (**1**) selecting α-bromination of the carbonyl group utilizing N-bromosuccinimide (NBS), followed by spontaneous elimination of HBr, provided *rac*-**2** in 64% yield (Scheme 1) [37]. The subsequent resolution of *rac*-**2** was achieved via formation of its complexes with (*R*,*R*)-TADDOL, affording both enantiomers in gram-scale quantities [41].

**Figure 3:**
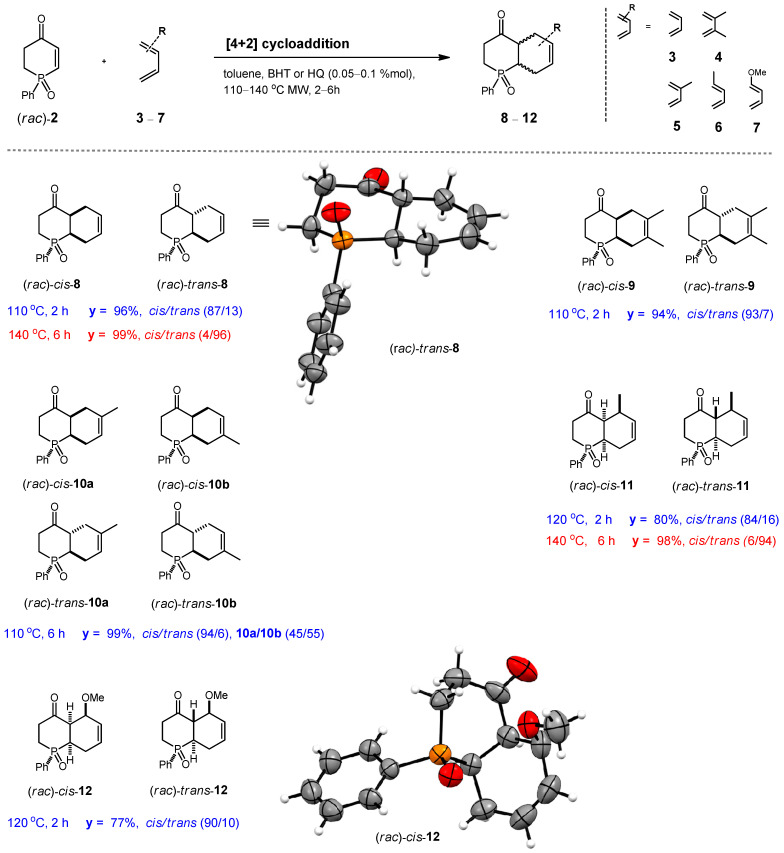
Cycloaddition reactions of a variety of acyclic dienes to (*rac*)-**2** as a dienophile and molecular structures of (*rac*)*-trans*-fused-**8** and (*rac*)*-cis*-fused-**12**. Yield (**y**) and stereoisomer ratios were determined by ^31^P NMR analysis. The conditions under which the reactions were carried out are given, *viz*. 110–140 °C, MW, 2–6 h.

In order to evaluate and optimize the cycloaddition reactivity of the studied dienophile *rac*-**2**, butadiene (**3**) was initially selected as the representative diene. The reaction proceeded efficiently under significantly milder conditions than those required for the related 1-phenylphosphol-2-ene system, which demands temperatures above 200 °C due to its poor dienophilicity [38,40]. This difference can be explained by the presence of a carbonyl group conjugated to the double bond in *rac*-**2**, which significantly increases its dienophilic character. Indeed, heating *rac*-**2** with butadiene in toluene at 120 °C led to a full conversion within 12 h, affording phosphadecalone (*rac*)-*cis*-**8** in excellent yield. Further optimization revealed that microwave-assisted heating reduced the reaction time dramatically to just 2 h, maintaining high efficiency and clean product formation (Figure 3).

Detailed analysis of the crude product revealed that the reaction generated two stereoisomers: *cis*- and *trans*-fused phosphadecalones **8**. Importantly, their ratio was highly dependent on the reaction conditions. Under milder conditions, the *cis*-isomer was favored, while extended heating or higher temperatures facilitated partial isomerization to the thermodynamically more stable *trans*-isomer, likely via an enolization pathway. This finding shows that *cis*/*trans*-fusion of the cycloadduct can be selectively controlled by tuning the cycloaddition conditions.

Additionally, the molecular structure of the obtained product *trans*-**8** confirmed that the diene approaches *rac*-**2** exclusively from the P=O-bearing face (Figure 3). This *endo*-selective approach is attributed to steric hindrance from the phenyl group, effectively shielding the opposite face and enforcing a selective cycloaddition.

Having established the reactivity and selectivity of *rac*-**2** with butadiene, the subsequent investigation focused on its broader synthetic utility. This was achieved by reacting it with a panel of acyclic dienes (**4**–**7**). This approach gave access to a family of *cis*- and *trans*-fused phosphadecalones (**9**–**12**) with excellent yields and stereoselectivity, establishing *rac*-**2** as a powerful building block for the construction of diverse bicyclic heterocycles (Figure 3).

Following the successful formation of the phosphadecalone scaffold from the reaction of *rac*-**2** and butadiene, we next investigated the selectivity of the cycloaddition with other symmetric acyclic dienes. The reaction of 2,3-dimethylbutadiene (**4**) with *rac*-**2** at 110 °C afforded the corresponding cycloadduct **9** with excellent efficiency (94%), where *cis* and *trans* stereoisomers were formed in the ratio of 93:7.

In view of these promising results, a set of unsymmetrically substituted dienes was subjected to microwave-assisted cycloadditions with *rac*-**2** to broaden the structural diversity of the synthesized phosphadecalone derivatives. Under optimized conditions (toluene, 110–120 °C), the reactions proceeded smoothly, affording *cis*-fused cycloadducts **10**–**12** in excellent yields ranging from 77% to 99% (Figure 3). Notably, in the case of isoprene (**5**), the desired adduct **10** was obtained in 92% yield with a *cis*/*trans* ratio of 94:6. However, the reaction exhibited low regioselectivity, affording regioisomers **10a** and **10b** in nearly equimolar amounts (45% and 55%, respectively).

Substitution at the 1-position of butadiene was also well tolerated, allowing access to cycloadducts **11** and **12** with excellent regio- and stereocontrol, probably due to steric factors. The regio- and stereoselectivity of the cycloaddition was determined by an X-ray structural analysis of (*rac*)-*cis*-**12** crystals (Figure 3), identifying it as (*rac*)-*cis*-7-methoxy-2-phenyl-2-phosphabicyclo[4.4.0]dec-8-en-5-one 2-oxide formed via an *endo*-selective cycloaddition pathway. The structural analysis, supported by 2D NMR studies of (*rac*)-*cis*-**11** and (*rac*)-*cis*-**12**, revealed that both regioisomers result from an *endo* approach, with the methyl or methoxy substituent located on the opposite face of the bicycle relative to the hydrogen atoms and the phenyl group. Moreover, when 1,3-pentadiene (**6**) was used as the diene, the ratio of *cis*- and *trans*-fused isomers of **11** could be effectively controlled by adjusting the reaction conditions, offering direct access to either isomer with high selectivity.

**Figure 4:**
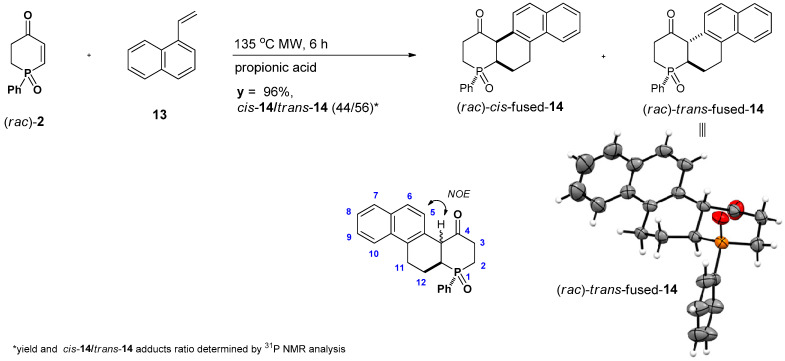
Cycloaddition reactions of 1-vinylnaphthalene (**13**) with (*rac*)-**2** as a dienophile and molecular structure of (*rac*)*-trans*-fused-**14**. The conditions under which the reaction was carried out are given.

The next stage of the study involved the reaction of (*rac*)-**2** with 1-vinylnaphthalene (**13**), under modified conditions inspired by a protocol for *trans*-benzyl-phenyl[*β*-(carbomethoxy)vinyl] phosphine oxide [42]. Using propionic acid as the solvent at 135 °C, the cycloaddition afford the phosphahexahydrochrysene derivative **14** with excellent efficiency (96% yield). Two stereoisomers, *cis*-**14** and *trans*-**14**, were obtained in a 44:56 ratio, as determined by ^31^P NMR analysis (Figure 4). Flash chromatography enabled their separation, providing a combined isolated yield of 61%.

Crystallization attempts yielded suitable single crystals only for *trans*-**14** isomer, whose molecular structure was determined (Figure 4). This analysis confirmed the *trans*-fused structure and established the relative orientation of the naphthalene moiety. The molecular structure was consistent with the NOESY data, which showed key NOE cross-peaks between the ArH-5 proton and the *α*-proton adjacent to the carbonyl group. In *cis*-**14**, the presence of strong NOE correlations was observed between the bridgehead protons, providing an indication of their *syn*-orientation across the fused ring junction.

### 2.2. Synthesis of Tricyclic Phenylphosphin-2-en-4-one Derivatives

**Figure 5:**
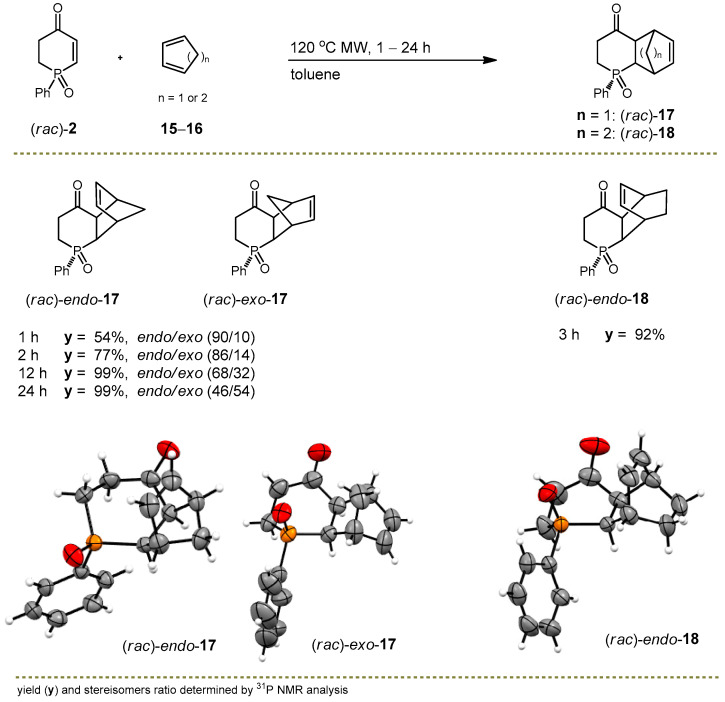
Cycloaddition reactions of cyclic dienes **15** and **16** to (*rac*)-**2** and molecular structures of products (*rac*)*-endo*-**17**, (*rac*)*-exo*-**17** and (*rac*)*-endo*-**18**. The conditions under which the reactions were carried out are given.

Cyclic dienes are highly effective for forming polycyclic skeletons via Diels–Alder reactions. Their conformational restrictions lower the entropic cost of cycloaddition, resulting in increased reactivity and improved stereocontrol compared to acyclic systems [40,43,44]. Motivated by these advantages, and following the establishment of efficient cycloaddition protocols, we decided to apply the Diels–Alder reaction to access rigid carbocyclic skeletons containing a phosphinane ring. Cyclopentadiene (**15**) and cyclohexadiene (**16**), which were successfully employed in the cycloaddition with phospholo-2-ene oxide, were selected as representative cyclic dienes for this purpose [38,39,40]. Both dienes were successfully employed in [4+2] cycloadditions with (*rac*)-**2**, affording the corresponding tricyclic phosphacycles **17** and **18** under mild conditions with excellent yields and stereocontrol (Figure 5).

The cycloaddition of (*rac*)-**2** with cyclopentadiene (**15**) at 120 °C smoothly afforded the tricyclic phosphaundecenone oxide **17** in 54% yield after 1 h. Following Alder’s rule, which states that Diels–Alder cycloadditions favor the formation of *endo*-products through maximized π-overlap, we initially observed a clear preference for the *endo*-**17** isomer in the reaction of (*rac*)-**2** with cyclopentadiene [45,46]. Interestingly, as the reaction progressed, a significant amount of the *exo*-**17** isomer also appeared. Extending the reaction time at constant temperature not only improved the yield but also altered the *endo*/*exo* isomer distribution. Complete consumption of (*rac*)-**2** was achieved after 12 h, accompanied by a marked increase in the amount of the *exo*-isomer. Prolonged heating further favored isomerization toward the thermodynamically preferred *exo*-**17**, as depicted in Figure 5.

**Figure 6:**
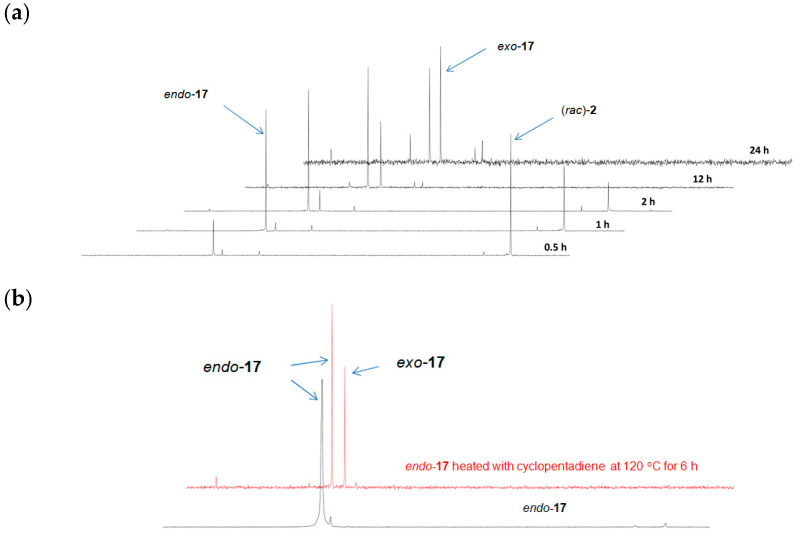
^31^P NMR spectra monitoring: (**a**) the cycloaddition of *rac*-**2** with cyclopentadiene; (**b**) conversion of *endo*-**17** to *exo*-**17**.

Intrigued by the unexpected formation of the *exo*-**17** adduct, we conducted an investigation of the cycloaddition dynamics. The ^3^^1^P NMR spectroscopy was used to monitor the stereochemical evolution of the reaction between (*rac*)-**2** and cyclopentadiene (**15**). This analysis provided valuable insights into the temporal development of the *endo*/*exo* ratio (Figure 6a).

As is often reported, although *endo*-cycloadducts are favored kinetically in Diels–Alder reactions, they can be thermally labile and undergo retro-Diels–Alder (rDA) fragmentation. This process regenerates the starting diene and dienophile under suitable conditions [47]. In contrast, *exo*-isomers, though formed more slowly, are thermodynamically stable and resistant to retro-cycloaddition.

In our case, prolonged heating of the reaction mixture led to the partial decomposition of the initially formed *endo*-**17** isomer, favoring the accumulation of the more stable *exo*-**17** isomer. The real-time monitoring of the cycloaddition process by ^3^^1^P NMR spectroscopy at 120 °C provided valuable insights into the stereochemical dynamics of the system (Figure 6a). Signals attributable to the rDA reaction emerged within 30 min, highlighting the lability of the initially formed *endo*-**17** isomer. These observations suggest that the final stereoisomeric distribution is shaped by both the initial cycloaddition kinetics and the competing re-cycloaddition processes.

To confirm that *exo*-**17** arises via the retro-Diels–Alder pathway, an independent experiment was performed. The isolated *endo*-**17** isomer was heated in the presence of excess cyclopentadiene. After heating at 120 °C for six hours, a new ^3^^1^P NMR signal corresponding to the thermodynamically favored *exo*-**17** appeared (Figure 6b), corroborating the proposed mechanism.

**Figure 7:**
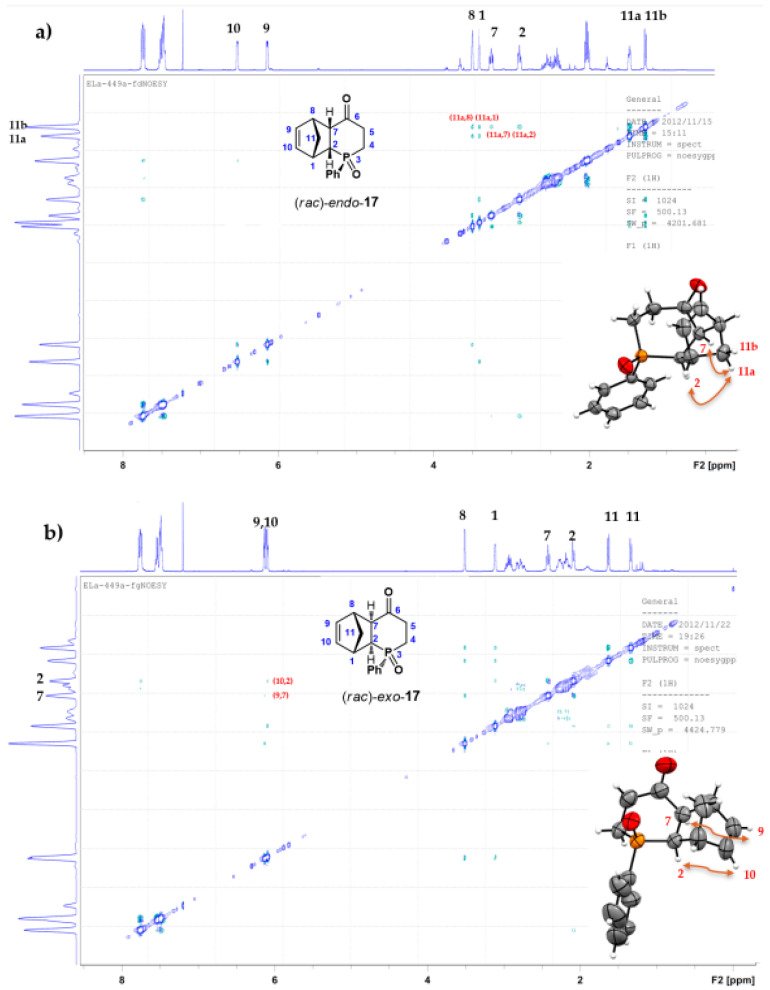
The 500 MHz ^1^H 2D NOESY spectrum and the key NOE interactions for: (**a**) (*rac*)*-endo*-**17**; (**b**) (*rac*)*-exo*-**17**.

The *endo*- and *exo*-isomers of cycloadduct **17** were independently validated by X-ray crystallography and NOESY spectroscopy. The study of the molecular structures confirmed the relative stereochemistry (Figure 5), while NOE cross-peaks—H-11a/H-2 and H-11a/H-7 for *endo*-**17** (Figure 7a), and H-7/H-9 and H-2/H-10 for *exo*-**17** (Figure 7b)—unambiguously supported the structural assignments.

Encouraged by the efficient formation of tricyclic phosphinane derivatives with cyclopentadiene, we next turned our attention to 1,3-cyclohexadiene (**16**) as the diene partner. When (*rac*)-**2** was reacted with **16** at 120 °C, the tricyclic phosphadodecenone oxide **18** was obtained with an isolated yield of 91% (Figure 5). Remarkably, the reaction displayed outstanding stereocontrol, furnishing almost exclusively a single stereoisomer. The molecular structure of the obtained tricyclic product (*rac*)-**18** confirmed the preferential *endo* approach of diene **16** toward the phosphorus dienophile (*rac*)-**2**, selectively occurring from the face opposite to the phenyl substituent, consistent with steric demands.

Unfortunately, further attempts to expand the scope of cyclic dienes in this reaction were unsuccessful. No cycloadduct formation was observed with five-membered heterocyclic dienes, such as furan and 2,5-diiodothiophene, nor with 1,3-cyclooctadiene under standard conditions (3 h, 120 °C). When α-phellandrene, a six-membered cyclic diene, was used, the reaction proceeded with 38% conversion of (*rac*)-**2**, but the resulting product was obtained as a complex mixture of five inseparable isomers.

### 2.3. Synthesis of Bicyclic P-Stereogenic Phosphadecalones

The strategy for the synthesis of bicyclic and polycyclic phosphines presented in this work is characterized by its high versatility and efficiency, enabling the preparation of a wide variety of structurally diverse compounds. This methodology not only advances the field of phosphadecalone chemistry but also paves the way toward novel P-stereogenic frameworks that had remained unexplored.

**Figure 8:**
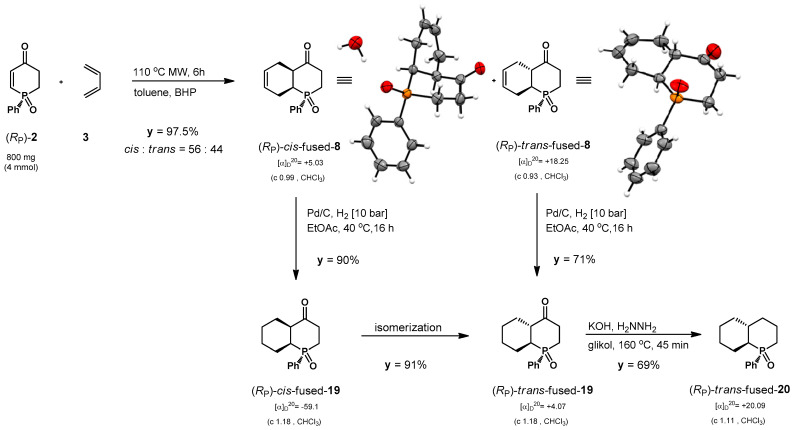
Synthesis of enantiomerically pure *P*-sterogenic phosphadecalenones (*R*_P_)-**8** and their derivatives (*R*_P_)-**19** and (*R*_P_)-**20**. The conditions under which the reactions were carried out are given.

To demonstrate the versatility of this approach, an enantiomerically pure P-stereogenic phosphadecalone was obtained via Diels–Alder cycloaddition of enantiopure dienophile (*R*_P_)-**2** with butadiene (**3**) under microwave irradiation at 110 °C (Figure 8). After 6 h, the reaction afforded a 56:44 mixture of (*R*_P_)-*cis*- and (*R*_P_)-*trans*-2-phenyl-2-phosphabicyclo[4.4.0]dec-8-en-5-one 2-oxide (**8**) in an excellent 97.5% isolated yield. The two diastereomers were efficiently separated by flash chromatography.

The expected *cis* and *trans* fusion, along with the absolute *R*-configuration at phosphorus, was confirmed by X-ray crystallography for both isomers (Figure 8). It is worth noting that six-membered phosphorus heterocycles typically adopt the ring conformations commonly found for their carbocyclic counterparts. Moreover, changes in the stereochemistry of the bridgehead carbon, as seen in the molecular structure images of both isomers, can significantly impact the overall molecular shape and, by extension, the properties and reactivity of these *P*-chiral phosphines.

In view of their structural versatility, both isolated isomers of (*R*_P_)-**8** were further explored as synthetic intermediates and subjected to a range of chemical modifications, as shown in Figure 8.

The double bond present in both isomers of (*R*_P_)-**8** was selectively reduced under mild conditions to afford the optically pure *cis*- and *trans*-fused phosphadecalones **19** in excellent and good yields, respectively. Furthermore, the thermal isomerization of (*R*_P_)-*cis*-**19** enabled efficient access to the corresponding (*R*_P_)-*trans*-**19** isomer in nearly quantitative yield, without loss of enantiomeric purity. Finally, deoxygenation of (*R*_P_)-*trans*-**19** via Wolff–Kishner reduction under basic conditions yielded the corresponding phosphadaline **20** in 69% yield. These transformations underscore the high synthetic versatility of the phosphadecalone framework and open new avenues for accessing structurally complex *P*-chiral phosphorus heterocycles.

## 3. Materials and Methods

### 3.1. General Information

All reagents were purchased from commercial suppliers and used without further purification unless otherwise stated. Only dry solvents were used, and all glassware was flame dried under vacuum prior to use. Solvents for chromatography and extraction were used as received, while solvents for crystallization and triethylamine (Et_3_N) were distilled once before use. The *rac*-1-phenylphosphin-2-en-4-one 1-oxide (**2**) [37] and (*R*_P_)-1-phenylphosphin-2-en-4-one 1-oxide ((*R*_P_)-**2**) [41] were prepared according to the literature procedures. Microwave-assisted reactions were performed using a single-mode Discover LabMate System (CEM Corp. Matthews, NC, USA) with standard Pyrex or quartz vessels (10 or 25 mL capacity) operating in temperature-control mode. The temperature was monitored by a built-in calibrated infrared (IR) sensor.

NMR spectra were recorded on a Bruker Ascend (500 MHz) spectrometer (Bruker Corp., Rheinstetten, Germany; software: TopSpin 4.2.1) using CDCl_3_ as a solvent at room temperature unless otherwise noted. Chemical shifts (δ) are given in ppm relative to tetramethylsilane (^1^H), with residual CHCl_3_ (^13^C) as a reference. Coupling constants (*J*) are given in Hz. The following abbreviations of signal patterns are as follows: s (singlet), d (doublet), t (triplet), q (quartet), m (multiplet), br (broad). Optical rotations were measured on a PerkinElmer 341LC polarimeter (Waltham, MA, USA) using a 1 mL cell with a 10 mm path length at ambient temperature and are reported as follows: [α]_D_^20^ (c g/100 mL, solvent). Melting points were determined using a Büchi M-560 (Büchi Labortechnik AG, Flawil, Switzerland) melting point apparatus in open capillaries and are uncorrected. Elementary analyses (C, H, N) were performed on a PerkinElmer CHN 2400 analyzer (PerkinElmer, Inc., Rodgau, Germany). Chiral HPLC analysis was performed on a Shimadzu HPLC (Shimadzu Corp., Kyoto, Japan; software: LabSolutions 5.93) system equipped with Chiralcel^®^ columns. Thin layer chromatography (TLC) was performed with precoated silica gel plates (Kieselgel 60, F254 on aluminum sheet, Merck sp. z o.o., Warszawa, Poland) and visualized by potassium permanganate (KMnO_4_) stain or exposure to iodine vapor. Column chromatography was conducted using silica gel 60 (230–400 mesh) (Merck) unless noted otherwise.

### 3.2. X-Ray Crystallography

The single crystal diffraction data were collected at room temperature with a SuperNova diffractometer (Oxford Diffraction; Agilent Technologies, Yarnton, UK [48]) using graphite monochromated CuKα radiation (λ = 1.54184 Å). The CrysAlisPro program system [49] was used for data collection, cell refinement, and data reduction. The intensities were corrected for Lorentz and polarization effects, and a multi-scan absorption corrections were applied. The crystal structure was solved by direct methods using the SHELXT program and refined by the full-matrix least squares method on F^2^ using the SHELXL-2018/3 program [50,51]. The non-hydrogen atoms were refined with anisotropic displacement parameters, H-atoms were positioned at calculated positions and refined using the riding model. The experimental details and final atomic parameters for the analyzed crystals were deposited with the Cambridge Crystallographic Data Centre as Supplementary Material (CCDC Nos 2300707–2300714). These data are provided free of charge by the Cambridge Crystallographic Data Centre Access Structures service www.ccdc.cam.ac.uk/structures/?.

### 3.3. Synthesis and Spectral Data

The general procedure for the microwave-assisted Diels–Alder cycloaddition of 1-phenylphosphin-2-en-4-one 1-oxide (**2**) with dienes is as follows:

In an oven-dried 10 mL pressure-rated reaction vial equipped with a magnetic stirring bar, (*rac*)- or (*R*_P_)-1-phenylphosphin-2-en-4-one 1-oxide (**2**) (typically 0.75 mmol) was dissolved in dry toluene (1 mL) or propanoic acid (1 mL). Diene (3.0–9.0 mmol) and a stabilizer (BHT or hydroquinone HQ, 0.05–0.1 mol%) were then added. The resulting reaction mixture was stirred at the specified temperature (110–140 °C) under microwave irradiation until complete consumption of the starting material (1–6 h). After completion, the solvent was removed under reduced pressure, and the crude product was purified by flash column chromatography (CHCl_3_/THF) to afford the corresponding cycloadduct.

#### 3.3.1. Synthesis of *rel*-(*S*_P_,1*R*,6*S*)-2-Phenyl 2-phosphabicyclo[4.4.0]dec-8-en-5-one 2-Oxide ((*rac*)-*cis*-**8**) and *rel*-(*S*_P_,1*R*,6*R*)-2-Phenyl 2-phosphabicyclo[4.4.0]dec-8-en-5-one 2-Oxide ((*rac*)-*trans*-**8**): General Procedure

Cycloadduct **8** was obtained as a mixture of *cis* and *trans* isomers according to the general procedure, using (*rac*)-1-phenylphosphin-2-en-4-one 1-oxide ((*rac*)-**2**) (150 mg, 0.75 mmol) and butadiene (approximately 0.5 mL, 9 mmol). To prevent polymerization the 0.05% mol of BHT was added. The reaction was carried out at 110 °C for 2 h under microwave irradiation. Both isomeric fractions were separated and fully characterized. Conversion and isomeric ratios were determined by ^31^P NMR measurements as described in Figure 3. The crude product was purified by silica gel chromatography (CHCl_3_/THF = 10.5:0.5 to 8:1) to afford (*rac*)-*cis*-**8** (153.5 mg) as a colorless oil and (*rac*)-*trans*-**8** (25 mg) as colorless crystals (total yield: 92%).

*rel*-(*S*_P_,1*R*,6*S*)-2-Phenyl-2-phosphabicyclo[4.4.0]dec-8-en-5-one 2-oxide ((*rac*)-*cis*-**8**); colorless oil; R_f_ = 0.19 (CHCl_3_/THF = 10:1); ^1^H NMR (500 MHz, CDCl_3_) δ ppm: 2.1 (dd, *J* = 17.5, 1.4 Hz, 1 H), 2.20–2.30 (m, 1 H) 2.44–2.65 (m, 3 H) 2.65–2.77 (m, 2 H) 2.85 (spt, *J* = 5.50 Hz, 1 H) 2.95–3.04 (m, 2 H) 5.70–5.80 (m, 2 H) 7.55–7.60 (m, 2 H) 7.60–7.65 (m, 1 H) 7.84–7.89 (m, 2 H); ^13^C NMR (126 MHz, CDCl_3_) δ ppm: 22.41 (d, *J* = 2.72 Hz) 24.38 (s) 24.86 (d, *J* = 9.08 Hz) 34.69 (s) 35.20 (d, *J* = 4.54 Hz) 45.60 (d, *J* = 2.72 Hz) 123.99 (d, *J* = 8.17 Hz) 125.24 (s) 129.27 (d, *J* = 11.81 Hz) 130.00 (d, *J* = 9.08 Hz) 131.49 (d, *J* = 96.28 Hz) 132.54 (d, *J* = 2.73 Hz) 207.68 (d, *J* = 6.36 Hz); ^31^P NMR (202 MHz, CDCl_3_) δ ppm: 32.34 (s); Elemental Anal. Calcd for C_15_H_17_O_2_P: C, 69.22; H, 6.58 Found: C, 68.79; H, 6.73.

*rel*-(*S_P_*,1*R*,6*R*)-2-Phenyl-2-phosphabicyclo[4.4.0]dec-8-en-5-one 2-oxide ((*rac*)-*trans*-**8**); colorless crystals, mp = 161.8–162.9 °C; R_f_ = 0.25 (CHCl_3_/THF = 10:1); ^1^H NMR (500 MHz, CDCl_3_) δ ppm: 1.77–1.86 (m, 1 H) 2.25–2.33 (m, 3 H) 2.38–2.48 (m, 2 H) 2.62–2.73 (m, 1 H) 2.82 (ddt, *J* = 28.50, 13.56, 4.40, 4.40 Hz, 1 H) 3.18–3.26 (m, 1 H) 3.32–3.41 (m, 1 H) 5.59–5.64 (m, 1 H) 5.70–5.75 (m, 1 H) 7.52–7.57 (m, 2 H) 7.58–7.62 (m, 1 H) 7.74–7.80 (m, 2 H); ^13^C NMR (126 MHz, CDCl_3_) δ ppm: 22.95 (d, *J* = 3.63 Hz) 24.47 (d, *J* = 8.17 Hz) 28.01 (d, *J* = 63.58 Hz) 36.49 (d, *J* = 6.36 Hz) 38.99 (d, *J* = 68.12 Hz) 44.98 (d, *J* = 4.54 Hz) 124.12 (d, *J* = 11.81 Hz) 125.06 (s) 128.98 (d, *J* = 10.90 Hz) 129.67 (d, *J* = 97.18 Hz) 130.61 (d, *J* = 9.99 Hz) 132.46 (d, *J* = 2.73 Hz) 208.63 (d, *J* = 7.27 Hz); ^31^P NMR (202 MHz, CDCl_3_) δ ppm: 30.14 (s); Elemental Anal. Calcd for C_15_H_17_O_2_P: C, 69.22; H, 6.58 Found: C, 68.74; H, 6.55.

Crystal data for (*rac*)-*trans*-**8**: C_15_H_17_O_2_P, Mw = 260.25, crystal system monoclinic, space group *P*2_1_/*n*, unit cell dimensions a = 5.3416(1) Å, b = 16.0701(3) Å, c = 15.4024(3) Å, β = 92.607(2)°, V = 1320.77(4) Å^3^, Z = 4. Density (calc) 1.309 g/cm^3^, absorption coeff. 1.771 mm^−1^, F(000) = 552. Collected/independent reflections 8448/2594 [R(int) = 0.0238], data/parameters 2594/163. Goodness-of-fit on *F*^2^ 1.052, final R indices [I>2σ(I)] R1 = 0.0366, wR2 = 0.0953. CCDC No. 2300709

#### 3.3.2. Synthesis of *rel*-(*S*_P_,1*R*,6*S*)-8,9-Dimethyl-2-phenyl-2-phosphabicyclo[4.4.0]dec-8-en-5-one 2-Oxide ((*rac*)-*cis*-**9**): General Procedure

Cycloadduct **9** was obtained as a mixture of *cis* and *trans* isomers according to the general procedure, using *rac*-**2** (150 mg, 0.75 mmol), 2,4-dimethyl-1,3-butadiene (370 mg, 4.5 mmol) and BHT (0.05% mol). The reaction was carried out at 110 °C for 2 h under microwave irradiation. The only *cis* isomer was separated and characterized. Conversion and isomer ratios were determined by ^31^P NMR measurements as described in Figure 3. The crude product was purified by silica gel chromatography (CHCl_3_/THF = 10.5:0.5 to 8:1) followed by crystallization (EtOAc/hexane = 2:5) to afford *cis*-**9** (188 mg, 87%, purity 94%) as a colorless oil; R_f_ = 0.24 (CHCl_3_/THF = 10:1); ^1^H NMR (500 MHz, CDCl_3_) δ ppm 1.63 (s, 3 H) 1.66 (s, 3 H) 1.93–2.01 (m, 1 H) 2.12–2.24 (m, 1 H) 2.35–2.44 (m, 1 H) 2.45–2.71 (m, 4 H) 2.81–2.99 (m, 3 H) 7.54–7.64 (m, 3 H) 7.83–7.89 (m, 2 H); ^13^C NMR (126 MHz, CDCl_3_) δ ppm 18.90 (s) 24.24 (d, *J* = 63.58 Hz) 28.39 (d, *J* = 2.72 Hz) 30.76 (d, *J* = 8.17 Hz) 35.63 (d, *J* = 67.21 Hz) 35.47 (d, *J* = 5.45 Hz) 46.00 (d, *J* = 2.72 Hz) 122.86 (d, *J* = 9.08 Hz) 124.16 (d, *J* = 1.82 Hz) 129.27 (d, *J* = 11.81 Hz) 129.91 (d, *J* = 9.08 Hz) 131.61 (d, *J* = 95.37 Hz) 132.51 (d, *J* = 2.73 Hz) 207.68 (d, *J* = 5.45 Hz); ^31^P NMR (202 MHz, CDCl_3_) δ ppm 32.66 (s); Elemental Anal. Calcd for C_17_H_21_O_2_P: C, 70.82; H, 7.34 Found: C, 65.4; H, 7.56.

#### 3.3.3. Synthesis of *rel*-(*S*_P_,1*R*,6*S*)-8-Methyl-2-phenyl-2-phosphabicyclo[4.4.0]dec-8-en-5-one 2-Oxide ((*rac*)-*cis*-**10a**) and *rel*-(*S*_P_,1*R*,6*S*)-9-Methyl-2-phenyl-2-phosphabicyclo[4.4.0]dec-8-en-5-one 2-Oxide ((*rac*)-*cis*-**10b**)

Cycloadducts *rac*-**10a** and *rac*-**10b** were obtained as a mixture of two regioisomers (ratio = 45:55) according to the general procedure, using *rac*-**2** (150 mg, 0.75 mmol), isoprene (310 mg, 4.5 mmol) and BHT (0.05% mol). The reaction was carried out at 110 °C for 6 h under microwave irradiation. The only *cis* isomer was separated and characterized. Conversion and isomer ratios were determined by ^31^P NMR measurements as described in Figure 3. The crude mixture was purified by silica gel chromatography (CHCl_3_/THF = 10.5:0.5 to 8:1) to afford the mixture of two inseparable cycloadducts *rac*-**10a** and *rac*-**10b** (168 mg) as a white sticky solid (total yield: 83%; R_f_ = 0.21 (CHCl_3_/THF = 10:1);

*rel*-(*S_P_*,1*R*,6*S*)-8-Methyl-2-phenyl-2-phosphabicyclo[4.4.0]dec-8-en-5-one 2-oxide ((*rac*)-*cis*-**10a**); white sticky solid; ^1^H NMR (500 MHz, CDCl_3_) δ ppm 1.7 (s, 3 H), 1.8 (d, *J* = 17.3 Hz, 1 H), 2.1–2.5 (m, 5 H), 2.6–2.7 (m, 1 H), 2.7–2.9 (m, 1 H), 3.2–3.3 (m, 1 H), 3.3–3.4 (m, 1 H), 5.3 (s, 1 H), 7.5–7.6 (m, 3 H), 7.7–7.8 (m, 2 H); ^13^C NMR (126 MHz, CDCl_3_) δ ppm 23.2 (d, *J* = 3.6 Hz), 23.5 (s), 27.8 (s), 29.2 (d, *J* = 9.1 Hz), 36.5 (s), 38.8 (d, *J* = 66.8 Hz), 45.4 (d, *J* = 4.5 Hz), 118.0 (d, *J* = 12.2 Hz), 129.0 (d, *J* = 10.8 Hz), 129.7 (d, *J* = 96.6 Hz), 130.6 (d, *J* = 9.1 Hz), 132.3 (s), 132.4 (d, *J* = 2.7 Hz), 208.7 (s); ^31^P NMR (202 MHz, CDCl_3_) δ ppm 30.7 (s).

*rel*-(*S_P_*,1*R*,6*S*)-9-Methyl-2-phenyl-2-phosphabicyclo[4.4.0]dec-8-en-5-one 2-oxide ((*rac*)-*cis*-**10b**); white sticky solid; ^1^H NMR (500 MHz, CDCl_3_) δ ppm 1.58 (s, 3 H), 1.64 (d, *J* = 17.65 Hz, 1 H), 2.09–2.48 (m, 5 H), 2.57–2.70 (m, 1 H), 2.74–2.89 (m, 1 H), 3.05–3.17 (m, 1 H), 3.30–3.41 (m, 1 H), 5.42 (br. s., 1 H), 7.50–7.64 (m, 3 H), 7.72–7.82 (m, 2 H); ^13^C NMR (126 MHz, CDCl_3_) δ ppm 23.0 (s), 24.9 (d, *J* = 9.1 Hz), 27.5 (d, *J* = 3.6 Hz), 28.3 (s), 36.5 (s), 39.3 (d, *J* = 68.6 Hz), 44.9 (d, *J* = 4.5 Hz), 119.2 (s), 129.0 (d, *J* = 10.9 Hz), 129.6 (d, *J* = 95.4 Hz), 130.6 (d, *J* = 9.1 Hz), 131.3 (d, *J* = 10.9 Hz), 132.5 (d, *J* = 2.7 Hz), 208.8 (s); ^31^P NMR (202 MHz, CDCl_3_) δ ppm 30.5 (s).

#### 3.3.4. Synthesis of *rel*-(*S*_P_,1*R*,6*S*,7*S*)-7-Methyl-2-phenyl-2-phosphabicyclo[4.4.0]dec-8-en-5-one 2-Oxide ((*rac*)-*cis*-**11**) and *rel*-(*S*_P_,1*R*,6*R*,7*S*)-7-Methyl-2-phenyl-2-phosphabicyclo[4.4.0]dec-8-en-5-one 2-Oxide ((*rac*)-*trans*-**11**)

Cycloadduct *(rac)-cis*-**11** was obtained according to the general procedure, using *rac*-**2** (150 mg, 0.75 mmol), 1,3-pentadiene (0.61 mL, 9 mmol) and BHT (0.05% mol). The reaction was carried out at 120 °C for 2 h under microwave irradiation. The crude product was purified by silica gel chromatography (CHCl_3_/THF = 10.5:0.5 to 8:1) followed by crystallization (EtOAc/hexane = 2:5) to afford *(rac)-cis*-**11** (123 mg, 62%) as a yellowish thick oil; R_f_ = 0.2 (CHCl_3_/THF = 10:1); ^1^H NMR (500 MHz, CDCl_3_) δ ppm 1.08 (d, *J* = 7.57 Hz), 2.14–2.26 (m), 2.26–2.35 (m), 2.43–2.73 (m), 2.83 (dt, *J* = 7.20, 3.90 Hz), 2.89–3.00 (m), 5.51–5.57 (m), 5.61 (ddt, *J* = 10.09, 4.89, 2.60, 2.60 Hz), 7.51–7.61 (m), 7.80–7.86 (m); ^13^C NMR (126 MHz, CDCl_3_) d ppm 17.72 (d, *J* = 1.82 Hz) 22.49 (d, *J* = 2.72 Hz) 24.05 (d, *J* = 61.76 Hz) 32.90 (d, *J* = 9.08 Hz) 37.33 (d, *J* = 4.54 Hz) 38.14 (d, *J* = 66.30 Hz) 49.15 (d, *J* = 2.72 Hz) 122.60 (d, *J* = 11.81 Hz) 129.45 (d, *J* = 10.90 Hz) 129.72 (d, *J* = 9.08 Hz) 131.37 (d, *J* = 97.80 Hz) 131.46 (s) 132.69 (d, *J* = 2.73 Hz) 206.03 (d, *J* = 5.45 Hz); ^31^P NMR (202 MHz, CDCl_3_) δ ppm 33.07 (s) Elemental Anal. Calcd for C_16_H_19_O_2_P: C, 70.06; H, 6.98 Found: C, 69.98; H, 7.02.

Cycloadduct *(rac)-trans*-**11** was obtained according to the general procedure, using *rac*-**2** (150 mg, 0.75 mmol), 1,3-pentadiene (0.61 mL, 9 mmol) and BHT (0.05% mol). The reaction was carried out at 140 °C for 6 h under microwave irradiation. The crude product was purified by silica gel chromatography (CHCl_3_/THF = 10.5:0.5 to 8:1) followed by crystallization (EtOAc/hexane = 2:5) to afford *(rac)-trans*-**11** (182 mg, 89%) as a colorless crystals, mp = 188.0–188.8 °C; R_f_ = 0.31 (CHCl_3_/THF = 10:1); ^1^H NMR (500 MHz, CDCl_3_) δ ppm 1.00 (d, *J* = 6.94 Hz, 3 H) 1.73 (d, *J* = 17.65 Hz, 1 H) 2.22–2.36 (m, 2 H) 2.39–2.49 (m, 1 H) 2.55–2.67 (m, 1 H) 2.70–2.83 (m, 2 H) 2.96 (ddd, *J* = 13.32, 9.38, 4.41 Hz, 1 H) 3.29–3.41 (m, 1 H) 5.45–5.50 (m, 1 H) 5.51–5.56 (m, 1 H) 7.48–7.55 (m, 2 H) 7.56–7.62 (m, 1 H) 7.70–7.78 (m, 2 H); ^13^C NMR (126 MHz, CDCl_3_) δ ppm 20.87 (s) 22.54 (d, *J* = 3.63 Hz) 28.90 (s) 29.25 (d, *J* = 64.49 Hz) 37.19 (d, *J* = 5.45 Hz) 40.00 (d, *J* = 69.94 Hz) 51.89 (d, *J* = 4.54 Hz) 122.44 (d, *J* = 12.72 Hz) 128.91 (s) 129.55 (d, *J* = 95.37 Hz) 130.59 (d, *J* = 9.08 Hz) 131.93 (s) 132.45 (d, *J* = 1.82 Hz) 209.05 (d, *J* = 6.36 Hz); ^31^P NMR (202 MHz, CDCl_3_) δ ppm 30.84 (s); Elemental Anal. Calcd for C_16_H_19_O_2_P: C, 70.06; H, 6.98 Found: C, 70.01; H, 7.04.

#### 3.3.5. Synthesis of *rel*-(*S*_P_,1*R*,6*S*,7*S*)-7-Methoxy-2-phenyl-2-phosphabicyclo[4.4.0]dec-8-en-5-one 2-Oxide ((*rac*)-*cis*-**12**)

Cycloadduct *rac*-**12** was obtained as a mixture of *cis* and *trans* isomers according to the general procedure, using *rac*-**2** (150 mg, 0.75 mmol), 1-methoxy-1,3-butadiene (370 mg, 4.5 mmol) and BHT (0.05% mol). The reaction was carried out at 120 °C for 2 h under microwave irradiation. The only *cis* isomer was separated and characterized. Conversion and isomer ratios were determined by ^31^P NMR as described in Figure 3. The crude product was purified by silica gel chromatography (CHCl_3_/THF = 10.5:0.5 to 8:1) followed by crystallization (acetone/hexane = 2:5) to afford (*rac*)-*cis*-**12** (105 mg, 49%) as a colorless crystals; mp = 179.6–180.8 °C; R_f_ = 0.14 (CHCl_3_/THF = 10:1); ^1^H NMR (500 MHz, CDCl_3_) δ ppm 2.42–2.51 (m, 2 H) 2.57–2.80 (m, 3 H) 2.81–2.94 (m, 2 H) 3.15 (dt, *J* = 19.86, 5.04 Hz, 1 H) 3.39 (s, 3 H) 4.04 (dd, *J* = 2.21, 1.58 Hz, 1 H) 5.89–5.96 (m, 1 H) 6.05 (dd, *J* = 10.40, 3.15 Hz, 1 H) 7.54–7.67 (m, 3 H) 7.86–7.93 (m, 2 H); ^13^C NMR (126 MHz, CDCl_3_) δ ppm 22.44 (d, *J* = 2.72 Hz) 24.93 (d, *J* = 62.67 Hz) 33.38 (d, *J* = 65.40 Hz) 37.84 (d, *J* = 5.45 Hz) 49.14 (d, *J* = 3.63 Hz) 56.72 (s) 74.02 (d, *J* = 7.27 Hz) 125.64 (s) 127.26 (d, *J* = 6.36 Hz) 129.20 (d, *J* = 10.90 Hz) 129.90 (d, *J* = 9.08 Hz) 132.32 (d, *J* = 2.73 Hz) 133.19 (d, *J* = 95.37 Hz) 206.43 (d, *J* = 5.45 Hz); ^31^P NMR (202 MHz, CDCl_3_) δ ppm 30.12 (s); Calcd for C_16_H_19_O_3_P: C, 66.20; H, 6.60 Found: C, 61.95; H, 6.55.

Crystal data for (*rac*)-*cis*-**12**: C_32_H_38_O_6_P_2_, Mw = 580.56, crystal system monoclinic, space group *Pc*, unit cell dimensions a = 7.3147(5) Å, b = 18.0838(8) Å, c = 11.1951(6) Å, β = 90.556(5)°, Volume = 1480.79(14) Å^3^, Z = 2, density (calc) 1.302 g/cm^3^, absorption coeff. 1.686 mm^−1^, F(000) = 616. Collected/independent reflections 9963/5411 [R(int) = 0.0445], Data/restraints/parameters 5411/2/361, goodness-of-fit on F^2^ 1.086, final R indices [I>2σ(I)] R1 = 0.0708, wR2 = 0.1846. Absolute structure parameter 0.03(3). CCDC No. 2300710.

#### 3.3.6. Synthesis of *rel*-(*S*_P_,4a*R*,12a*R*)-1-Phenyl-1-phospha-2,3,11,12,12a-hexahydrochrysen-4-one 1-Oxide ((*rac*)-*cis*-**14**) and *rel*-(*S*_P_,4a*S*,12a*R*)-1-Phenyl-1-phospha-2,3,11,12,12a-hexahydrochrysen-4-one 1-Oxide ((*rac*)-*trans*-**14**)

Cycloadduct of *rac*-**2** and 1-vinyl-nafthalene were obtained as a mixture of two isomers (*rac*)-*cis*-**14** and (*rac*)-*trans*-**14** according to the general procedure, using *rac*-**2** (206 mg, 1 mmol), 1-vinyl-nafthalene (600 mg, 3.9 mmol), propanoic acid (1 mL) and HQ (25 mg, 0.23 mmol). The reaction was carried out at 135 °C for 6 h under microwave irradiation. Both isomers could be separated and characterized. Conversion and isomer ratios were determined by ^31^P NMR as described in Figure 4. After the reaction, the pH of the mixture was adjusted to 10 with 20% NaOH, and the solution was extracted with CHCl_3_. The combined organic phases were dried over MgSO_4_, filtered, and the solvent was removed under reduced pressure. Purification by silica gel chromatography (CHCl_3_/THF = 10.5:0.5 to 8:1) afforded both pure isomers (*rac*)-*cis*-**14** (37 mg) as a thick oil and (*rac*)-*trans*-**14** (75 mg) as colorless crystals. Total yield: 61%.

(*rac*)-*cis*-**14**: yellow thick oil; R_f_ = 0.16 (CHCl_3_/THF = 10:1); ^1^H NMR (500 MHz, CDCl_3_) δ ppm 1.90–2.00 (m, 1 H) 2.53–2.66 (m, 1 H) 2.76–2.88 (m, 3 H) 2.90–3.00 (m, 1 H) 3.09–3.22 (m, 2 H) 3.57 (dd, *J* = 17.50, 5.83 Hz, 1 H) 4.10 (t, *J* = 4.89 Hz, 1 H) 6.97 (d, *J* = 8.51 Hz, 1 H) 7.49–7.56 (m, 2 H) 7.60–7.70 (m, 4 H) 7.82–7.85 (m, 1 H) 7.93–7.98 (m, 2 H) 7.98–8.02 (m, 1 H); ^13^C NMR (126 MHz, CDCl_3_) δ ppm ^13^C NMR (126 MHz, CDCl_3_) d ppm 20.2 (d, *J* = 2.7 Hz), 24.1 (d, *J* = 63.4 Hz), 25.7 (d, *J* = 10.9 Hz), 36.2 (d, *J* = 5.4 Hz), 36.9 (d, *J* = 66.7 Hz), 53.3 (s), 123.1 (s), 125.9 (s), 126.3 (d, *J* = 20.0 Hz), 128.1 (d, *J* = 1.2 Hz), 128.6 (s), 129.0 (d, *J* = 9.1 Hz), 129.2 (d, *J* = 12.7 Hz), 129.5 (d, *J* = 11.8 Hz), 129.9 (d, *J* = 9.1 Hz), 131.3 (d, *J* = 96.0 Hz), 132.0 (d, *J* = 37.2 Hz), 132.8 (d, *J* = 2.5 Hz),132.9 (s), 138.2 (d, *J* = 83.5 Hz), 206.8 (d, *J* = 7.3 Hz); ^31^P NMR (202 MHz, CDCl_3_) δ ppm 31.45 (s); Calcd for C_23_H_21_O_2_P: C, 76.65; H, 5.87 Found: C, 76.33; H, 5.99.

(*rac*)-*trans*-**14**: colorless crystals, mp = 231.3–234.8 °C; R_f_ *=* 0.24 (CHCl_3_/THF = 10:1); ^1^H NMR (500 MHz, CDCl_3_) δ ppm 1.92–2.00 (m, 1 H) 2.06–2.17 (m, 1 H) 2.33–2.47 (m, 2 H) 2.47–2.59 (m, 1 H) 2.81–2.95 (m, 2 H) 3.31 (d, *J* = 17.02 Hz, 1 H) 3.41–3.52 (m, 1 H) 5.09 (dd, *J* = 12.77, 9.30 Hz, 1 H) 7.19 (d, *J* = 8.51 Hz, 1 H) 7.45–7.54 (m, 2 H) 7.57–7.63 (m, 2 H) 7.64–7.69 (m, 1 H) 7.72 (d, *J* = 8.83 Hz, 1 H) 7.81–7.91 (m, 4 H); ^13^C NMR (126 MHz, CDCl_3_) δ ppm 20.92 (d, *J* = 3.63 Hz) 25.13 (d, *J* = 13.62 Hz) 29.36 (d, *J* = 62.67 Hz) 38.02 (d, *J* = 5.45 Hz) 42.06 (d, *J* = 71.75 Hz) 50.64 (d, *J* = 3.63 Hz) 122.94 (s) 125.74 (s) 125.89 (s) 126.07 (s) 128.10 (d, *J* = 9.08 Hz) 128.24 (s) 128.47 (s) 129.01 (d, *J* = 97.18 Hz) 129.16 (d, *J* = 11.81 Hz) 130.84 (d, *J* = 9.08 Hz) 132.07 (s) 132.18 (d, *J* = 1.82 Hz) 132.44 (s) 132.71 (d, *J* = 2.72 Hz); ^31^P NMR (202 MHz, CDCl_3_) δ ppm 33.01 (s); Calcd for C_23_H_21_O_2_P: C, 76.65; H, 5.87 Found: C, 76.52; H, 5.91.

Crystal data for (*rac*)-*trans*-**14**: C_23_H_21_O_2_P, Mw = 360.37, crystal system triclinic, space group P-1, unit cell dimensions a = 7.8011(5) Å, b = 9.7862(6) Å, c = 13.6324(9) Å, α = 74.592(6)°, β = 80.724(6)°, γ = 67.013(6)°, V = 921.73(11) Å^3^, Z = 2, Density (calc) = 1.298 g/cm^3^, absorption coefficient 1.425 mm^−1^, F(000) = 380. Collected/independent reflections 6338/3552 [R(int) = 0.0292], data/parameters 3552/235, goodness-of-fit on F^2^ 1.060, final R indices [I>2σ(I)] R1 = 0.0398, wR2 = 0.1035. CCDC No. 2300711.

#### 3.3.7. Synthesis of *rel*-(*S*_P_,1*S*,2*R*,7*S*,8*R*)-3-Phenyl-3-phosphatricyclo[6.2.1.0^2,7^]undec-9-en-6-one 3-Oxide ((*rac*)-*endo*-**17**) and *rel*-(S_P_,1*R*,*2R*,7*S*,8*S*)-3-Phenyl-3-phosphatricyclo[6.2.1.0^2,7^]undec-9-en-6-one 3-Oxide ((*rac*)-*exo*-**17**)

Cycloadduct **17** was obtained as a mixture of stereoisomers *exo*-**17** and *endo*-**17** according to the general procedure, using *rac*-**2** (150 mg, 0.75 mmol), dicyclopentadiene (395 mg, 3 mmol) and BHT (0.05% mol). The reaction was carried out at 120 °C for 12 h under microwave irradiation. Both stereoisomers were separated and characterized. Conversion and stereoisomer ratios were determined by ^31^P NMR as described in Figure 5. The crude products were purified by silica gel chromatography (CHCl_3_/THF = 10.5:0.5 to 8:1) to afford product (*rac*)*-endo*-**17** (95 mg) and product (*rac*)*-exo*-**17** (72 mg), (total yield: 84%).

(*rac*)*-endo*-**17**; colorless crystals; mp = 119.8–123.1 °C; R_f_ = 0.15 (CHCl_3_/THF = 10:1); ^1^H NMR (500 MHz, CDCl_3_) δ ppm 1.33 (d, *J* = 8.83 Hz, 5 H) 1.50–1.56 (m, 5 H) 2.04–2.12 (m, 2 H) 2.41–2.53 (m, 1 H) 2.52–2.66 (m, 1 H) 2.92–2.98 (m, 1 H) 3.28–3.34 (m, 1 H) 3.47 (br. s., 1 H) 3.56 (br. s., 1 H) 6.20 (dd, *J* = 5.20, 2.99 Hz, 1 H) 6.58 (t, *J* = 2.30 Hz, 1 H) 7.50–7.55 (m, 2 H) 7.55–7.59 (m, 1 H) 7.77–7.82 (m, 2 H); ^13^C NMR (126 MHz, CDCl_3_) δ ppm 24.35 (d, *J* = 65.39 Hz) 36.21 (d, *J* = 5.45 Hz) 40.87 (d, *J* = 72.66 Hz) 46.15 (d, *J* = 2.72 Hz) 48.14 (d, *J* = 2.73 Hz) 49.62 (d, *J* = 11.81 Hz) 54.78 (d, *J* = 1.82 Hz) 128.98 (d, *J* = 11.81 Hz) 130.25 (d, *J* = 9.08 Hz) 132.16 (d, *J* = 2.73 Hz) 133.37 (d, *J* = 97.18 Hz) 135.84 (s) 137.08 (d, *J* = 3.63 Hz) 208.60 (d, *J* = 10.90 Hz); ^31^P NMR (202 MHz, CDCl_3_) δ ppm 31.32 (s); Elemental Anal. Calcd for C_16_H_17_O_2_P: C, 70.58; H, 6.29; Found: C, 68.76; H, 6.35; HRMS (ESI): *m*/*z* = 273.1054 [C_16_H_17_O_2_P+H]^+^, *m*/*z* (teor.) = 273.1039, diff. = 5.49 ppm.

Crystal data for (*rac*)-*endo*-**17**: C_16_H_17_O_2_P, Mw = 272.26, crystal system monoclinic, space group *P*2_1_/*c*, unit cell dimensions a = 19.0112(6) Å, b = 6.2413(2) Å, c = 11.7247(4) Å, β = 104.256(3)°, V = 1348.35(8) Å^3^, Z = 4. Density (calc) 1.341 g/cm^3^, absorption coeff. 1.761 mm^−1^, F(000) = 576. Collected/independent reflections 5086/2607 [R(int) = 0.0237], data/parameters 2607/172, goodness-of-fit on F^2^ 1.074, final R indices [I > 2σ(I)] R1 = 0.0397, wR2 = 0.1049. CCDC No. 2300713.

(*rac*)*-exo*-**17**; colorless crystals, mp = 178.5–180.1 °C; R_f_ = 0.16 (CHCl_3_/THF = 10:1); ^1^H NMR (500 MHz, CDCl_3_) δ ppm 1.43 (d, *J* = 9.77 Hz, 1 H) 1.72 (d, *J* = 9.14 Hz, 1 H) 2.17 (d, *J* = 10.40 Hz, 1 H) 2.22–2.31 (m, 1 H) 2.31–2.40 (m, 1 H) 2.51 (t, *J* = 8.67 Hz, 1 H) 2.80–2.93 (m, 1 H) 2.97–3.08 (m, 1 H) 3.21 (br. s, 1 H) 3.60 (br. s., 1 H) 6.16–6.20 (m, 1 H) 6.20–6.24 (m, 1 H) 7.54–7.60 (m, 2 H) 7.61–7.66 (m, 1 H) 7.82–7.88 (m, 2 H); ^13^C NMR (126 MHz, CDCl_3_) δ ppm 24.02 (d, *J* = 66.30 Hz) 34.63 (d, *J* = 6.36 Hz) 40.76 (d, *J* = 62.67 Hz) 43.07 (d, *J* = 2.72 Hz) 44.35 (s) 46.80 (s) 52.28 (s) 128.99 (d, *J* = 11.81 Hz) 130.59 (d, *J* = 9.08 Hz) 131.79 (d, *J* = 98.09 Hz) 132.36 (d, *J* = 2.72 Hz) 136.62 (s) 138.50 (d, *J* = 10.90 Hz) 208.32 (d, *J* = 18.17 Hz); ^31^P NMR (202 MHz, CDCl_3_) δ ppm 30.91 (s); Elemental Anal. Calcd for C_16_H_17_O_2_P: C, 70.58; H, 6.29; Found: C, 70.03; H, 6.38; HRMS (ESI): *m*/*z* = 273.1028 [C_16_H_17_O_2_P+H]^+^, *m*/*z* (teor.) = 273.1039, diff. = 4.03 ppm.

Crystal data for (*rac*)-*exo*-**17**: C_16_H_17_O_2_P, Mw = 272.26, crystal system triclinic, space group *P*-1, unit cell dimensions a = 10.8657(4) Å, b = 11.5017(4) Å, c = 13.3826(5) Å, α = 68.854(3)°, β = 66.755(4)°, γ = 88.759(3)°, V = 1418.95(10) Å^3^, Z = 4. Density (calc) 1.274 g/cm^3^, absorption coeff. 1.674 mm^−1^, F(000) = 576. Collected/independent reflections 9768/5462 [R(int) = 0.0208], data/parameters 5462/343, goodness-of-fit on F^2^ 1.026, final R indices [I > 2 σ(I)] R1 = 0.0528, wR2 = 0.1440. CCDC No. 2300712.

#### 3.3.8. Synthesis of *rel*-(*S*_P_,1*S*,2*R*,7*S*,8*R*)-3-Phenyl-3-phosphatricyclo[6.2.2.0^2,7^]dodeca-9-en-6-one 3-Oxide ((*rac*)-*endo*-**18**)

Cycloadduct **18** was obtained as a mixture of isomers *exo*-**18** and *endo*-**18** according to the general procedure, using *rac*-**1** (150 mg, 0.75 mmol), 1,3-cyclohexadiene (240 mg, 3 mmol) and BHT (0.05% mol). The reaction was carried out at 120 °C for 3 h under microwave irradiation. The only *endo*-**18** isomer was separated and characterized. Conversion and isomer ratios were determined by ^31^P NMR as described in Figure 5. The crude product was purified by silica gel chromatography (CHCl_3_/THF = 10.5:0.5 to 8:1) followed by crystallization (acetone/hexane = 2:4) to afford (*rac*)-*endo*-**18** (195 mg, 91%) as a colorless crystals, mp = 186.9–188.7 °C; R_f_ = 0.13 (CHCl_3_/THF = 10:1); ^1^H NMR (500 MHz, CDCl_3_) δ ppm 1.27–1.37 (m, 1 H) 1.37–1.46 (m, 1 H) 1.52–1.64 (m, 2 H) 2.17–2.26 (m, 2 H) 2.52–2.66 (m, 1 H) 2.69–2.86 (m, 2 H) 3.01–3.09 (m, 2 H) 3.26 (d, *J* = 1.89 Hz, 1 H) 6.36 (t, *J* = 7.25 Hz, 1 H) 6.53 (t, *J* = 7.25 Hz, 1 H) 7.50–7.56 (m, 2 H) 7.56–7.60 (m, 1 H) 7.79 (s, 2 H); ^13^C NMR (126 MHz, CDCl_3_) δ ppm 22.67 (d, *J* = 63.58 Hz) 23.36 (s) 27.18 (d, *J* = 11.81 Hz) 29.32 (d, *J* = 3.63 Hz) 33.10 (s) 34.50 (d, *J* = 6.36 Hz) 41.71 (d, *J* = 61.76 Hz) 54.75 (d, *J* = 3.63 Hz) 128.96 (d, *J* = 10.90 Hz) 130.43 (d, *J* = 9.08 Hz) 132.15 (d, *J* = 96.40 Hz) 132.25 (d, *J* = 2.80 Hz) 133.13 (s) 134.26 (s) 208.08 (d, *J* = 18.17 Hz); ^31^P NMR (202 MHz, CDCl_3_) δ ppm 29.18 (s); Elemental Anal. Calcd for C_15_H_21_OP: C, 71.32; H, 6.69 Found: C, 70.45; H, 6.83.

Crystal data for (*rac*)-*endo*-**18** (racemic twin): C_17_H_19_O_2_P, Mw = 286.29, crystal system orthorhombic, space group *P*2_1_2_1_2_1_, unit cell dimensions a = 7.5111(1) Å, b = 11.0155(3) Å, c = 18.4543(4) Å, V = 1526.88(6) Å^3^, Z = 4. Density (calc) 1.245 g/cm^3^, absorption coeff. 1.579 mm^−1^, F(000) = 608. Collected/independent reflections 6129/2963 [R(int) = 0.0156], data/parameters 2963/182, goodness-of-fit on F^2^ 1.077, final R indices [I>2σ I)] R1 = 0.0504, wR2 = 0.1452. CCDC No. 2300714.

#### 3.3.9. Synthesis of (*R*_P_,1*S*,6*R*)-2-Phenyl-2-phosphabicyclo[4.4.0]dec-8-en-5-one 2-Oxide ((*R*_P_)-*cis*-**8**) and (*R*_P_,1*S*,6*S*)-2-Phenyl-2-phosphabicyclo[4.4.0]dec-8-en-5-one 2-Oxide ((*R*_P_)-*trans*-**8**): General Procedure

Enantiopure cycloadduct **8** was obtained as a mixture of *cis* and *trans* isomers according to the general procedure, using (*R*_P_)-1-phenylphosphin-2-en-4-one 1-oxide ((*R*_P_)-**2**)) (800 mg, 4 mmol) and butadiene (approximately 2.5 mL, 9 mmol). To prevent polymerization the 0.05% mol of BHT was added. The reaction was carried out at 110 °C for 6 h under microwave irradiation. Both fractions of each isomer were separated and characterized. Conversion and isomeric ratios were determined by ^31^P NMR as described in Figure 8. The crude isomers were purified by silica gel chromatography (CHCl_3_/THF = 10.5:0.5 to 8:1) to afford (*R*_P_)-*cis*-**8** (551 mg) as a colorless oil and (*R*_P_)-*trans*-**8** (434 mg) as colorless crystals (total yield: 97.5%).

(*R*_P_,1*S*,6*R*)-2-Phenyl-2-phosphabicyclo[4.4.0]dec-8-en-5-one 2-oxide ((*R*_P_)-*cis*-**8**); colorless crystals, mp = 164.2–166.6 °C; [α]_D_ = +5.03 (c 0.99, CHCl_3_); R_f_ = 0.19 (CHCl_3_/THF = 10:1);^1^H NMR (500 MHz, CDCl_3_) δ ppm: 2.1 (dd, *J* = 17.5, 1.4 Hz, 1 H), 2.20–2.30 (m, 1 H) 2.44–2.65 (m, 3 H) 2.65–2.77 (m, 2 H) 2.85 (spt, *J* = 5.50 Hz, 1 H) 2.95–3.04 (m, 2 H) 5.70–5.80 (m, 2 H) 7.55–7.60 (m, 2 H) 7.60–7.65 (m, 1 H) 7.84–7.89 (m, 2 H); ^13^C NMR (126 MHz, CDCl_3_) δ ppm: 22.41 (d, *J* = 2.72 Hz) 24.38 (s) 24.86 (d, *J* = 9.08 Hz) 34.69 (s) 35.20 (d, *J* = 4.54 Hz) 45.60 (d, *J* = 2.72 Hz) 123.99 (d, *J* = 8.17 Hz) 125.24 (s) 129.27 (d, *J* = 11.81 Hz) 130.00 (d, *J* = 9.08 Hz) 131.49 (d, *J* = 96.28 Hz) 132.54 (d, *J* = 2.73 Hz) 207.68 (d, *J* = 6.36 Hz); ^31^P NMR (202 MHz, CDCl_3_) δ ppm: 32.34 (s); Elemental Anal. Calcd for C_15_H_17_O_2_P*H_2_O: C, 64.72; H, 6.88 Found: C, 64.79; H, 6.83.

Crystal data for (*R_P_*)-*cis*-**8** monohydrate: C_15_H_17_O_2_P H_2_O, Mw = 277.26, crystal system orthorhombic, space group *P*2_1_2_1_2_1_, unit cell dimensions a = 8.7328(4) Å, b = 9.5504(4) Å, c = 16.5192(6) Å, V = 1377.73(10) Å^3^, Z = 4. Density (calc) 1.337 g/cm^3^, absorption coeff. 1.786 mm^−1^, F(000) = 588. Collected/independent reflections 5625/2407 [R(int) = 0.0182], data/restraints/parameters 2407/3/180, goodness-of-fit on F^2^ 1.061, final R indices [I > 2 σ(I)] R1 = 0.0257, wR2 = 0.0700. Absolute structure parameter 0.018(9). CCDC No. 2300707.

(*R*_P_,1*S*,6*S*)-2-Phenyl-2-phosphabicyclo[4.4.0]dec-8-en-5-one 2-oxide ((*R*_P_)-*trans*-**8**); colorless crystals, mp = 153.1–157.1 °C; [α]_D_ = +18.25 (c 0.93, CHCl_3_); R_f_ = 0.25 (CHCl_3_/THF = 10:1); ^1^H NMR (500 MHz, CDCl_3_) δ ppm: 1.77–1.86 (m, 1 H) 2.25–2.33 (m, 3 H) 2.38–2.48 (m, 2 H) 2.62–2.73 (m, 1 H) 2.82 (ddt, *J* = 28.50, 13.56, 4.40, 4.40 Hz, 1 H) 3.18–3.26 (m, 1 H) 3.32–3.41 (m, 1 H) 5.59–5.64 (m, 1 H) 5.70–5.75 (m, 1 H) 7.52–7.57 (m, 2 H) 7.58–7.62 (m, 1 H) 7.74–7.80 (m, 2 H); ^13^C NMR (126 MHz, CDCl_3_) δ ppm: 22.95 (d, *J* = 3.63 Hz) 24.47 (d, *J* = 8.17 Hz) 28.01 (d, *J* = 63.58 Hz) 36.49 (d, *J* = 6.36 Hz) 38.99 (d, *J* = 68.12 Hz) 44.98 (d, *J* = 4.54 Hz) 124.12 (d, *J* = 11.81 Hz) 125.06 (s) 128.98 (d, *J* = 10.90 Hz) 129.67 (d, *J* = 97.18 Hz) 130.61 (d, *J* = 9.99 Hz) 132.46 (d, *J* = 2.73 Hz) 208.63 (d, *J* = 7.27 Hz); ^31^P NMR (202 MHz, CDCl_3_) δ ppm: 30.14 (s); Elemental Anal. Calcd for C_15_H_17_O_2_P: C, 69.22; H, 6.58 Found: C, 68.94; H, 6.55.

Crystal data for (*R*_P_)-*trans*-**8**: C_15_H_17_O_2_P, Mw = 260.25, crystal system monoclinic, space group *P*2_1_, unit cell dimensions a = 8.2771(4) Å, b = 5.4442(3) Å, c = 14.7443(5) Å, b = 100.37(1)°, V = 653.56(6) Å^3^, Z = 2. Density (calc) 1.322 g/cm^3^, absorption coefficient 1.789 mm^−1^, F(000) = 276. Collected/independent reflections 4225/2203 [R(int) = 0.0259], data/restraints/parameters 2203/1/163, goodness-of-fit on F^2^ 1.103, final R indices [I>2 σ(I)] R1 = 0.0407, wR2 = 0.1175. Absolute structure parameter 0.055(18). CCDC No. 2300708.

#### 3.3.10. Synthesis of (*R*_P_,1*S*,6*R*)-2-Phenyl-2-phosphabicyclo[4.4.0]dec-5-one 2-Oxide ((*R*_P_)-*cis*-**19**) and (*R*_P_,1*S*,6*S*)-2-Phenyl-2-phosphabicyclo[4.4.0]dec-5-one 2-Oxide ((*R*_P_)-*trans*-**19**)

Hydrogenation of (*R*_P_)-*cis*-**8** (0.26 g, 1 mmol) was accomplished under 1 atm of hydrogen pressure over 24 h in 5 mL of EtOAc using 20 mg of 10% palladium on charcoal as the catalyst. After filtration of the catalyst through a Celite pad and evaporation of the solvent, the residue was crystallized from acetone/hexane (2:4) to afford white crystals of (*R*_P_,1*S*,6*R*)-2-phenyl-2-phosphabicyclo[4.4.0]dec-5-one 2-oxide ((*R*_P_)-*cis*-**19**) (235 mg, 90%), mp = 177.6–179.1 °C; [α]_D_ = −59.1 (c 1.18, CHCl_3_); R_f_ = 0.22 (CHCl_3_/THF = 10:1); ^1^H NMR (500 MHz, CDCl_3_) δ ppm: 1.34–1.44 (m, 1 H), 1.44–1.52 (m, 2 H), 1.55–1.65 (m, 1 H), 1.69–1.80 (m, 1 H), 1.87–2.00 (m, 1 H), 2.00–2.10 (m, 1 H), 2.29–2.48 (m, 3 H), 2.54 (tt, *J* = 9.30, 4.73 Hz, 1 H), 2.59–2.72 (m, 1 H), 2.77–2.86 (m, 1 H), 3.10 (tdd, *J* = 15.92, 15.92, 10.56, 5.20 Hz, 1 H), 7.51–7.63 (m, 3 H), 7.76–7.88 (m, 2 H); ^13^C NMR (126 MHz, CDCl_3_) δ ppm: 23.4 (s), 24.3 (d, *J* = 7.2 Hz), 24.3 (d, *J* = 3.6 Hz), 25.3 (d, *J* = 64.2 Hz), 26.8 (d, *J* = 5.4 Hz), 35.2 (d, *J* = 5.4 Hz), 38.0 (d, *J* = 64.9 Hz), 50.0 (s), 129.1 (d, *J* = 11.8 Hz), 130.2 (d, *J* = 9.1 Hz), 131.5 (d, *J* = 94.6 Hz), 132.3 (d, *J* = 2.7 Hz), 209.5 (d, *J* = 6.4 Hz); ^31^P NMR (202 MHz, CDCl_3_) δ ppm: 32.0 (s); Elemental Anal. Calcd for C_15_H_19_O_2_P: C, 68.69; H, 7.30 Found: C, 68.62; H, 7.38.

Hydrogenation of (*R*_P_)-*trans*-**8** (0.62 g, 2.4 mmol) was accomplished under 1 atm of hydrogen pressure over 24 h in 5 mL of EtOAc using 70 mg of 10% palladium on charcoal as the catalyst. After filtration of the catalyst through a Celite pad and evaporation of the solvent, the residue was crystallized from acetone/hexane (2:4) to afford beige crystals of (*R*_P_,1*S*,6*S*)-2-phenyl-2-phosphabicyclo[4.4.0]dec-5-one 2-oxide ((*R*_P_)-*trans*-**19**) (440 mg, 71%), mp = 196.0–196.9 °C; [α]_D_ = +4.07 (c 1.18, CHCl_3_); R_f_ = 0.28 (CHCl_3_/THF = 10:1); ^1^H NMR (500 MHz, CDCl_3_) δ ppm: 1.00–1.12 (m, 1 H) 1.19–1.32 (m, 1 H) 1.33–1.42 (m, 1 H) 1.42–1.50 (m, 1 H) 1.66–1.74 (m, 2 H) 1.78 (br. d, *J* = 13.24 Hz, 1H) 1.89–1.96 (m, 1 H) 1.97–2.04 (m, 1 H) 2.19–2.28 (m, 1 H) 2.28–2.39 (m, 1 H) 2.70 (ddt, *J* = 28.00, 13.60, 4.70, 4.70 Hz) 3.01 (tt, *J* = 12.10, 4.30 Hz, 1 H) 3.22 (s, 1 H) 7.47–7.52 (m, 2 H) 7.53–7.58 (m, 1 H) 7.68–7.74 (m, 2 H); ^13^C NMR (126 MHz, CDCl_3_) δ ppm: 23.76 (d, *J* = 4.54 Hz) 24.42 (s) 25.04 (d, *J* = 11.81 Hz) 25.94 (d, *J* = 8.17 Hz) 28.05 (d, *J* = 64.49 Hz) 36.64 (d, *J* = 5.45 Hz) 43.11 (d, *J* = 68.12 Hz) 48.37 (d, *J* = 4.54 Hz) 128.90 (d, *J* = 11.50 Hz) 129.55 (d, *J* = 95.37 Hz) 130.64 (d, *J* = 9.08 Hz) 132.33 (d, *J* = 2.72 Hz) 209.30 (d, *J* = 5.45 Hz); ^31^P NMR (202 MHz, CDCl_3_) δ ppm: 30.90 (s); Elemental Anal. Calcd for C_15_H_19_O_2_P: C, 68.69; H, 7.30 Found: C, 68.65; H, 7.35.

#### 3.3.11. Synthesis of (*R*_P_,1*S*,6*R*)-2-Phenyl-2-phosphabicyclo[4.4.0]decane 2-Oxide ((*R*_P_)-*trans*-**20**)

In a flame-dried, argon-filled Schlenk flask equipped with a stirring bar, (*R*_P_)-*trans*-**19** (0.3 g, 1.15 mmol) was added to a solution of KOH (0.55 g, 9.2 mmol) and hydrazine hydrate (0.28 mL, 4.8 mmol) in 5 mL of ethylene glycol. A reflux condenser fitted to the flask and sealed with an argon-filled balloon. After 3 h, the condenser was removed and glycol was evaporated under reduced pressure. The obtained viscous oil was then purified by column chromatography (CHCl_3_/THF = 10:1) to afford 0.205 g (0.79 mmol, 69%) of product (*R*)-*trans*-**20**.

(*R*_P_,1*S*,6*R*)-2-phenyl-2-phosphabicyclo[4.4.0]decane 2-oxide ((*R*_P_)-*trans*-**20**); colorless crystals, mp = 157.7–158.8 °C; [α]_D_ = +20.09 (c 1.11, CHCl_3_); R_f_ = 0.35 (CHCl_3_/THF = 10:1); ^1^H NMR (500 MHz, CDCl_3_) δ ppm: 1.03–1.18 (m, 2 H) 1.18–1.29 (m, 1 H) 1.31–1.49 (m, 3 H) 1.51–1.62 (m, 1 H) 1.67–1.75 (m, 2 H) 1.76–1.85 (m, 3 H) 1.92–2.10 (m, 3 H) 2.12–2.24 (m, 1 H) 7.45–7.54 (m, 3 H) 7.71–7.78 (m, 2 H); ^13^C NMR (126 MHz, CDCl_3_) δ ppm: 20.75 (d, *J* = 5.45 Hz) 22.93 (d, *J* = 4.54 Hz) 25.81 (s) 26.04 (d, *J* = 12.72 Hz) 28.82 (d, *J* = 64.49 Hz) 34.81 (d, *J* = 9.99 Hz) 35.21 (d, *J* = 4.54 Hz) 37.25 (d, *J* = 4.54 Hz) 43.22 (d, *J* = 66.30 Hz) 128.51 (d, *J* = 10.90 Hz) 130.63 (d, *J* = 9.99 Hz) 131.50 (d, *J* = 2.72 Hz) 132.04 (d, *J* = 91.73 Hz); ^31^P NMR (202 MHz, CDCl_3_) δ ppm: 30.12 (s); Elemental Anal. Calcd for C_15_H_21_OP: C, 72.56; H, 8.52 Found: C, 72.53; H, 8.46.

## 4. Conclusions

In summary, a highly efficient synthesis of new bicyclic and tricyclic phosphadecalone systems was achieved. The key basis for this approach is the high activity of 1-phenylphosphin-2-en-4-one 1-oxide (**2**) used as a dienophile, which undergoes stereoselective Diels–Alder cycloadditions with the acyclic and cyclic dienes. The reaction provides cycloadducts via the *endo* mode and only from the P=O bond side of the molecule, when, at the same time, the second opposite substituent is a phenyl group. The developed procedure is general in nature and uses microwave irradiation to accelerate the process and increase the efficiency of the synthesized cycloadducts. Appropriately selected parameters of syntheses (temperature and time) allow the control of the isomer selectivity of the resulting *cis*- or *trans*-fused cyclization product, which is characterized by a completely different molecular stereochemistry.

Under thermal and acidic conditions, the izomerization of kinetically preferred *cis*-fused phosphadecalones gives the *trans*-fused isomers practically exclusively. In the case of the kinetically preferred *endo* stereoisomer of tricyclic phosphine **17**, it has been shown to convert to the corresponding thermodynamically preferred *exo* stereoisomer via a retro-Diels-Alder reaction.

We further demonstrate this strategy for also accessing the enantiomerically pure P,C,C-chiral phosphadecalenone via cycloaddition with the enantiomerically pure dienophile (*R*)-**2**. The synthetic transformations demonstrated the applicability of the enantiomerically pure phosphadecalenone (*R*)-**4** to other derivatives that can serve as *P*-stereogenic monophosphine ligands in asymmetric catalysis. Research is currently being conducted in our laboratory to achieve this goal.

## Data Availability

Data are contained within the article and Supplementary Materials.

## Supplementary Materials

The following supporting information can be downloaded at https://www.mdpi.com/article/10.3390/molecules30112338/s1, Figures S1–S45: NMR spectra (^1^H 500 MHz, ^13^C NMR 126 MHz, ^31^P NMR 202 MHz); crystallographic data (CCDC Nos 2300707–2300714—these data are provided free of charge by the Cambridge Crystallographic Data Centre Access Structures service www.ccdc.cam.ac.uk/structures/?).
