# Supplementary material for: A Straightforward Approach Towards Phosphadecalones by Microwave-Assisted Diels–Alder Reaction
Source: Molecules. 2025 May 27;30(11):2338. doi: 10.3390/molecules30112338 (PMC12155891; doi:10.3390/molecules30112338)
Supplement: Supplementary file 1 [file molecules-30-02338-s001.zip › molecules-3644922-supplementary.pdf]

molecules

Supporting Information

A Straightforward Approach Towards Phosphadecalones by Microwave-Assisted Diels–Alder Reaction

Elżbieta Łastawiecka[1,*], Anna E. Kozioł [1,*], and K. Michał Pietrusiewicz [1]

[1]Department of Organic Chemistry and Crystal Chemistry, Institute of Chemical Sciences, Faculty of Chemistry, Maria Curie-Sklodowska University, 20-031, Lublin, Poland

***** elzbieta.lastawiecka@mail.umcs.pl ; anna.koziol@mail.umcs.pl

(EL) ORCID 0000-0001-6546-1519; (AEK) ORCID 0000-0001-5339-7505; (KMP) ORCID 0000- 0003-0969-4689

^{1}H NMR, ^{13}C NMR and ^{31}P NMR spectra of novel phosphorus-containing bicyclic and tricyclic cycloadducts derived from 1-phenylphosphin-2-en-4-one 1-oxide

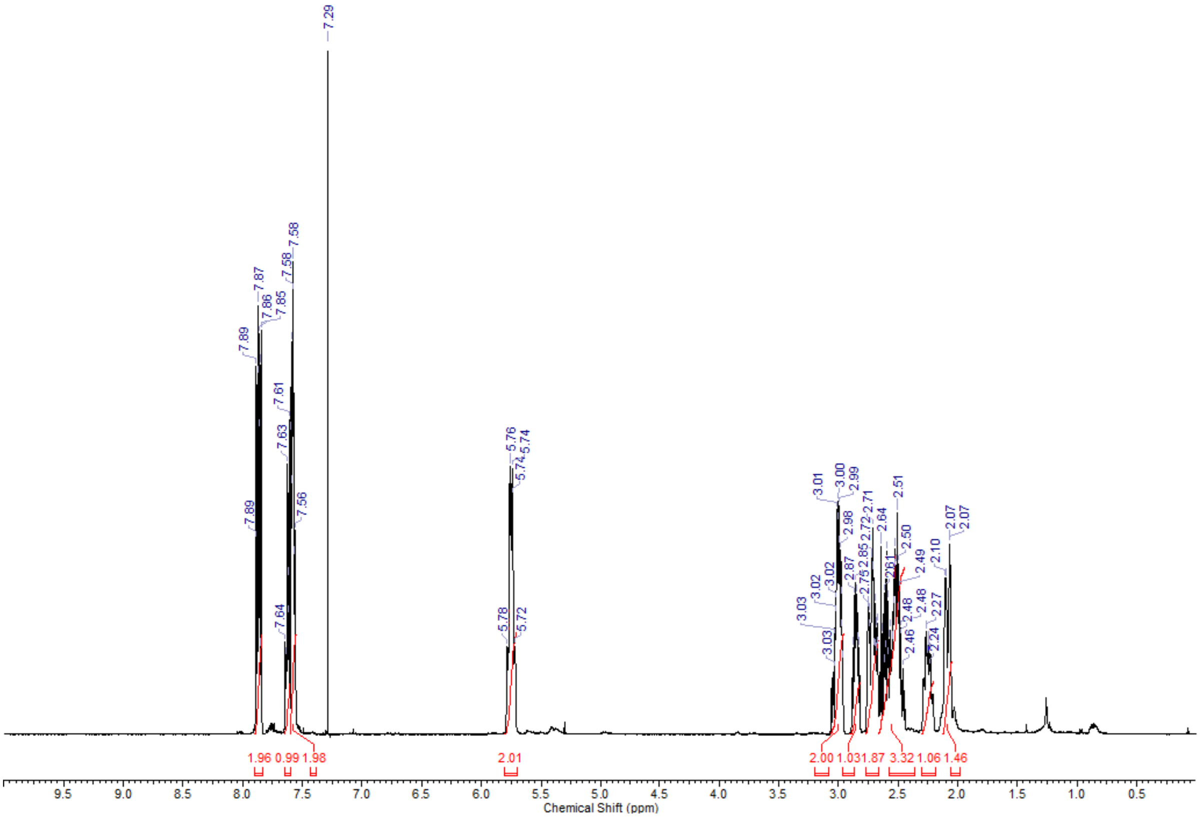

Fig. S1. ^{1}H NMR spectrum of *rel*-(S_P,1*R*,6*S*)-2-phenyl-2-phosphabicyclo[4.4.0]dec-8-en-5-one 2-oxide ((*rac*)-*cis*-**8**) ($CDCl_3$, 500 MHz).

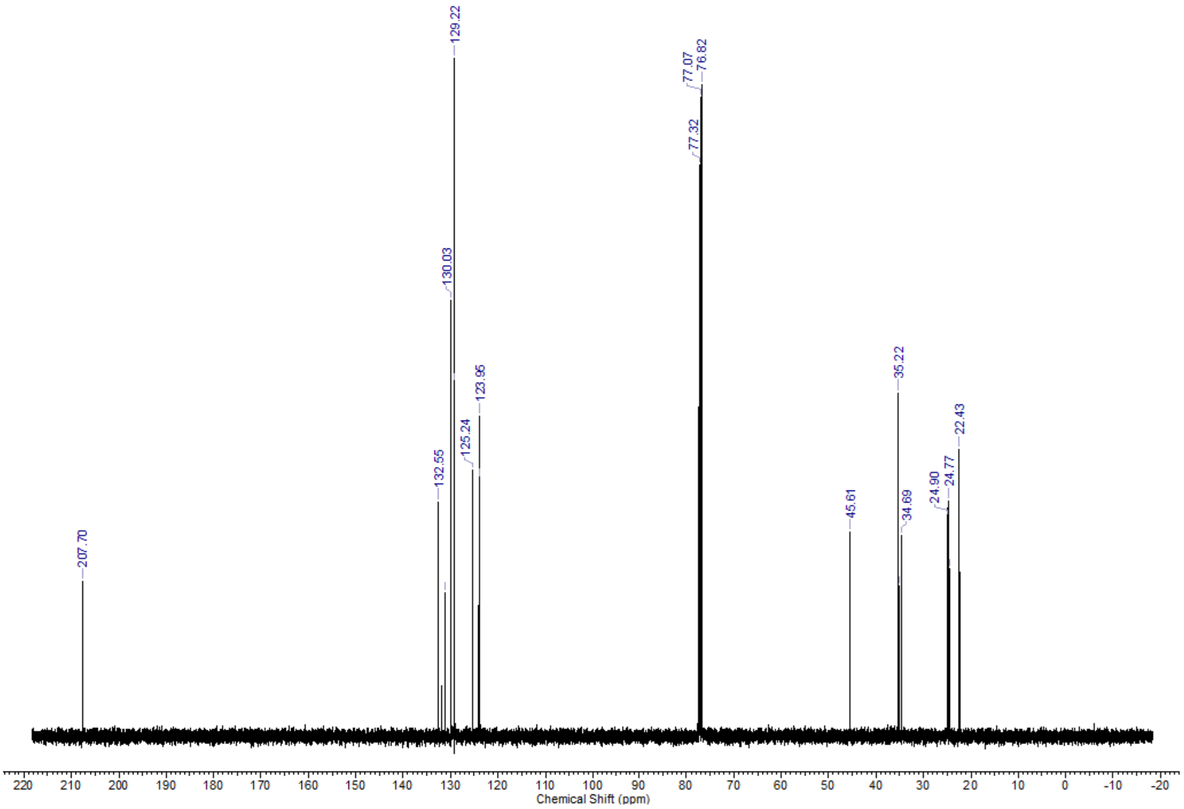

Fig. S2. ^{13}C NMR spectrum of *rel*-(S_P,1*R*,6*S*)-2-phenyl-2-phosphabicyclo[4.4.0]dec-8-en-5-one 2-oxide ((*rac*)-*cis*-**8**) ($CDCl_3$, 126 MHz).

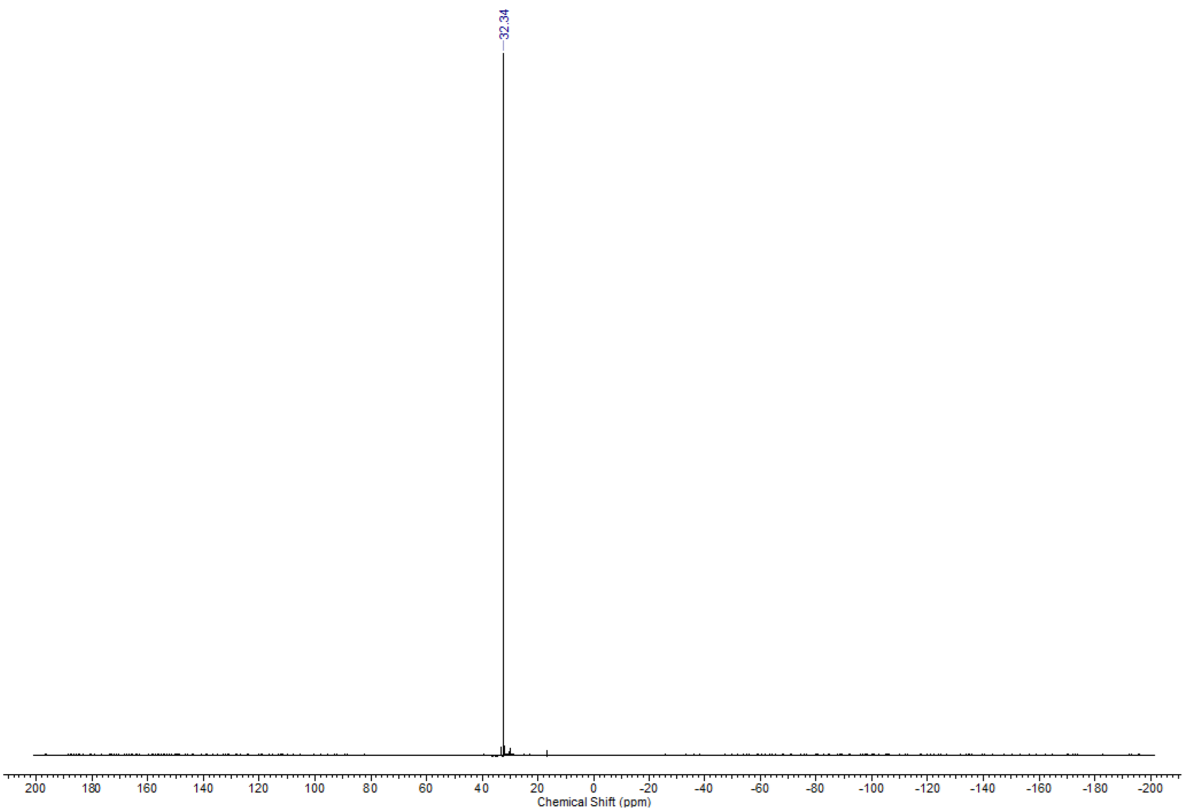

Fig. S3. ^{31}P NMR spectrum of *rel*-(S_P,1*R*,6*S*)-2-phenyl-2-phosphabicyclo[4.4.0]dec-8-en-5-one 2-oxide ((*rac*)-*cis*-**8**) ($CDCl_3$, 202 MHz).

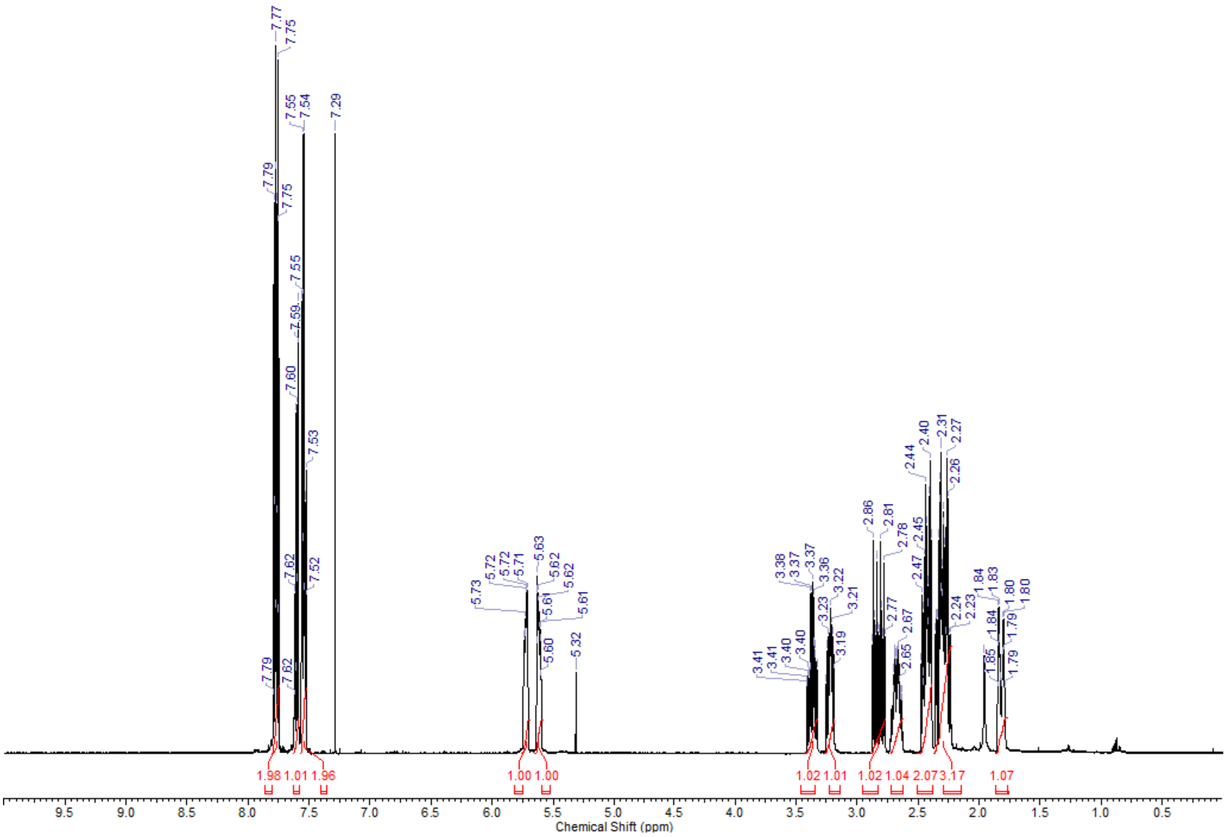

Fig. S4. ^{1}H NMR spectrum of *rel*-(S_P,1*R*,6*R*)-2-phenyl-2-phosphabicyclo[4.4.0]dec-8-en-5-one 2-oxide ((*rac*)-*trans*-**8**) ($CDCl_3$, 500 MHz).

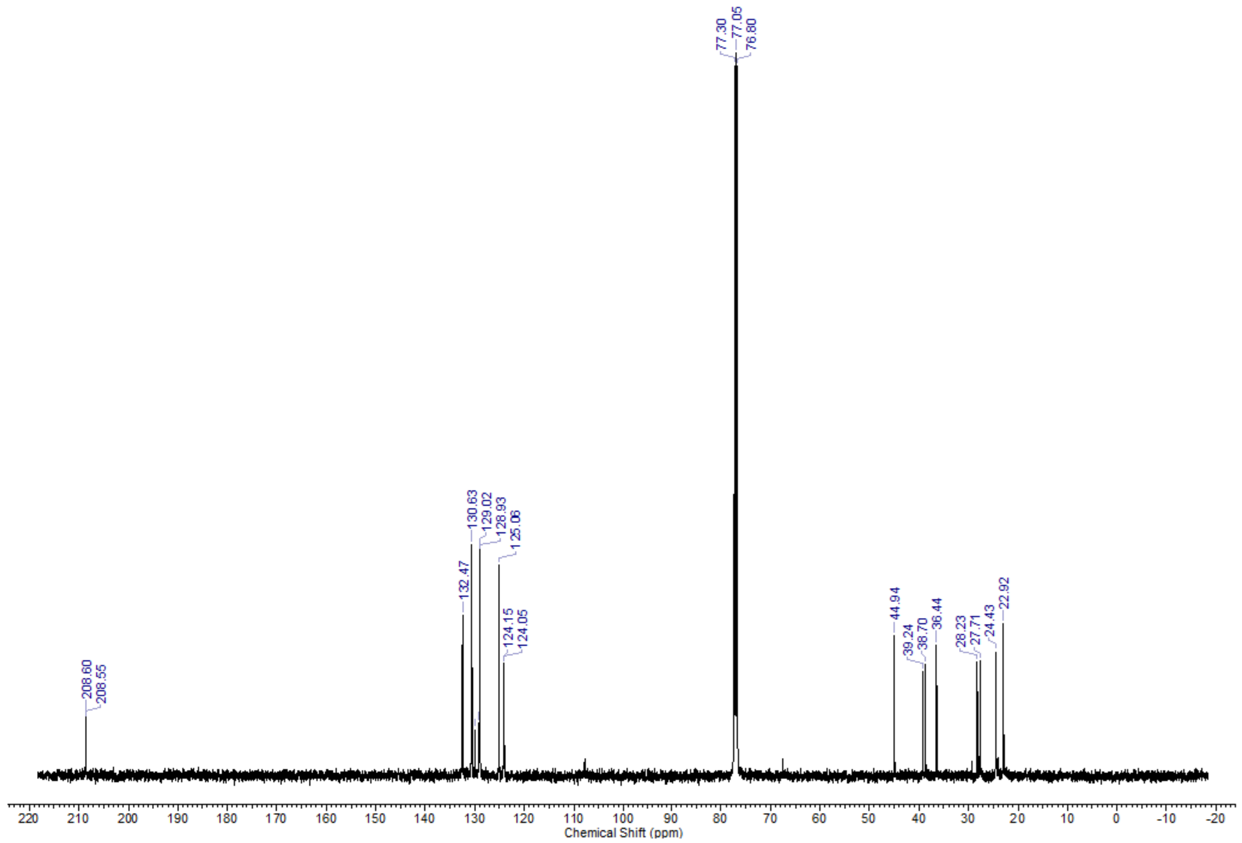

Fig. S5. ^{13}C NMR spectrum of *rel*-(S_{P},1*R*,6*R*)-2-phenyl-2-phosphabicyclo[4.4.0]dec-8-en-5-one 2-oxide ((*rac*)-*trans*-**8**) ($CDCl_3$, 126 MHz).

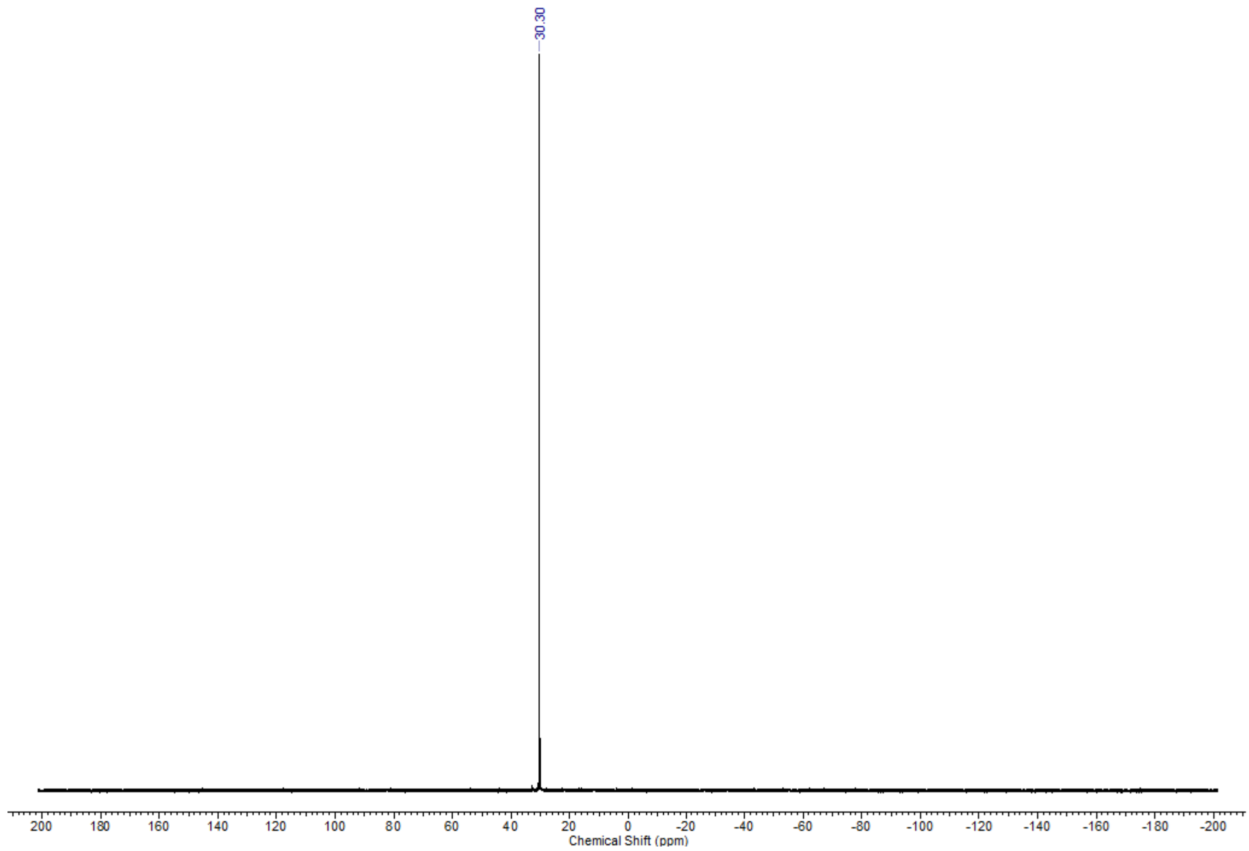

Fig. S6. ^{31}P NMR spectrum of *rel*-(S_{P},1*R*,6*R*)-phenyl-2-phosphabicyclo[4.4.0]dec-8-en-5-one 2-oxide ((*rac*)-*trans*-**8**) ($CDCl_3$, 202 MHz).

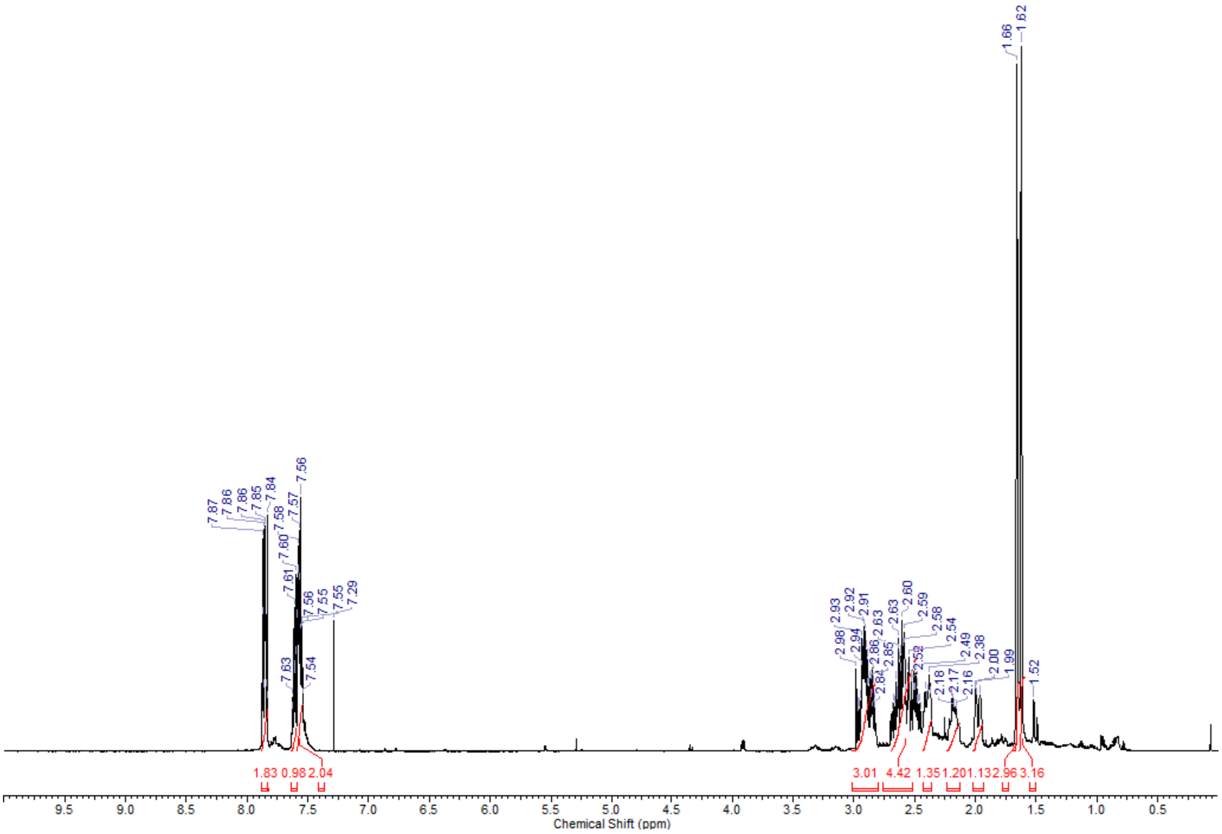

Fig. S7. ^{1}H NMR spectrum of *rel*-(S_P,1*R*,6*S*)-8,9-dimethyl-2-phenyl-2-phosphabicyclo[4.4.0]dec-8-en-5-one 2-oxide ((*rac*)-*cis*-**9**) ($CDCl_3$, 500 MHz).

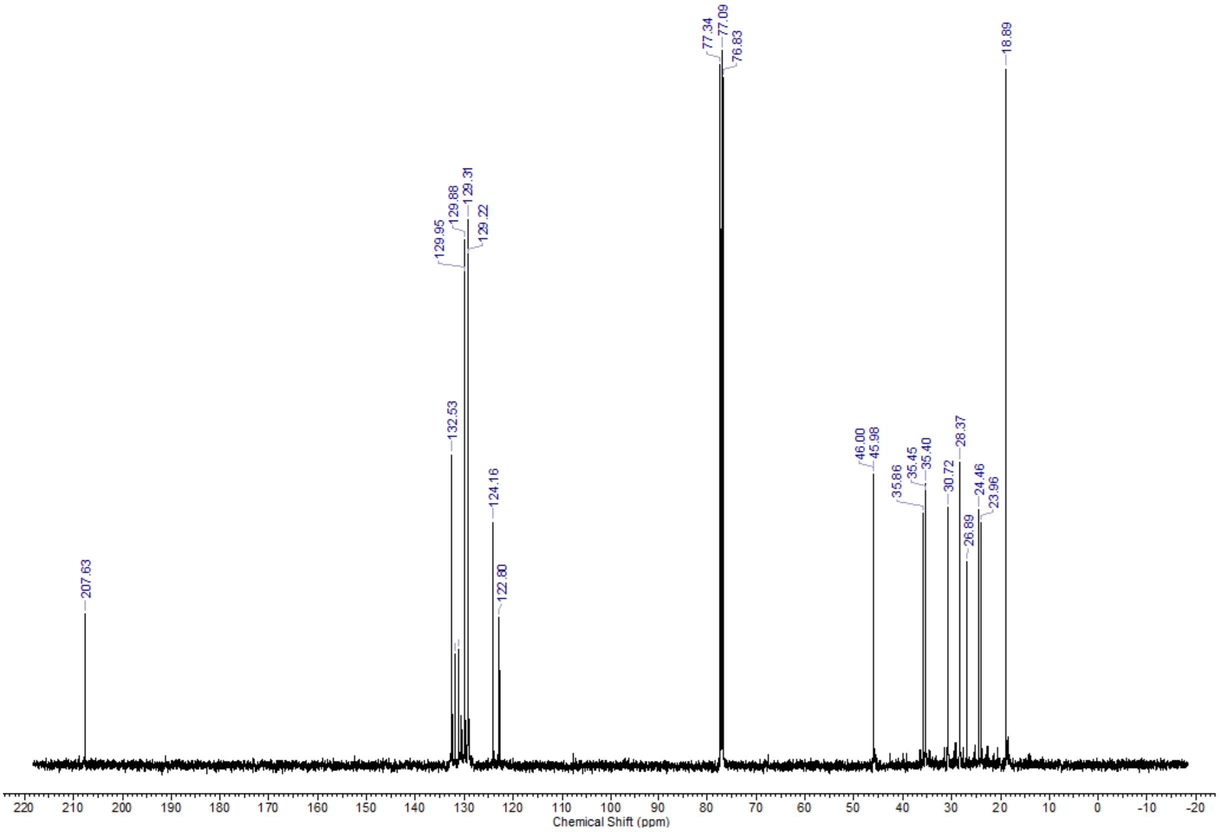

Fig. S8. ^{13}C NMR spectrum of *rel*-(S_P,1*R*,6*S*)-8,9-dimethyl-2-phenyl-2-phosphabicyclo[4.4.0]dec-8-en-5-one 2-oxide ((*rac*)-*cis*-**9**) ($CDCl_3$, 126 MHz).

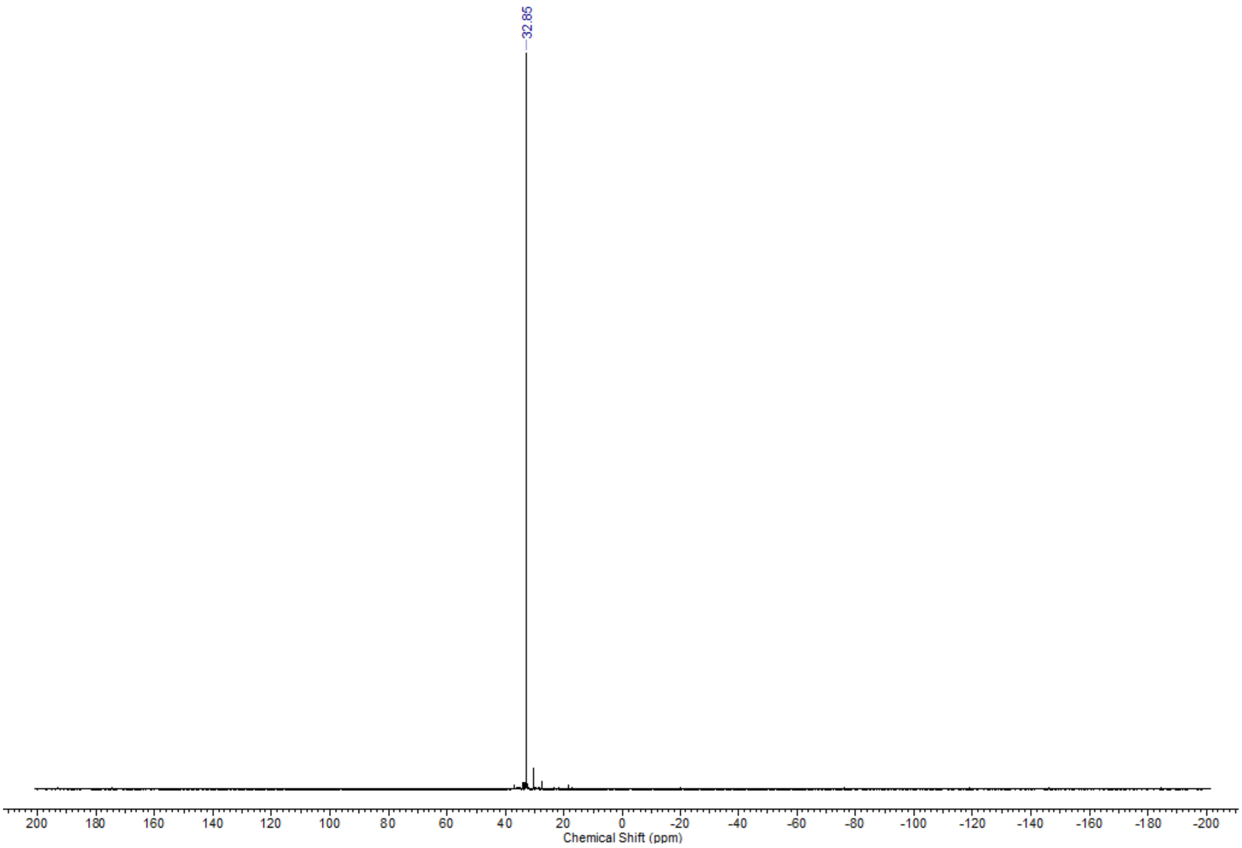

Fig. S9. ^{31}P NMR spectrum of *rel*-(S_P,1*R*,6*S*)-8,9-dimethyl-2-phenyl-2-phosphabicyclo[4.4.0]dec-8-en-5-one 2-oxide ((*rac*)-*cis*-**9**) ($CDCl_3$, 202 MHz).

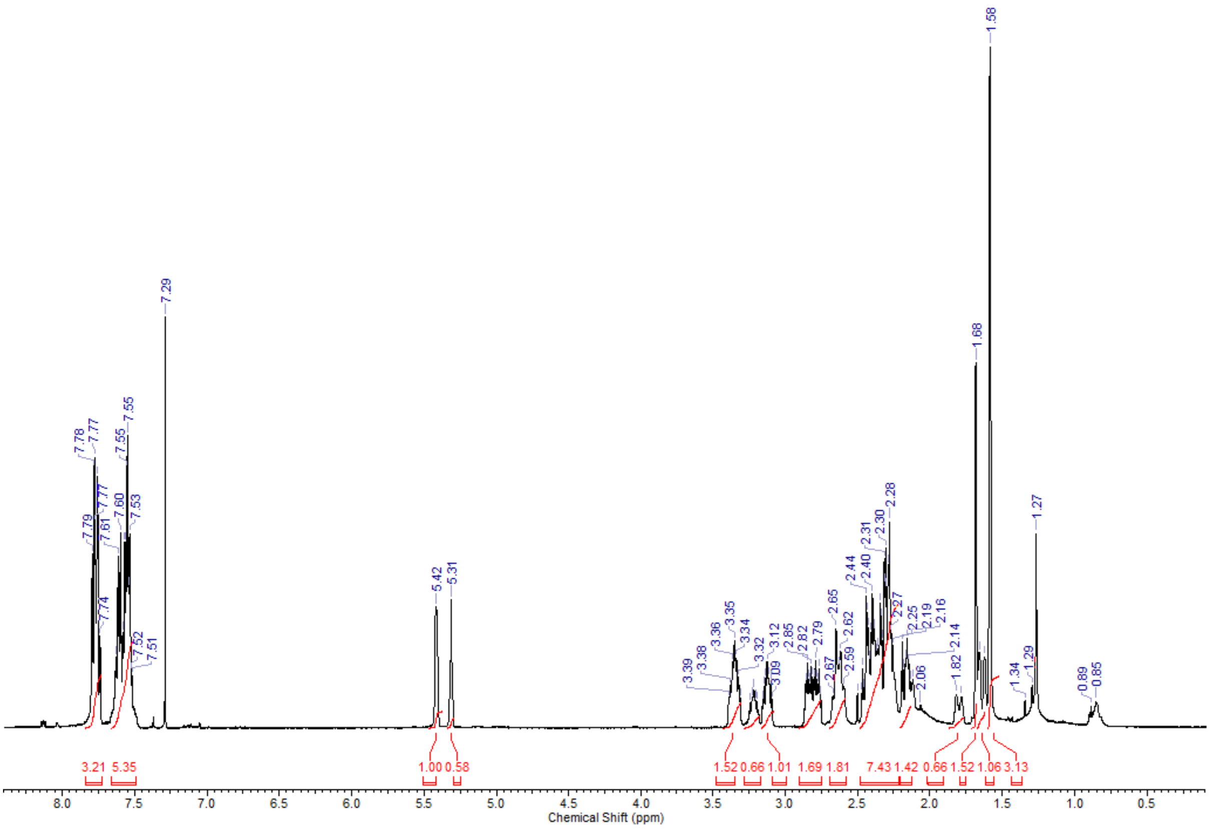

Fig. S10. ^{1}H NMR spectrum of *rel*-(S_P,1*R*,6*S*)-8-methyl-2-phenyl-2-phosphabicyclo[4.4.0]dec-8-en-5-one 2-oxide and *rel*-(S_P,1*R*,6*S*)-9-methyl-2-phenyl-2-phosphabicyclo[4.4.0]dec-8-en-5-one 2-oxide mixture ((*rac*)-*cis*-**10a** /*cis*-**10b** = 1/1.9) ($CDCl_3$, 500 MHz).

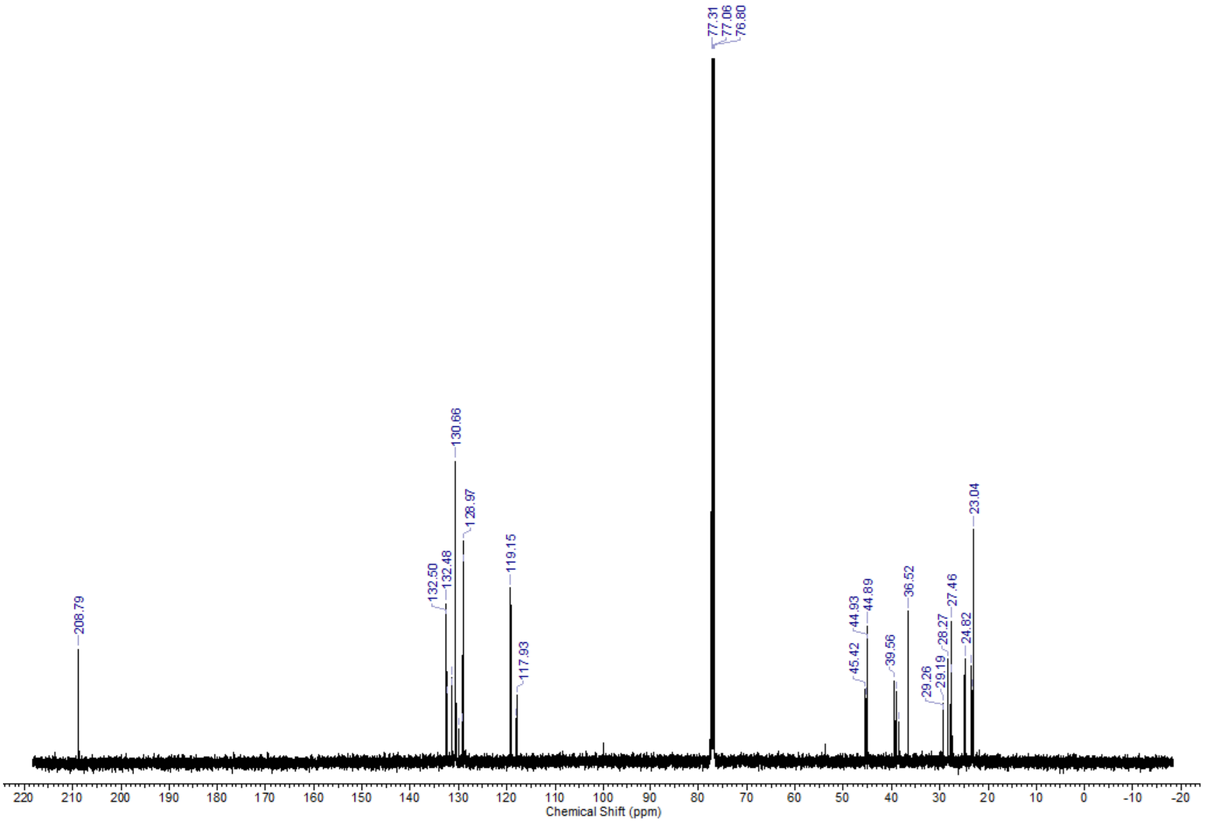

Fig. S11. ^{13}C NMR spectrum of *rel*-(S_P,1*R*,6*S*)-8-methyl-2-phenyl-2-phosphabicyclo[4.4.0]dec-8-en-5-one 2-oxide and *rel*-(S_P,1*R*,6*S*)-9-methyl-2-phenyl-2-phosphabicyclo[4.4.0]dec-8-en-5-one 2-oxide mixture ((*rac*)-*cis*-**10a** /*cis*-**10b** = 1/1.9) ($CDCl_3$, 126 MHz).

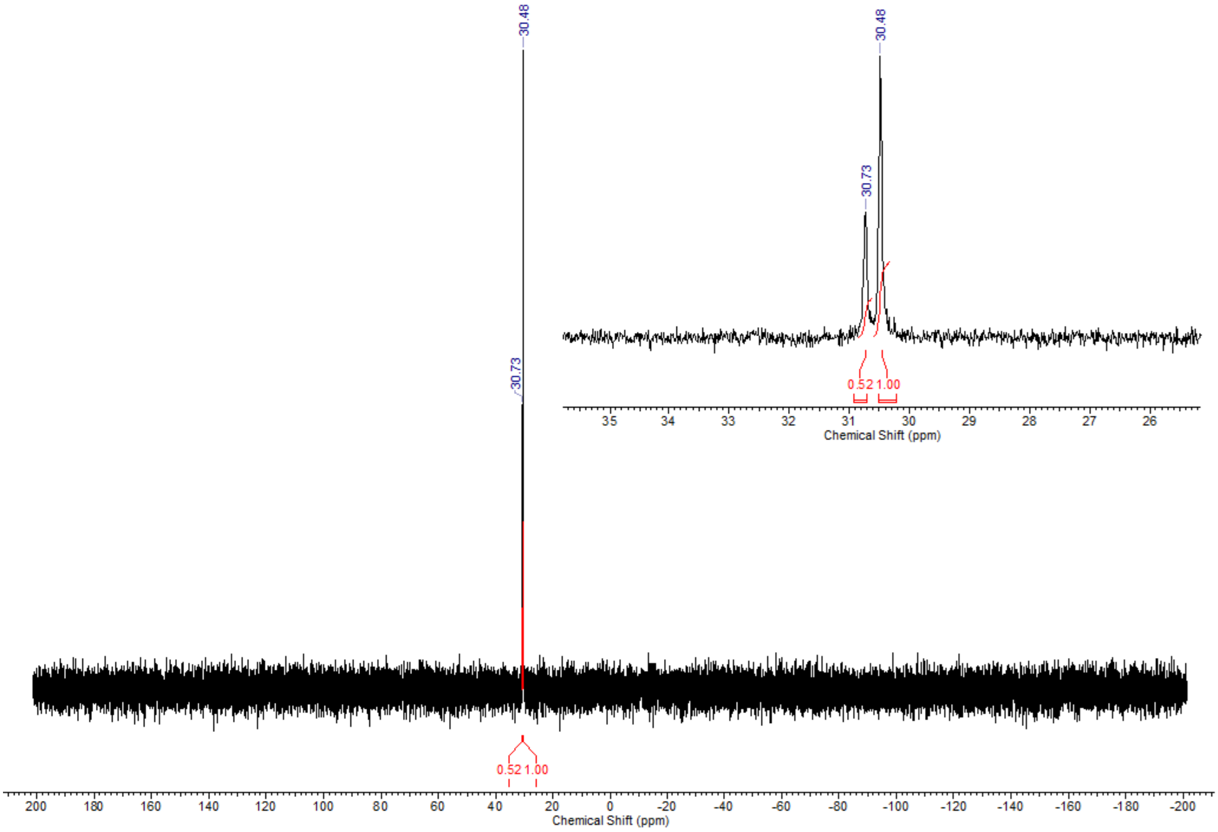

Fig. S12. ^{31}P NMR spectrum of *rel*-(S_P,1*R*,6*S*)-8-methyl-2-phenyl-2-phosphabicyclo[4.4.0]dec-8-en-5-one 2-oxide and *rel*-(S_P,1*R*,6*S*)-9-methyl-2-phenyl-2-phosphabicyclo[4.4.0]dec-8-en-5-one 2-oxide mixture ((*rac*)-*cis*-**10a** /*cis*-**10b** = 1/1.9) ($CDCl_3$, 202 MHz).

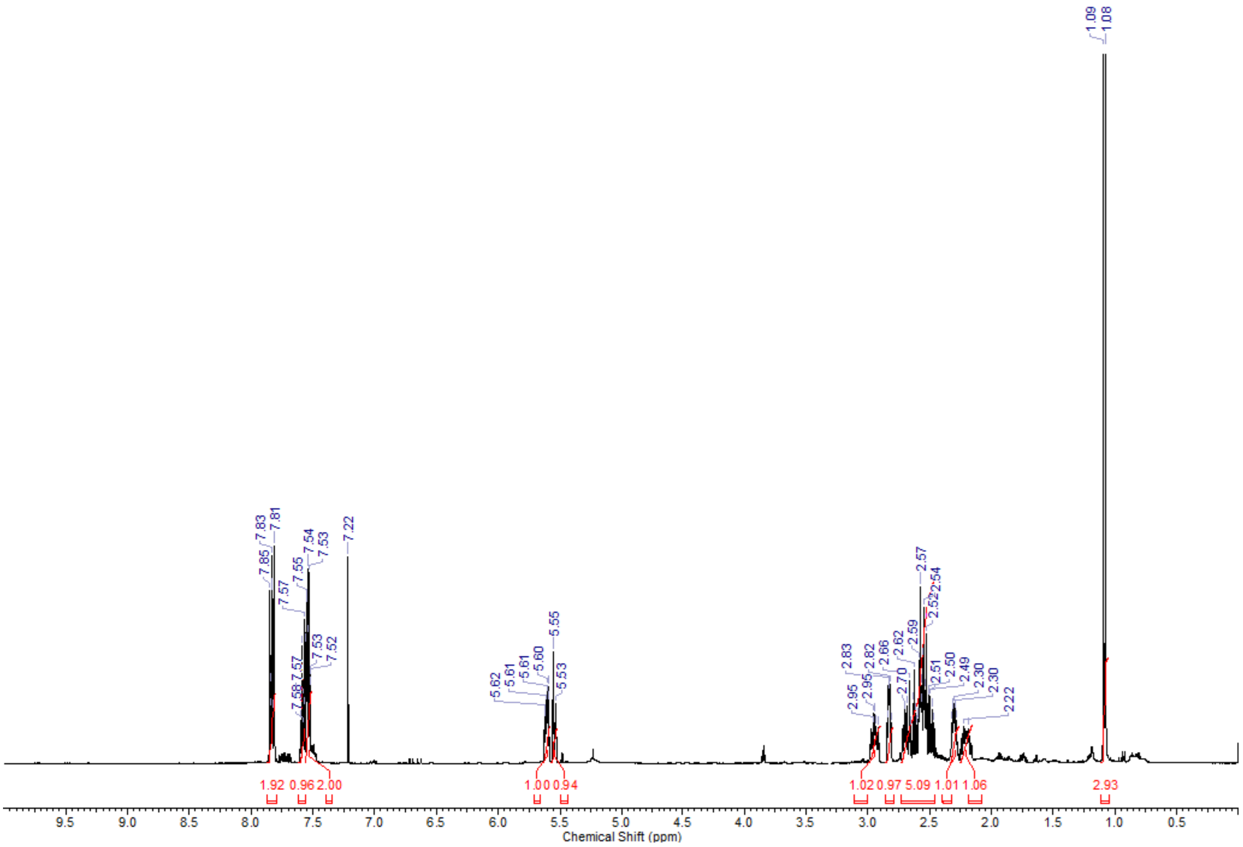

Fig. S13. ^{1}H NMR spectrum of (*rac*)-*cis*-7-methyl-2-phenyl-2-phosphabicyclo[4.4.0]dec-8-en-5-one 2-oxide ((*rac*)-*cis*-**11**) ($CDCl_3$, 500 MHz).

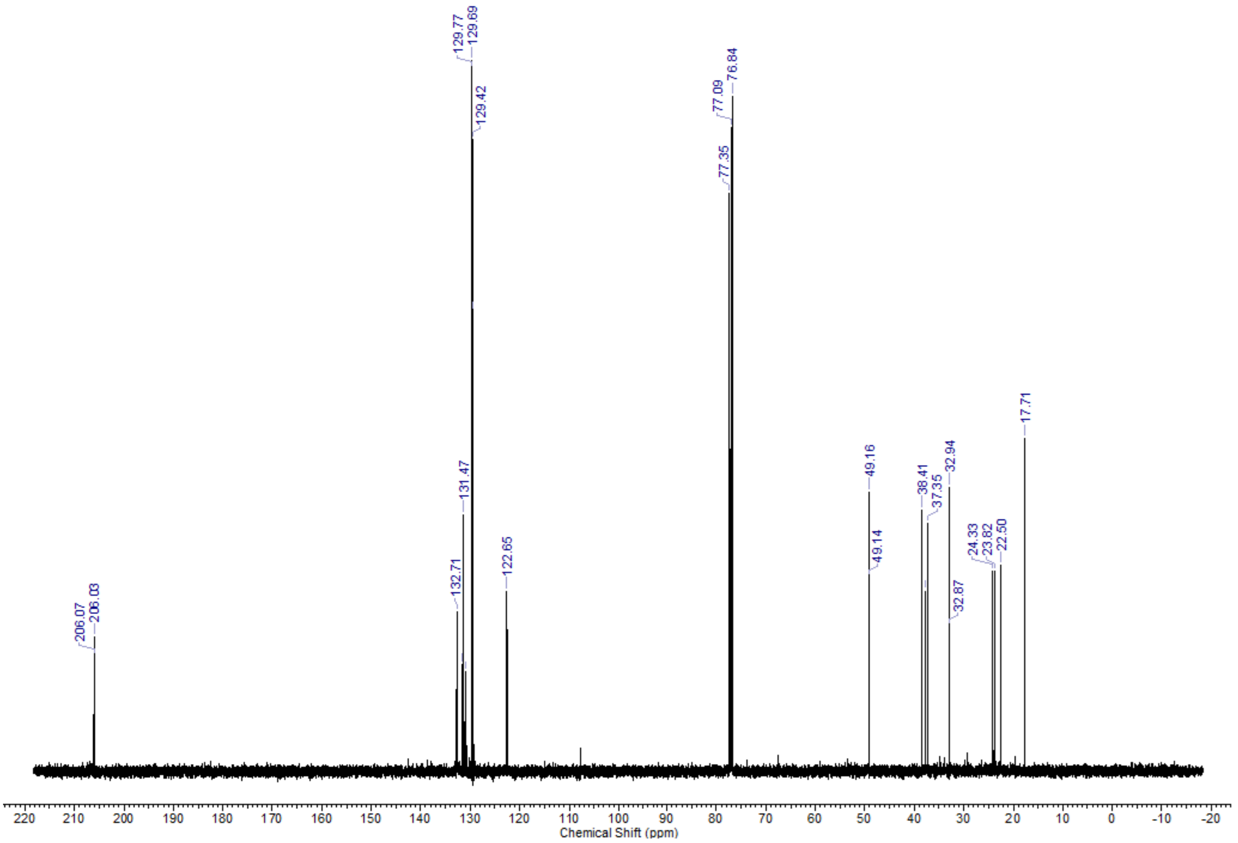

Fig. S14. ^{13}C NMR spectrum of (*rac*)-*cis*-7-methyl-2-phenyl-2-phosphabicyclo[4.4.0]dec-8-en-5-one 2-oxide ((*rac*)-*cis*-**11**) ($CDCl_3$, 126 MHz).

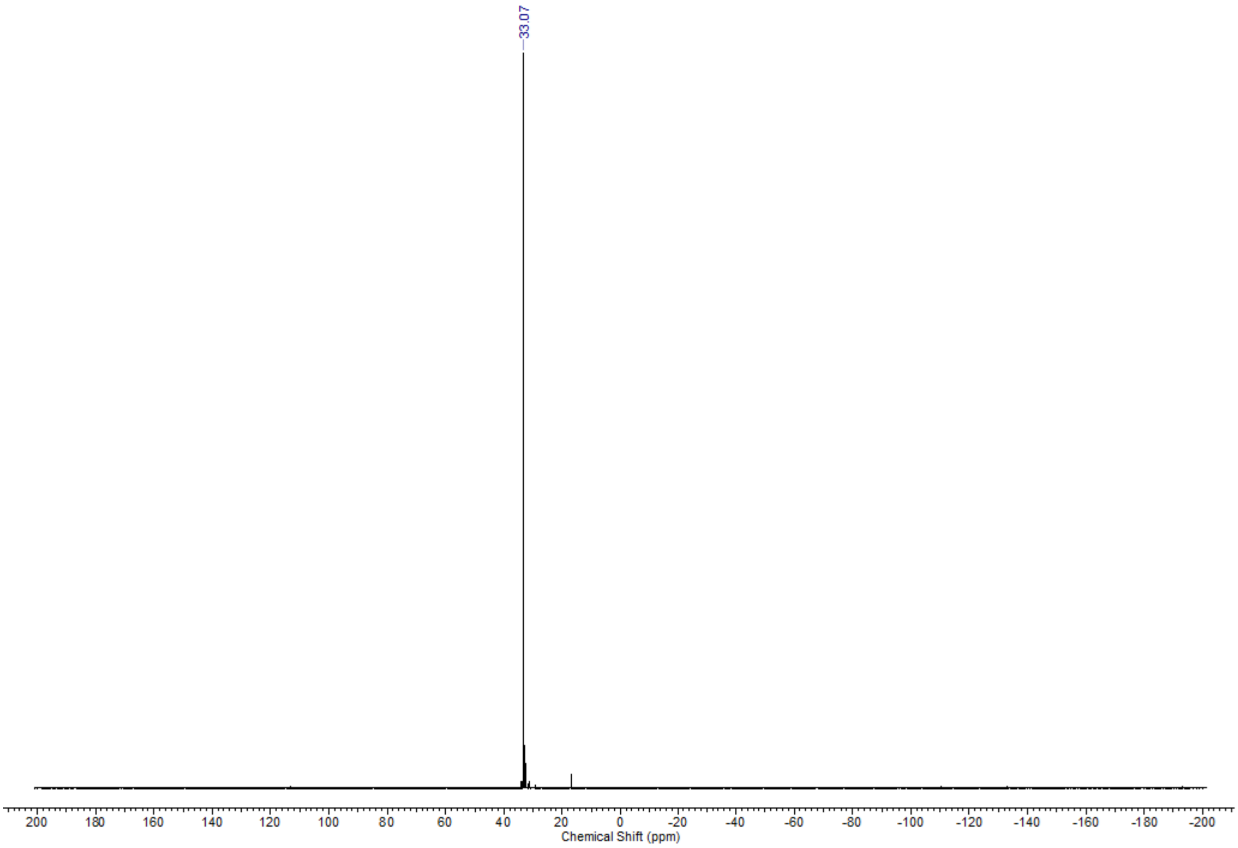

Fig. S15. ^{31}P NMR spectrum of (*rac*)-*cis*-7-methyl-2-phenyl-2-phosphabicyclo[4.4.0]dec-8-en-5-one 2-oxide ((*rac*)-*cis*-**11**) ($CDCl_3$, 202 MHz).

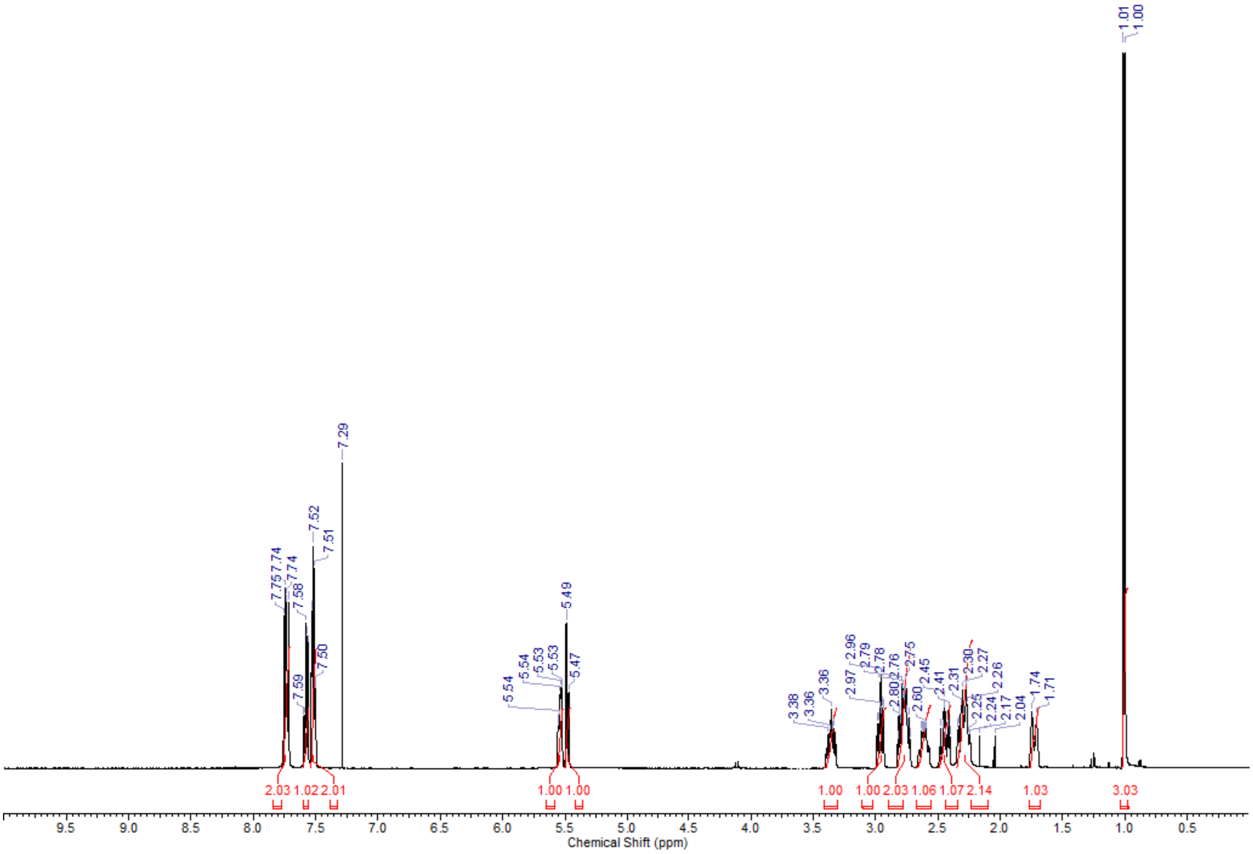

Fig. S16. ^{1}H NMR spectrum of (*rac*)-*trans*-7-methyl-2-phenyl-2-phosphabicyclo[4.4.0]dec-8-en-5-one 2-oxide ((*rac*)-*trans*-**11**) ($CDCl_3$, 500 MHz).

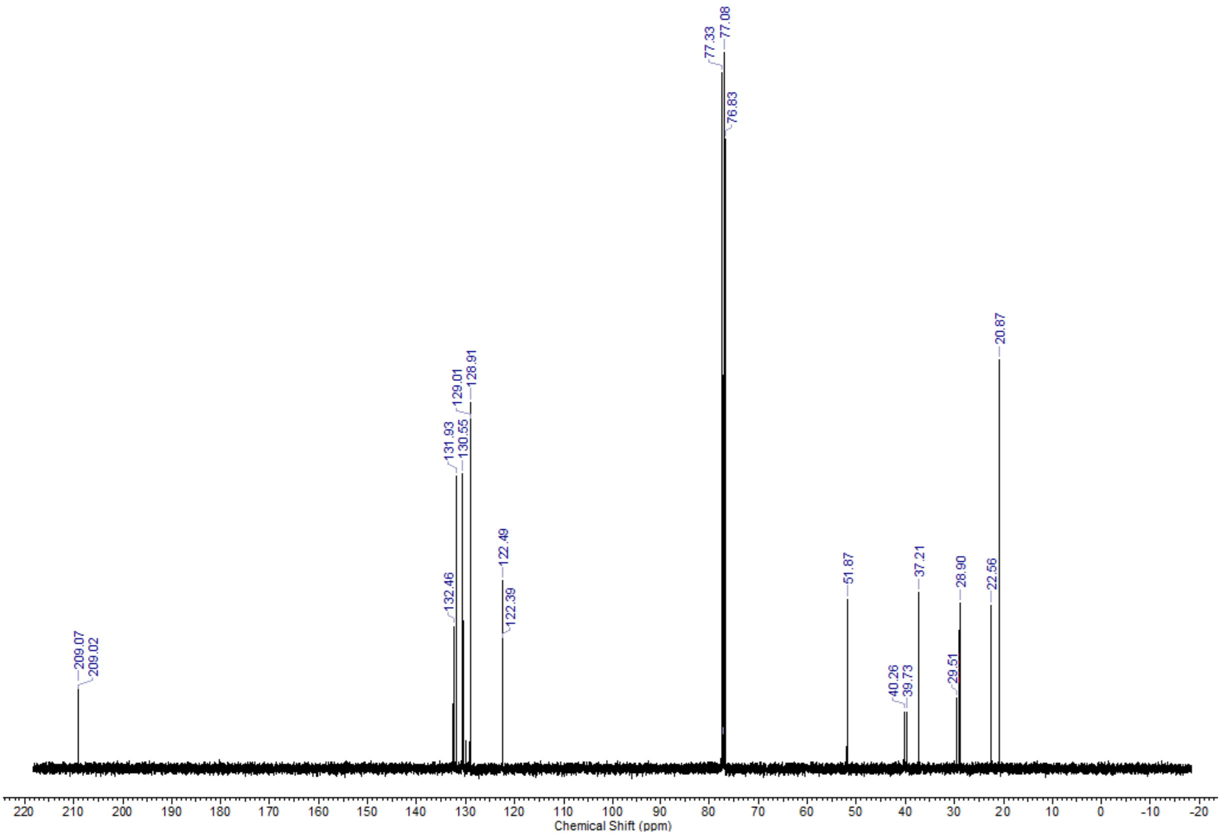

Fig. S17. ^{13}C NMR spectrum of (*rac*)-*trans*-7-methyl-2-phenyl-2-phosphabicyclo[4.4.0]dec-8-en-5-one 2-oxide ((*rac*)-*trans*-**11**) ($CDCl_3$, 126 MHz).

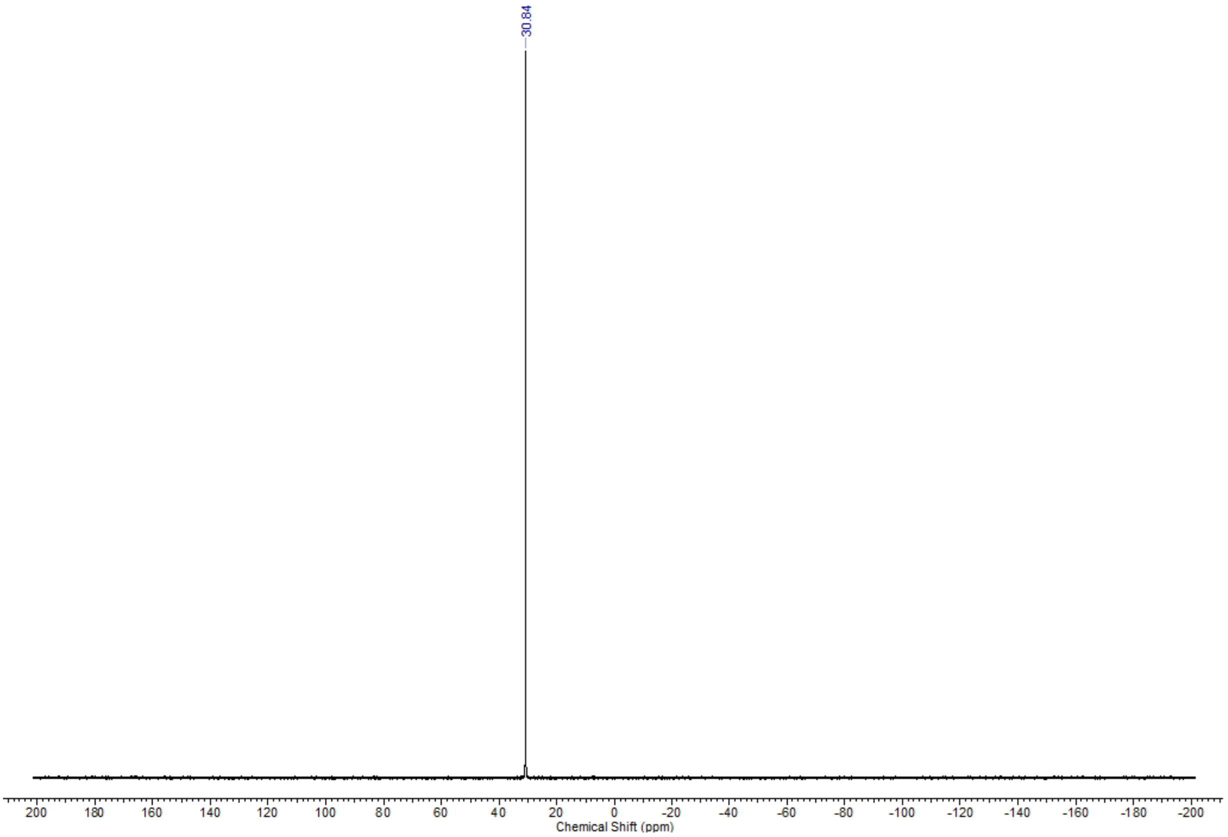

Fig. S18. ^{31}P NMR spectrum of (*rac*)-*trans*-7-methyl-2-phenyl-2-phosphabicyclo[4.4.0]dec-8-en-5-one 2-oxide ((*rac*)-*trans*-**11**) ($CDCl_3$, 202 MHz).

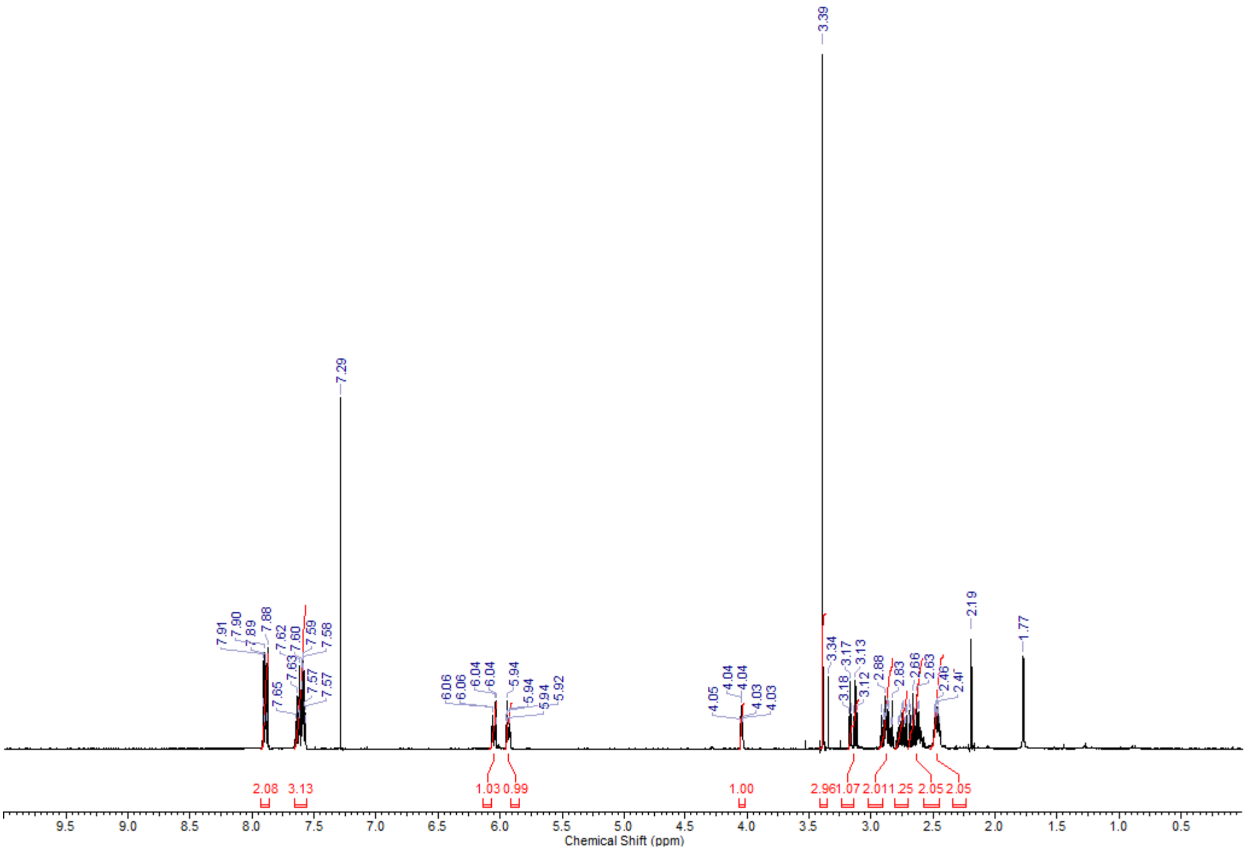

Fig. S19. ^{1}H NMR spectrum of (*rac*)-*cis*-7-methoxy-2-phenyl-2-phosphabicyclo[4.4.0]dec-8-en-5-one 2-oxide ((*rac*)-*cis*-**12**) ($CDCl_3$, 500 MHz).

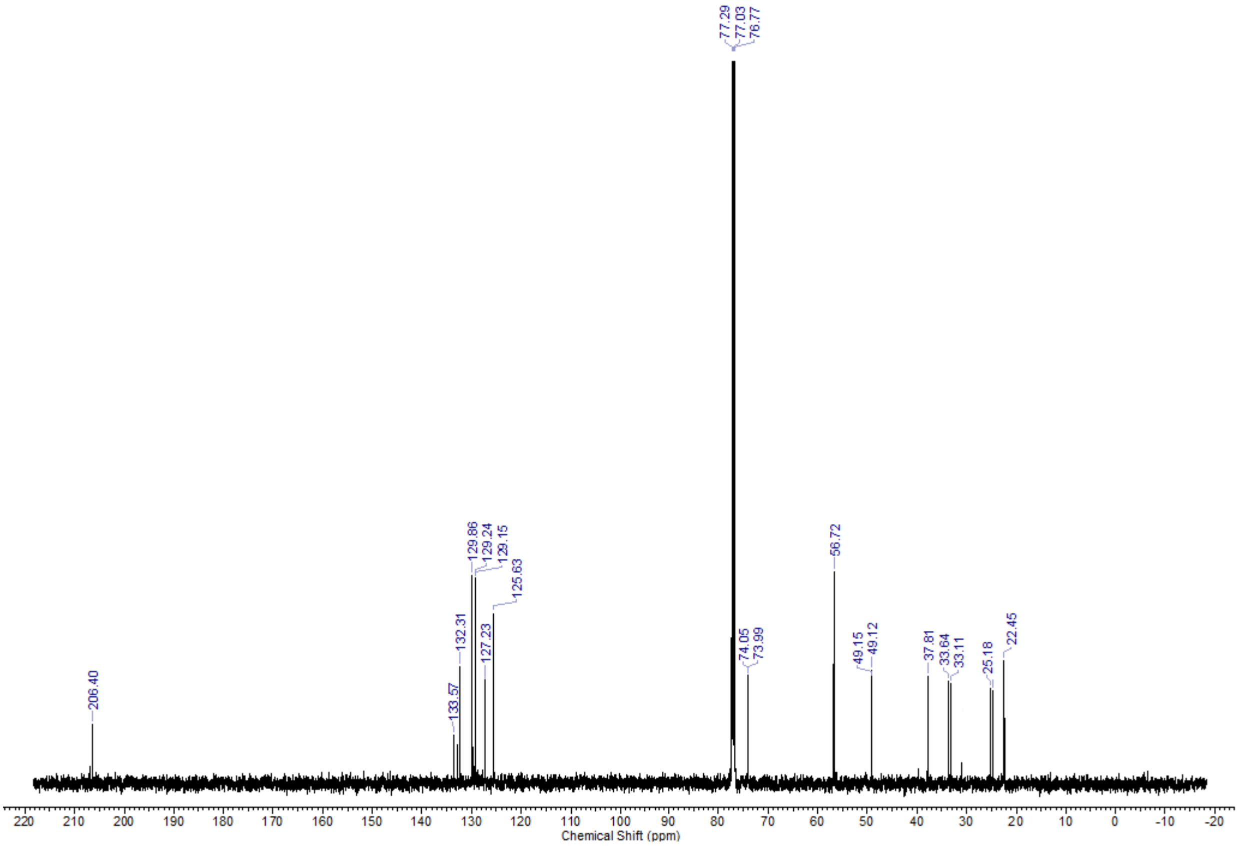

Fig. S20. ^{13}C NMR spectrum of (*rac*)-*cis*-7-methoxy-2-phenyl-2-phosphabicyclo[4.4.0]dec-8-en-5-one 2-oxide ((*rac*)-*cis*-**12**) ($CDCl_3$, 126 MHz).

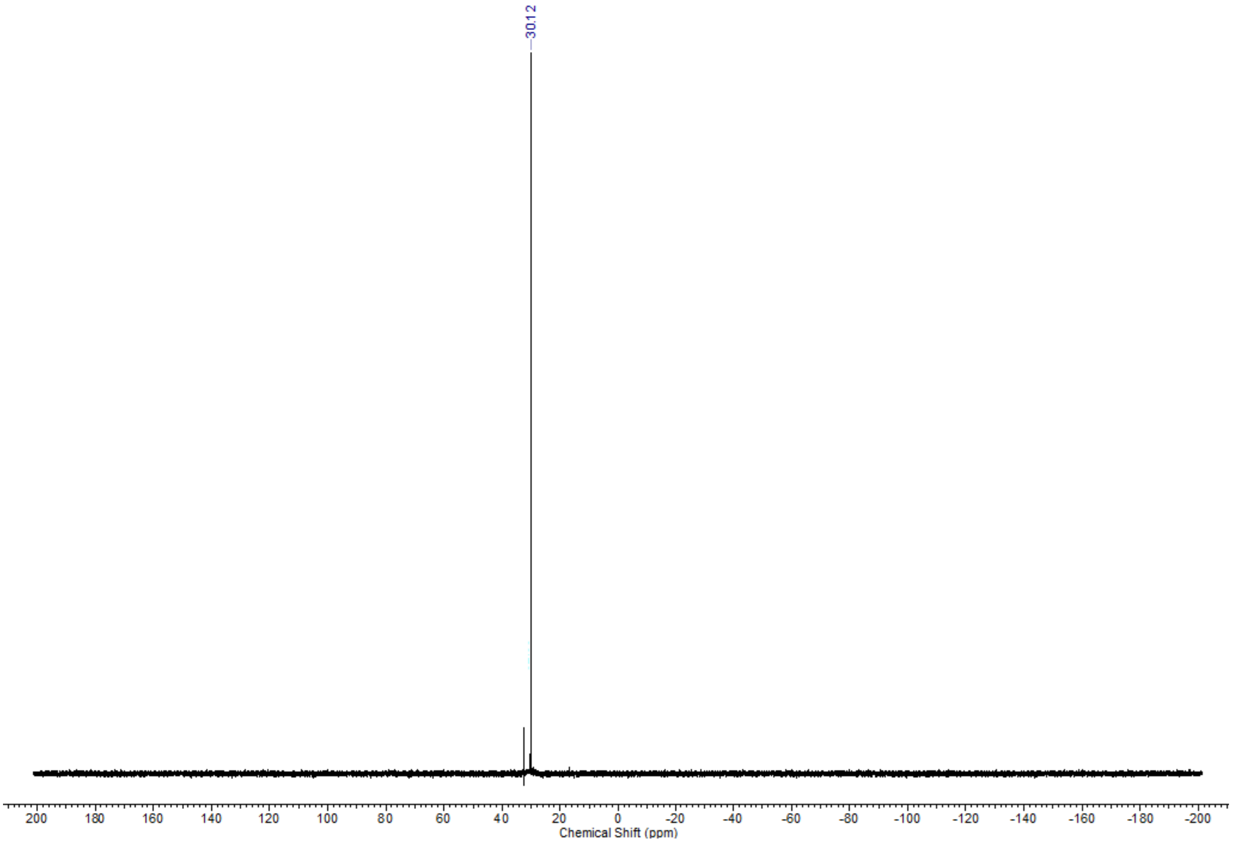

Fig. S21. ^{31}P NMR spectrum of (*rac*)-*cis*-7-methoxy-2-phenyl-2-phosphabicyclo[4.4.0]dec-8-en-5-one 2-oxide ((*rac*)-*cis*-**12**) (CDCl$_3$, 202 MHz).

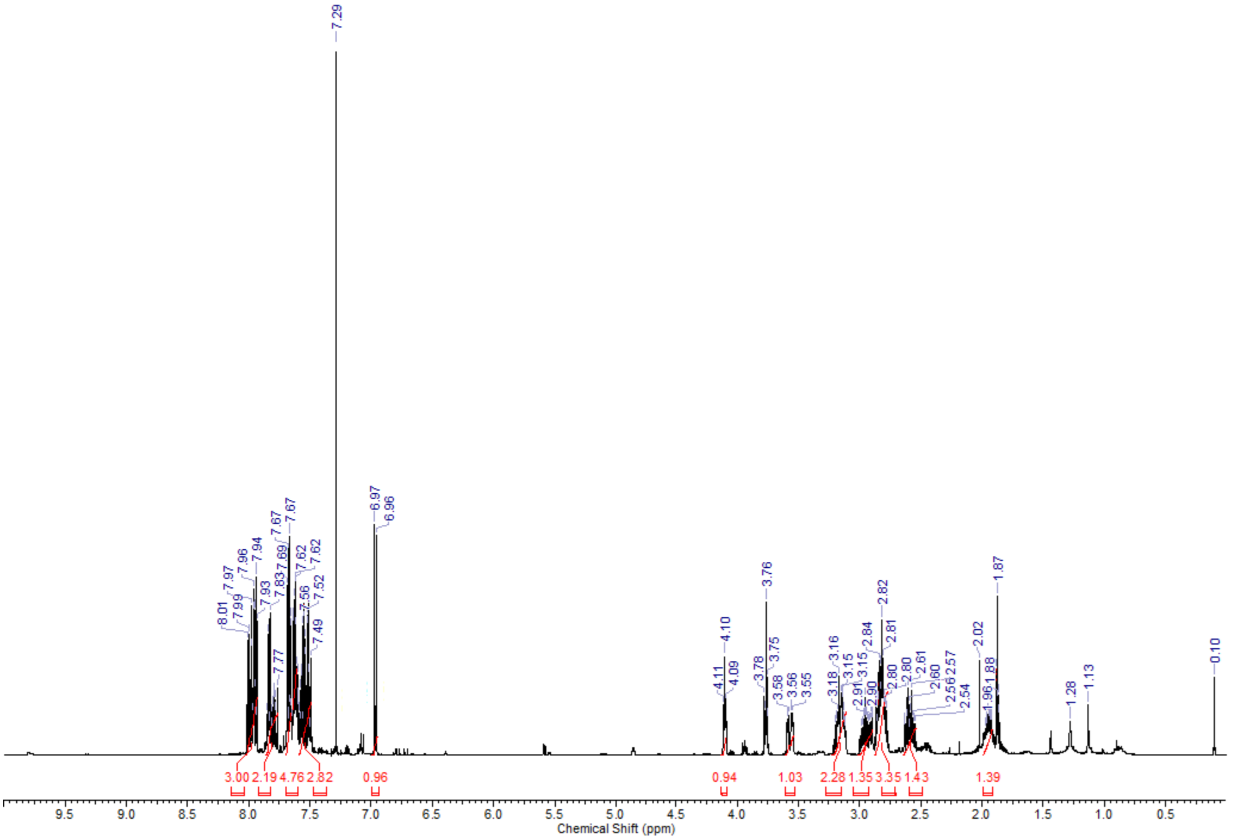

Fig. S22. ^{1}H NMR spectrum of (*rac*)-*cis*-1-phenyl-1-phospha-2,3,11,12,12a-hexahydrochrysen-4-one 1-oxide ((*rac*)-*cis*-**14**) (CDCl$_3$, 500 MHz).

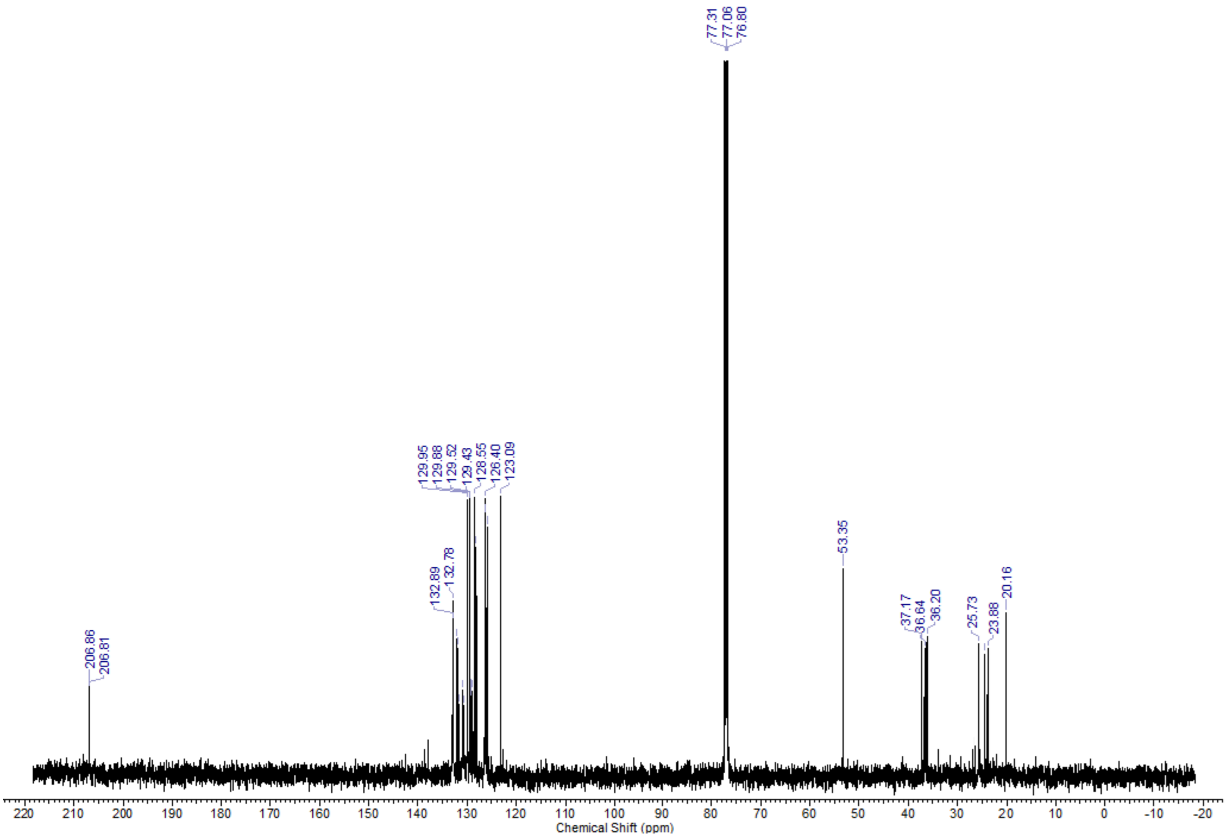

Fig. S23. ^{13}C NMR spectrum of (*rac*)-*cis*-1-phenyl-1-phospha-2,3,11,12,12a-hexahydrochrysen-4-one 1-oxide ((*rac*)-*cis*-**14**) ($CDCl_3$, 126 MHz).

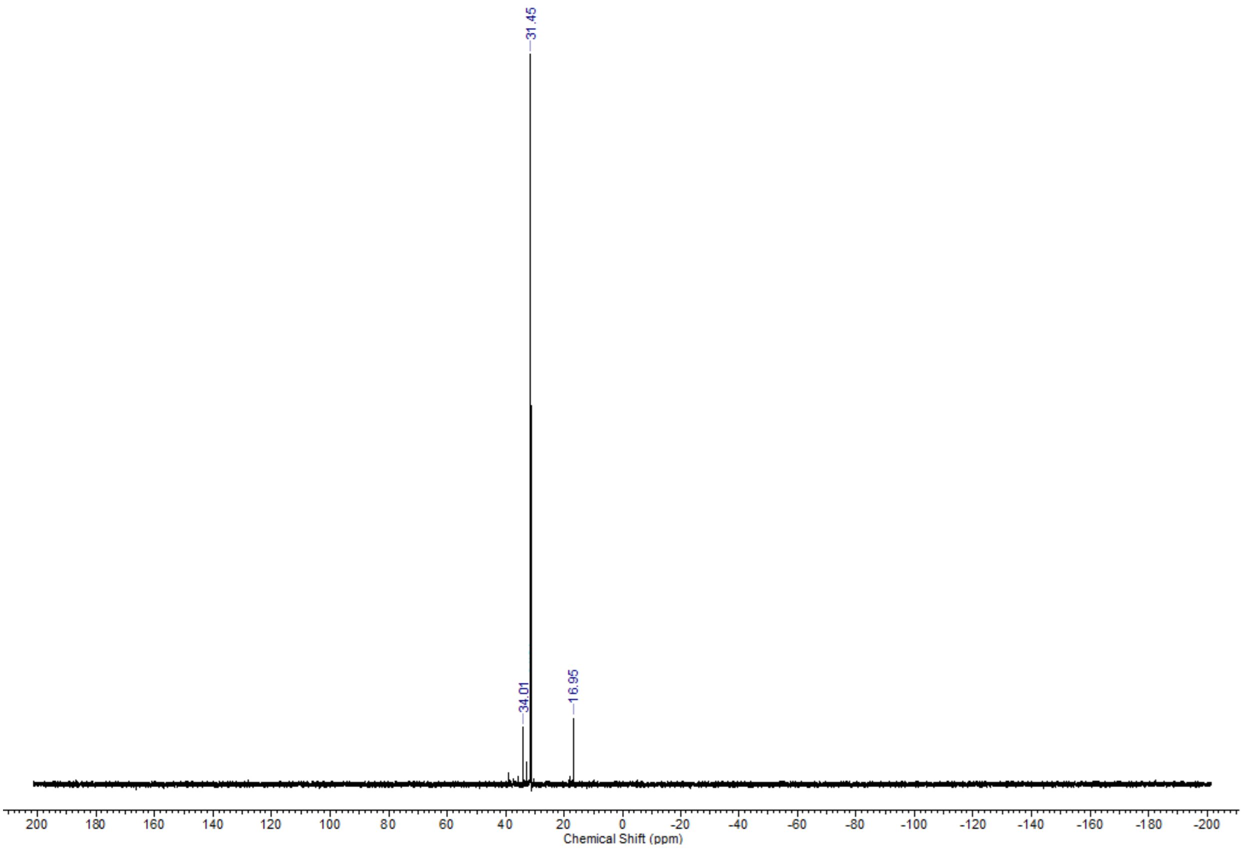

Fig. S24. ^{31}P NMR spectrum of (*rac*)-*cis*-1-phenyl-1-phospha-2,3,11,12,12a-hexahydrochrysen-4-one 1-oxide ((*rac*)-*cis*-**14**) ($CDCl_3$, 202 MHz).

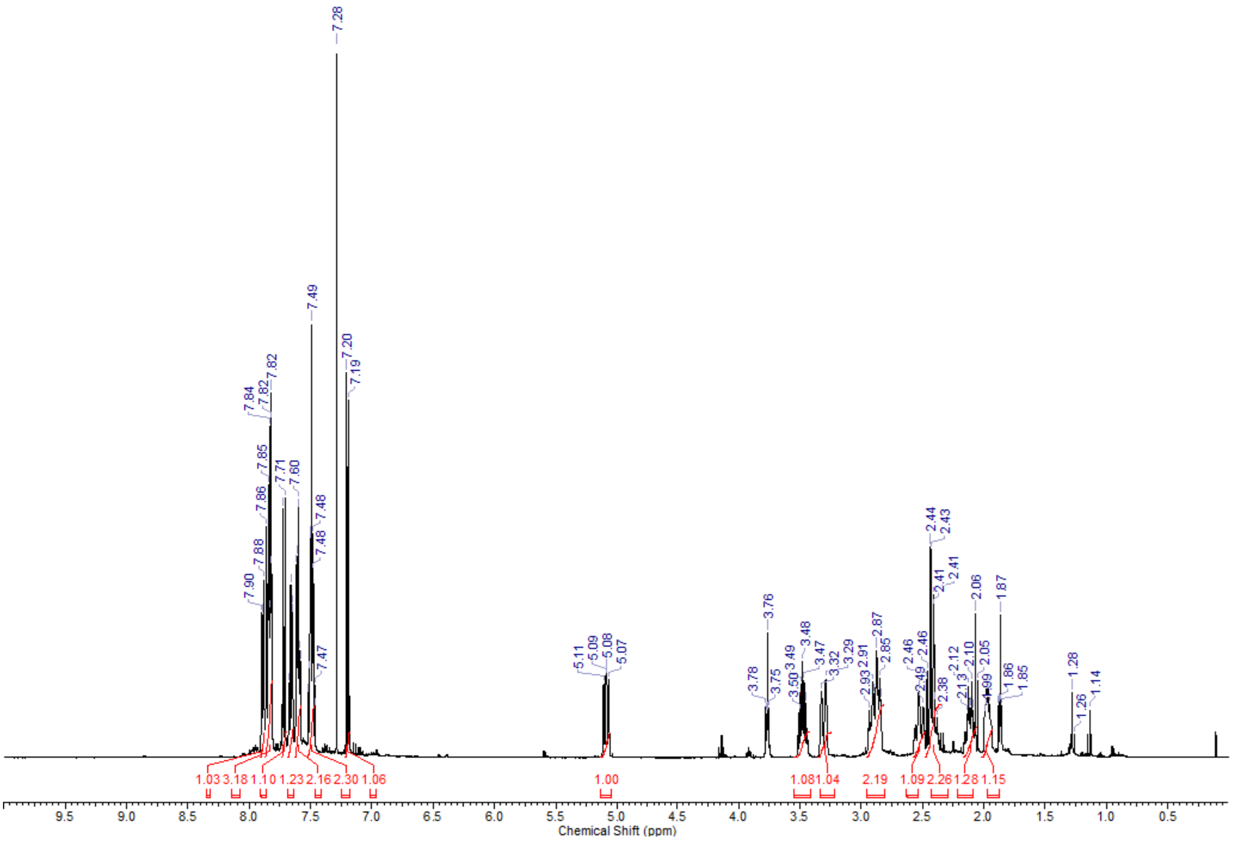

Fig. S25. ^{1}H NMR spectrum of (*rac*)-*trans*-1-phenyl-1-phospha-2,3,11,12,12a-hexahydrochrysen-4-one 1-oxide ((*rac*)-*trans*-**14**) ($CDCl_3$, 500 MHz).

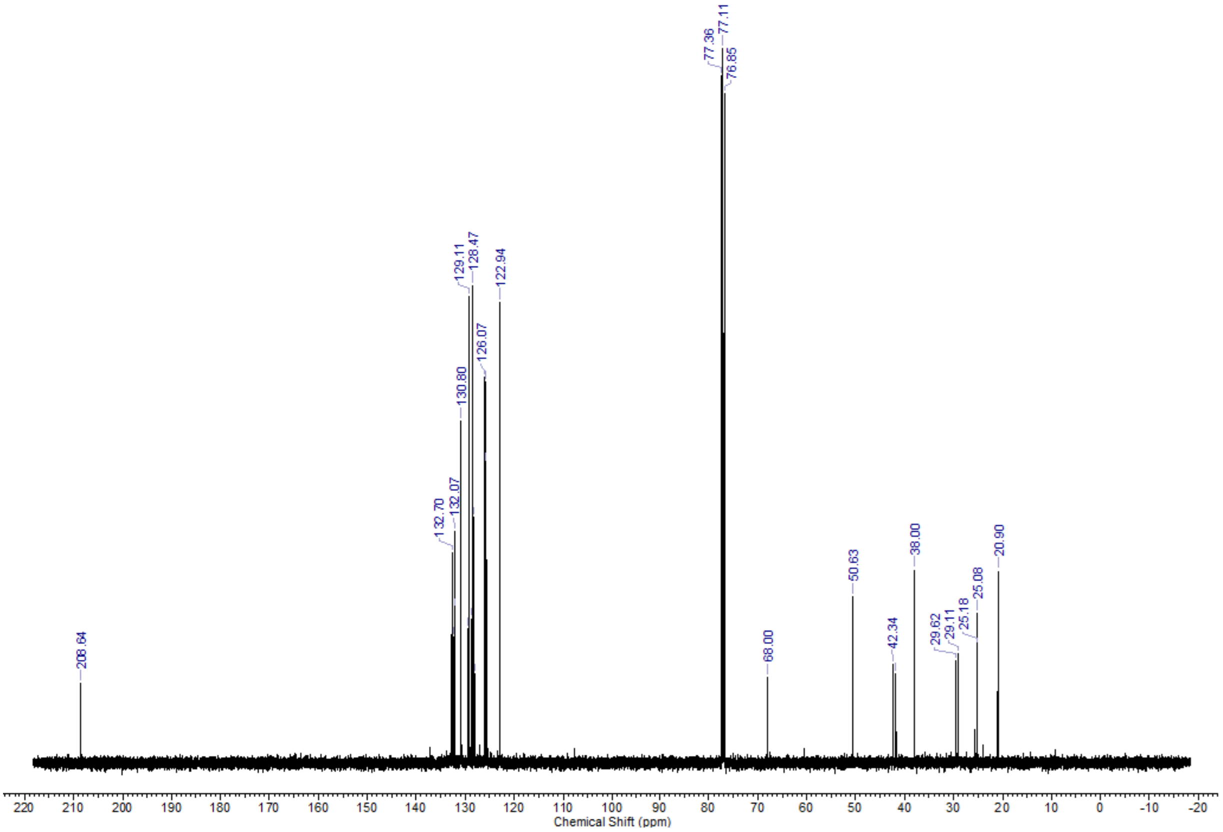

Fig. S26. ^{13}C NMR spectrum of (*rac*)-*trans*-1-phenyl-1-phospha-2,3,11,12,12a-hexahydrochrysen-4-one 1-oxide ((*rac*)-*trans*-**14**) ($CDCl_3$, 126 MHz).

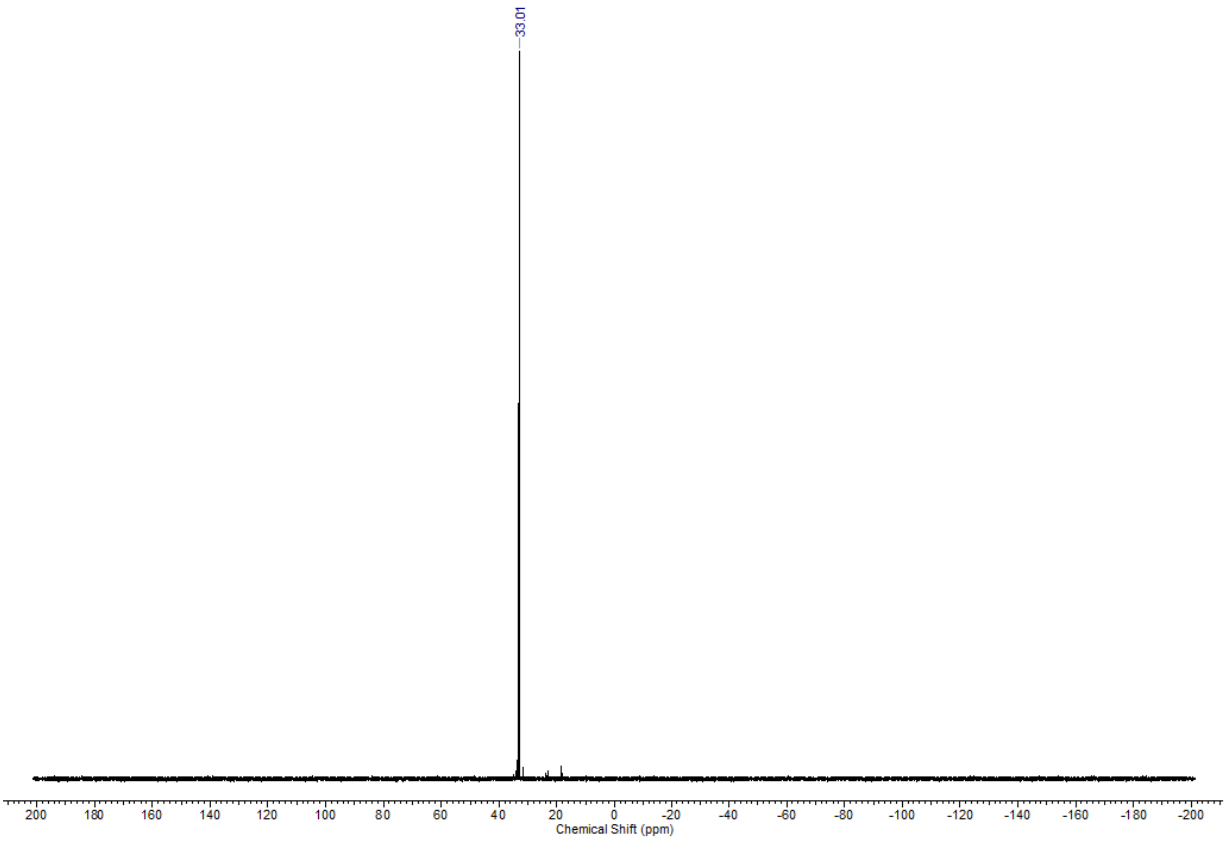

Fig. S27. ^{31}P NMR spectrum of (*rac*)-*trans*-1-phenyl-1-phospha-2,3,11,12,12a-hexahydrochrysen-4-one 1-oxide ((*rac*)-*trans*-**14**) ($CDCl_3$, 202 MHz).

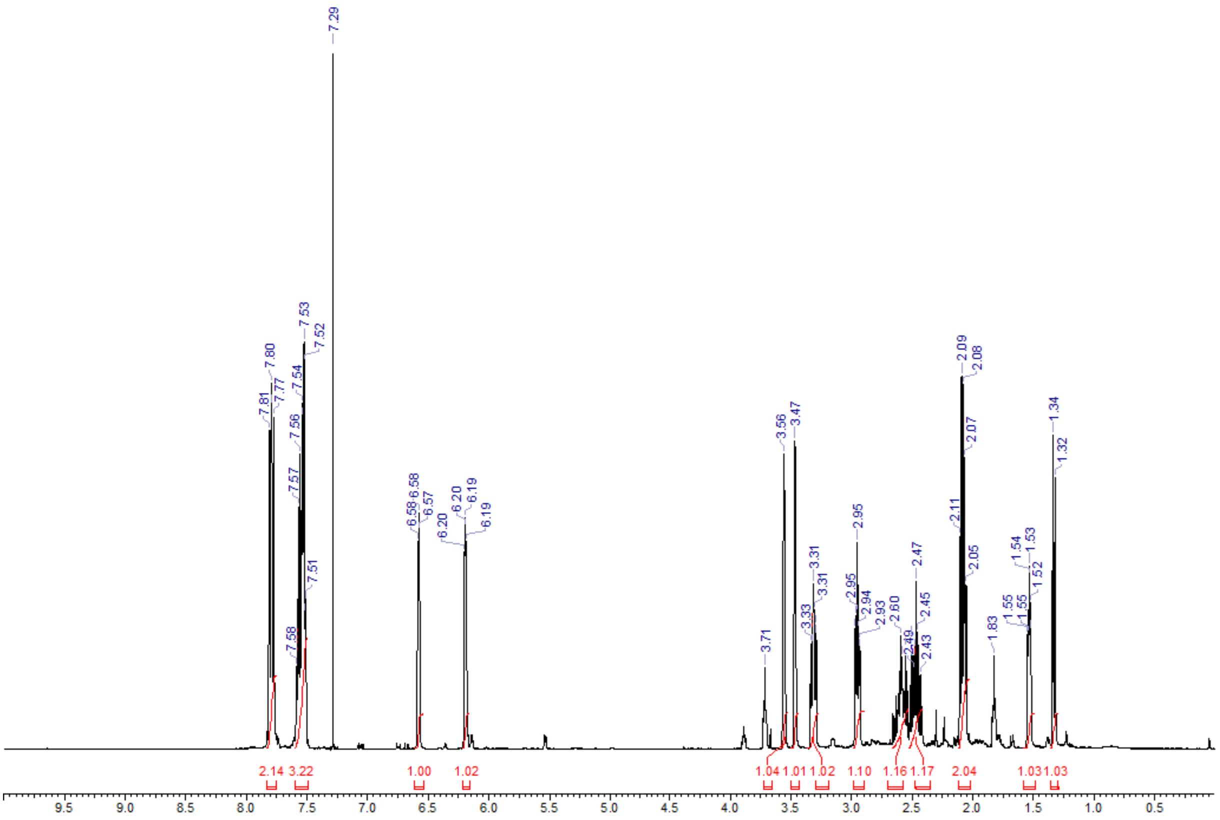

Fig. S28. ^{1}H NMR spectrum of (*rac*)-*endo*-3-phenyl-3-phosphatricyclo[6.2.1.0^{2,7}]undec-9-en-6-one 3-oxide ((*rac*)-*endo*-**17**) ($CDCl_3$, 500 MHz).

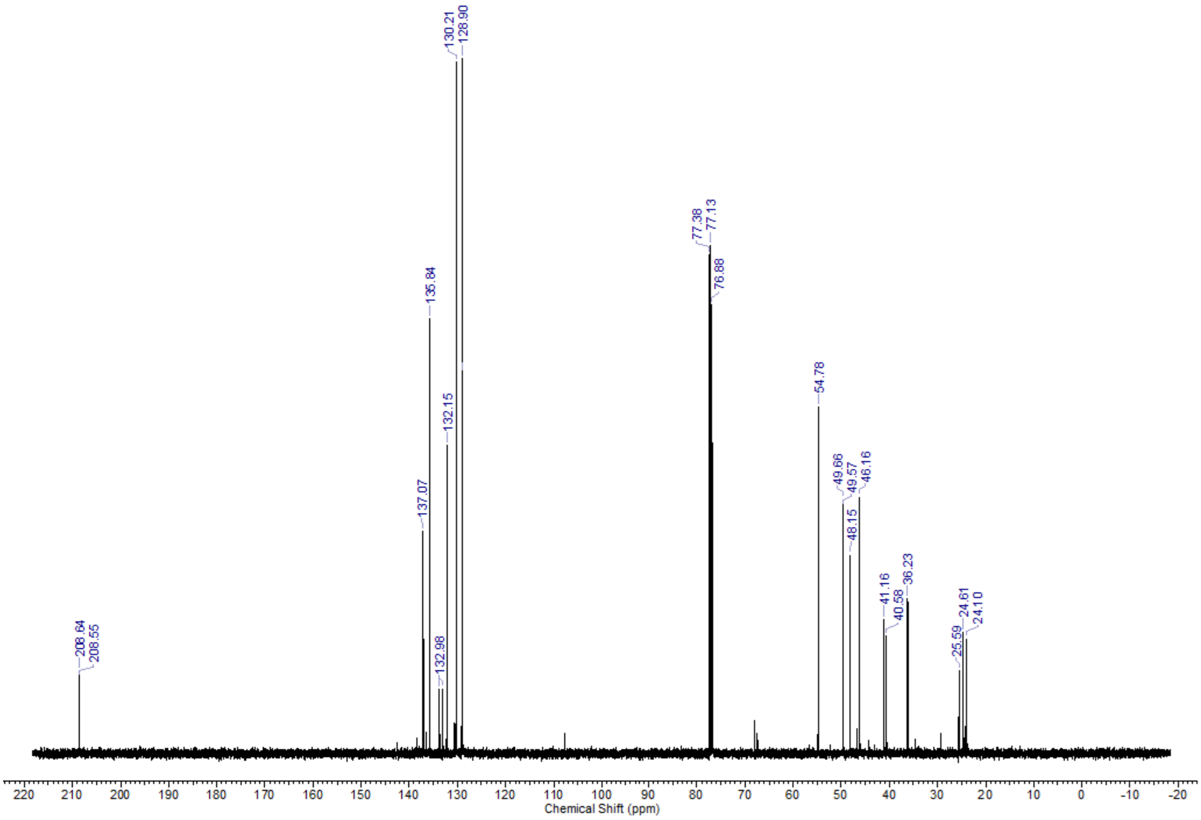

Fig. S29. ^{13}C NMR spectrum of (*rac*)-*endo*-3-phenyl-3-phosphatricyclo[6.2.1.0^{2,7}]undec-9-en-6-one 3-oxide ((*rac*)-*endo*-**17**) ($CDCl_3$, 126 MHz).

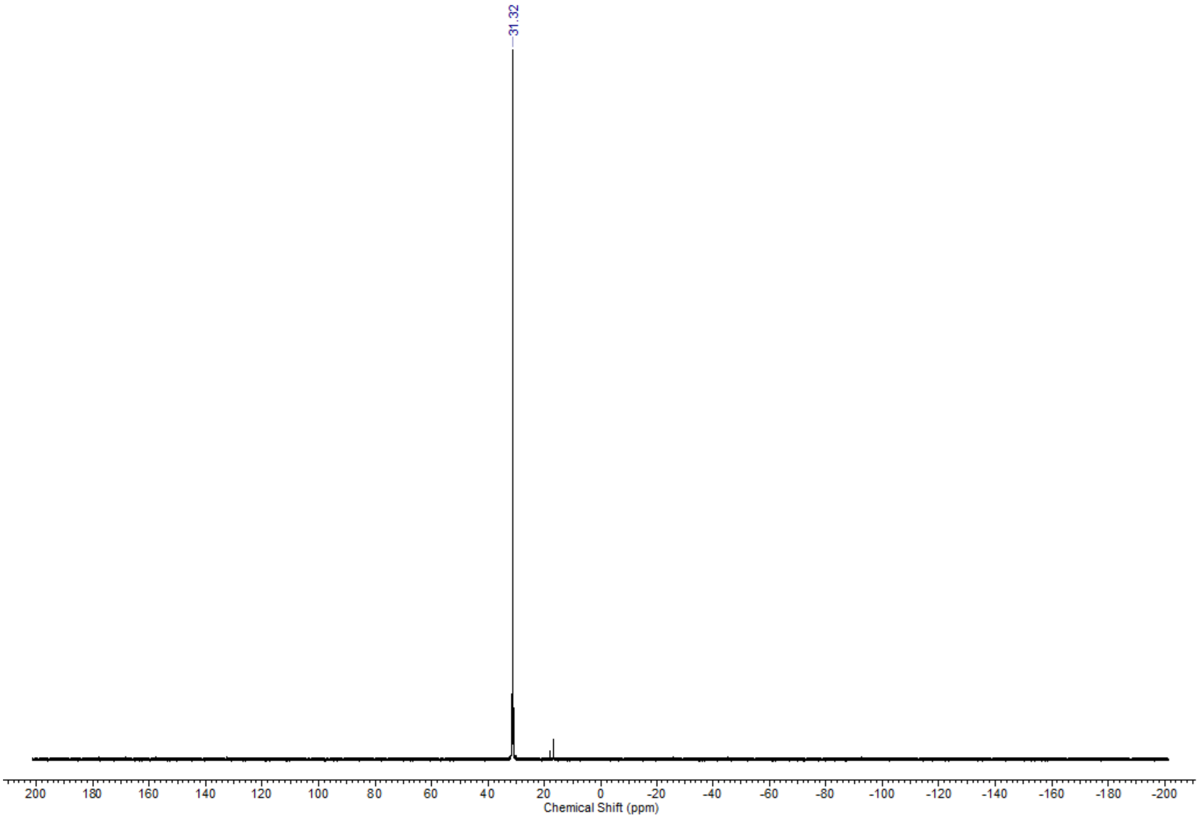

Fig. S30. ^{31}P NMR spectrum of (*rac*)-*endo*-3-phenyl-3-phosphatricyclo[6.2.1.0^{2,7}]undec-9-en-6-one 3-oxide ((*rac*)-*endo*-**17**) ($CDCl_3$, 202 MHz).

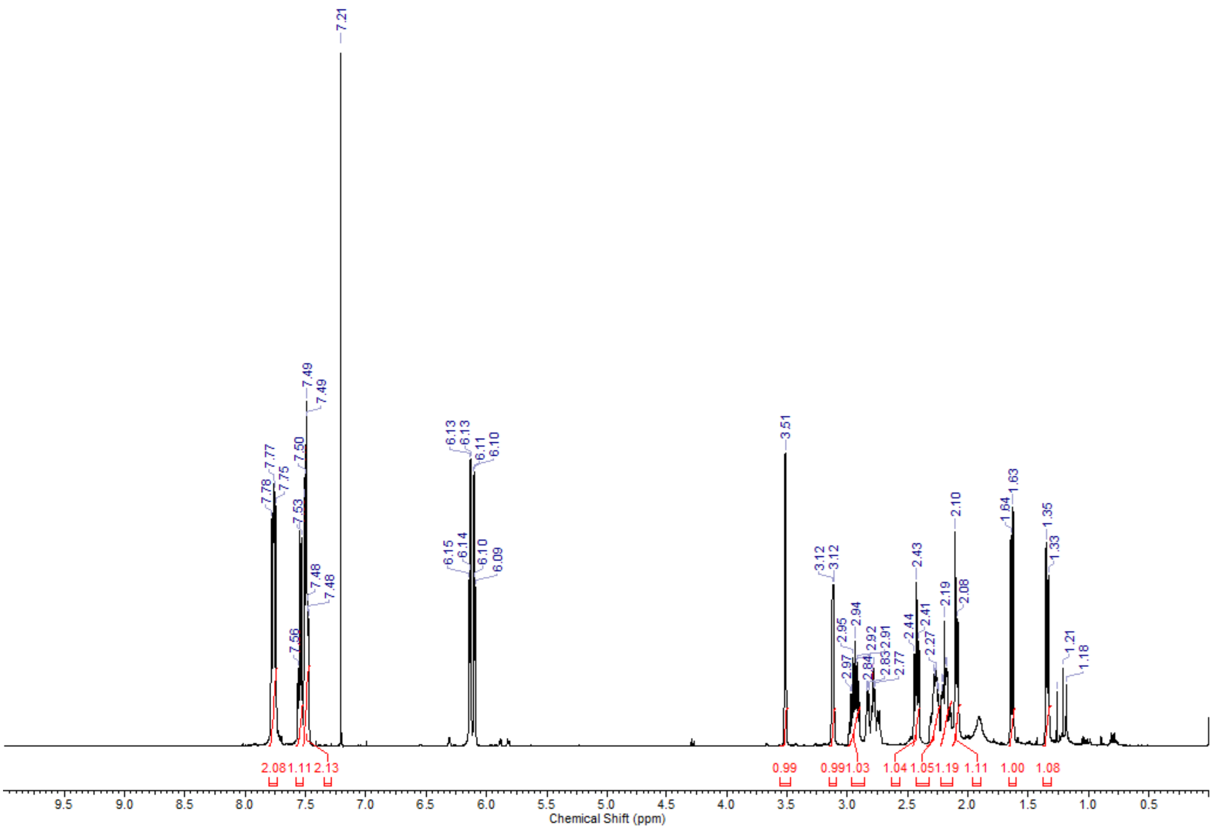

Fig. S31. ^{1}H NMR spectrum of (*rac*)-*exo*-3-phenyl-3-phosphatricyclo[6.2.1.0^{2,7}]undec-9-en-6-one 3-oxide ((*rac*)-*exo*-**17**) ($CDCl_3$, 500 MHz).

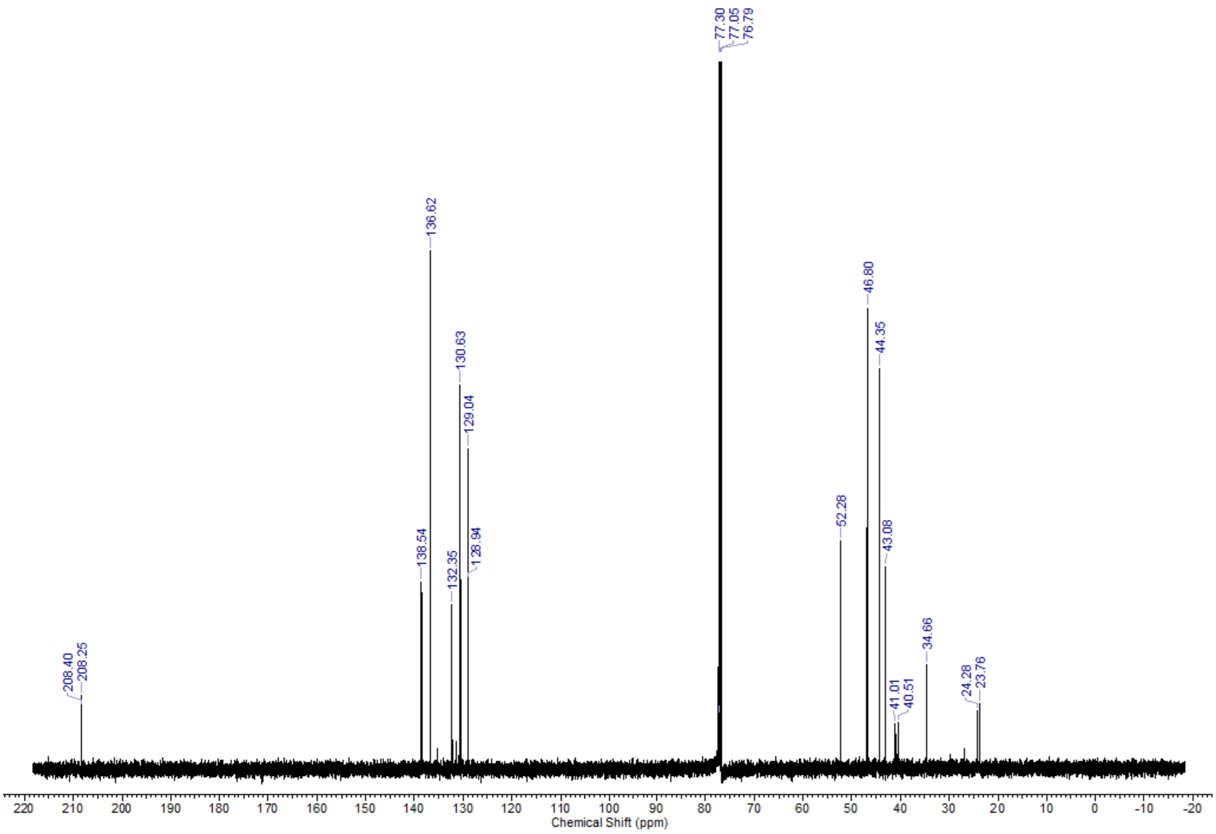

Fig. S32. ^{13}C NMR spectrum of (*rac*)-*exo*-3-phenyl-3-phosphatricyclo[6.2.1.0^{2,7}]undec-9-en-6-one 3-oxide ((*rac*)-*exo*-**17**) ($CDCl_3$, 126 MHz).

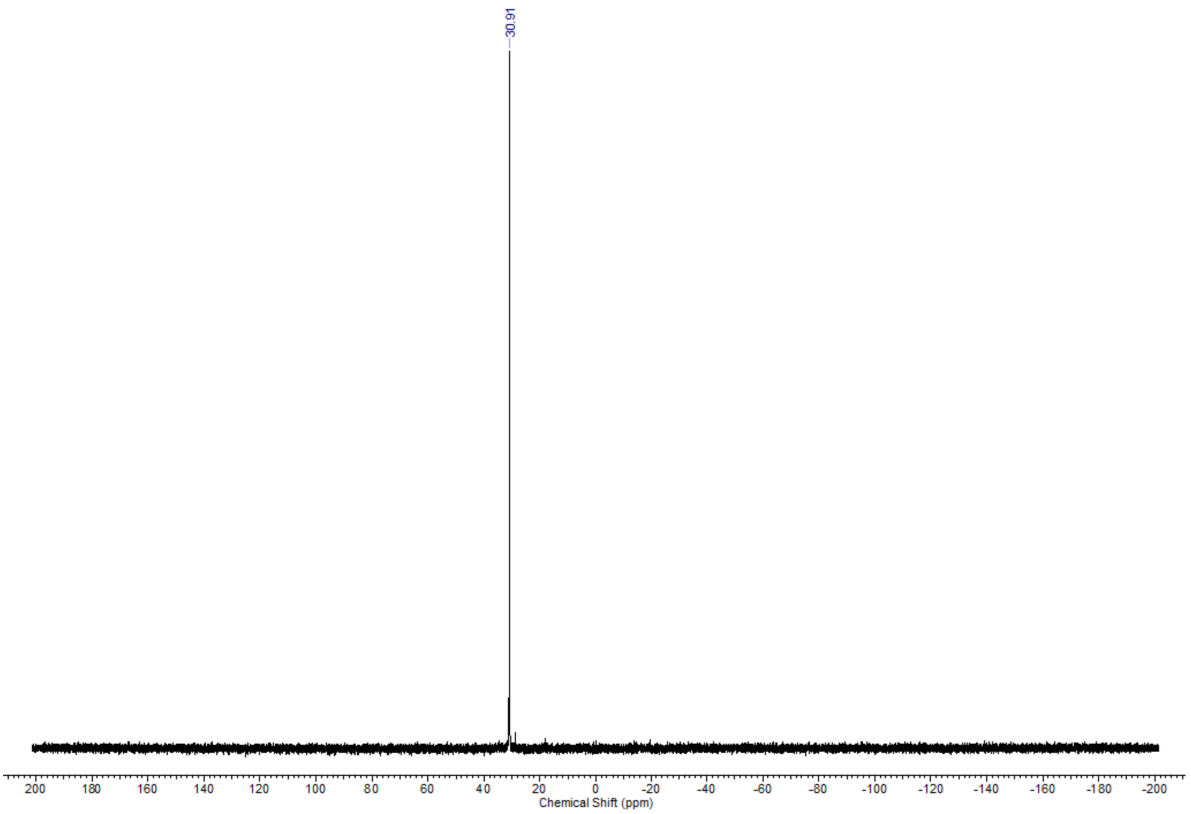

Fig. S33. ^{31}P NMR spectrum of (*rac*)-*exo*-3-phenyl-3-phosphatricyclo[6.2.1.0^{2,7}]undec-9-en-6-one 3-oxide ((*rac*)-*exo*-**17**) ($CDCl_3$, 202 MHz).

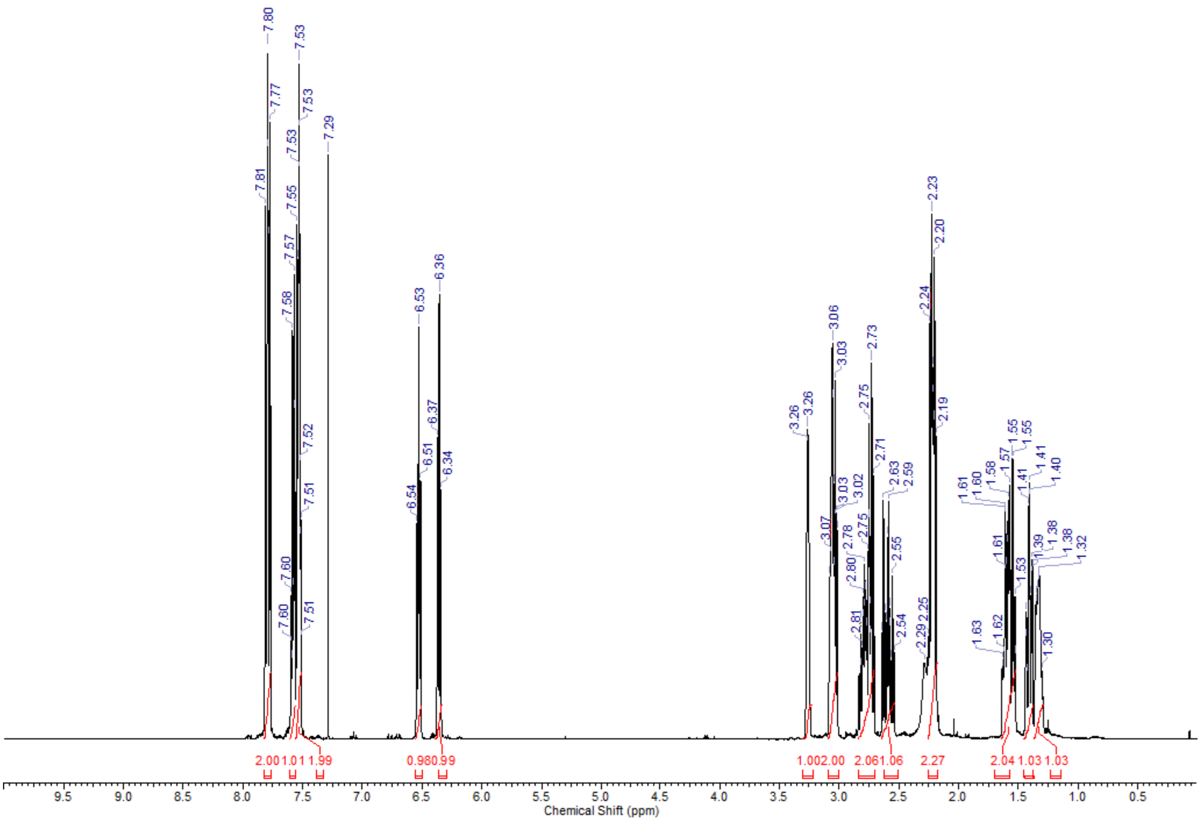

Fig. S34. ^{1}H NMR spectrum of (*rac*)-*endo*-3-phenyl-3-phosphatricyclo[6.2.2.0^{2,7}]dodeca-9-en-6-one 3-oxide ((*rac*)-*endo*-**18**) ($CDCl_3$, 500 MHz).

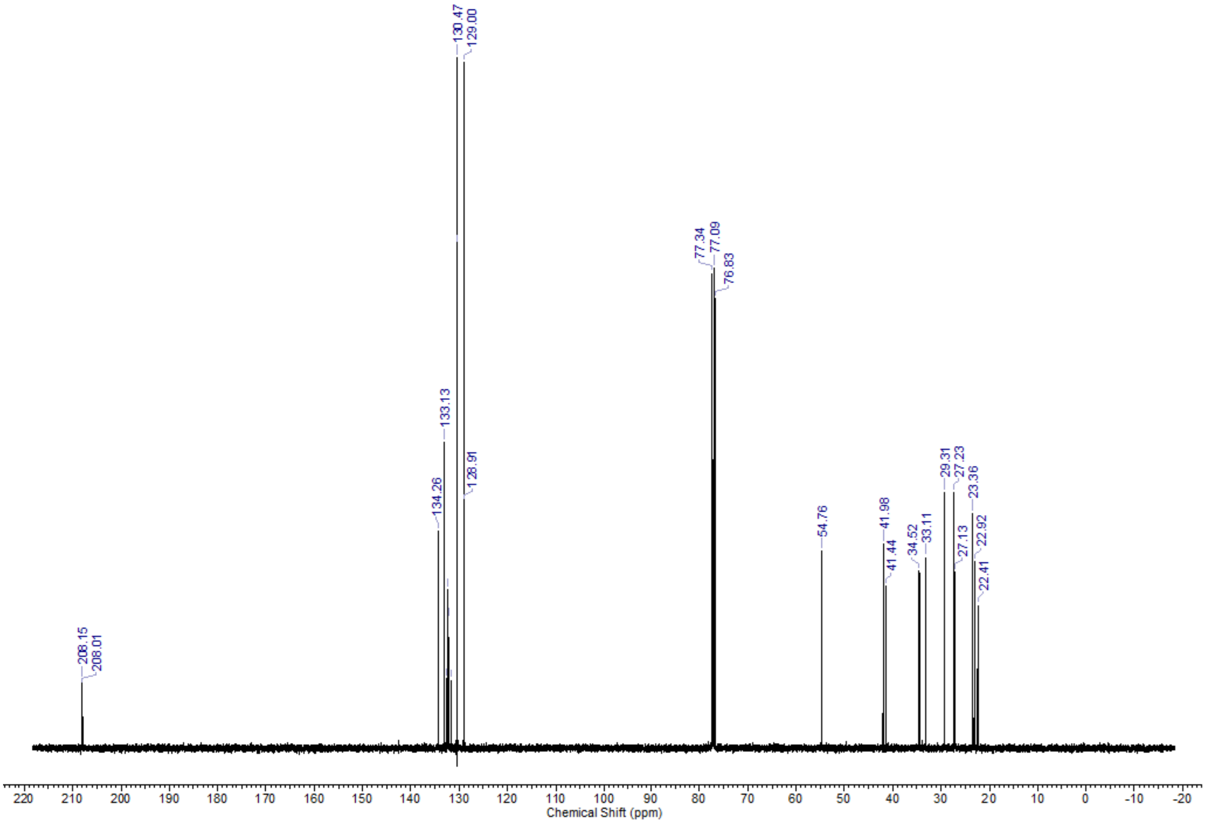

Fig. S35. ^{13}C NMR spectrum of (*rac*)-*endo*-3-phenyl-3-phosphatricyclo[6.2.2.0^{2,7}]dodeca-9-en-6-one 3-oxide ((*rac*)-*endo*-**18**) ($CDCl_3$, 126 MHz).

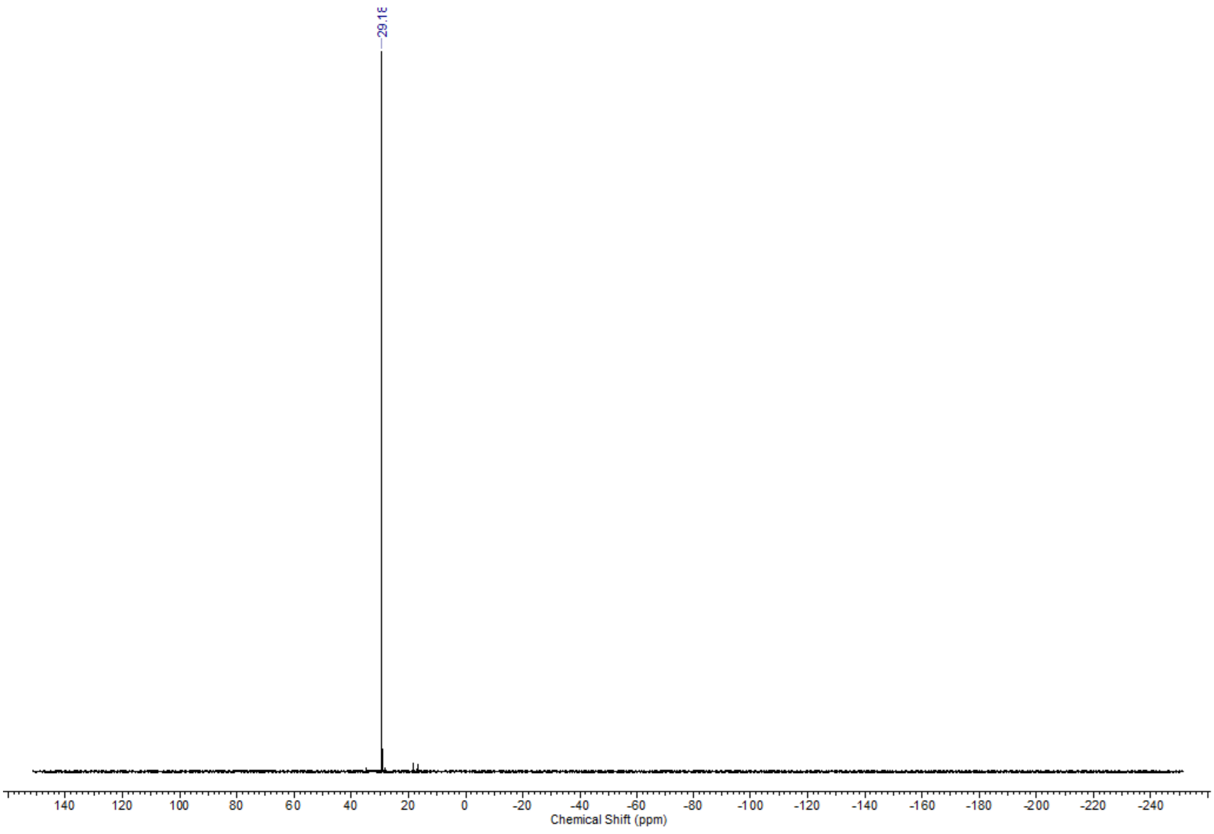

Fig. S36. ^{31}P NMR spectrum of (*rac*)-*endo*-3-phenyl-3-phosphatricyclo[6.2.2.0^{2,7}]dodeca-9-en-6-one 3-oxide ((*rac*)-*endo*-**18**) ($CDCl_3$, 202 MHz).

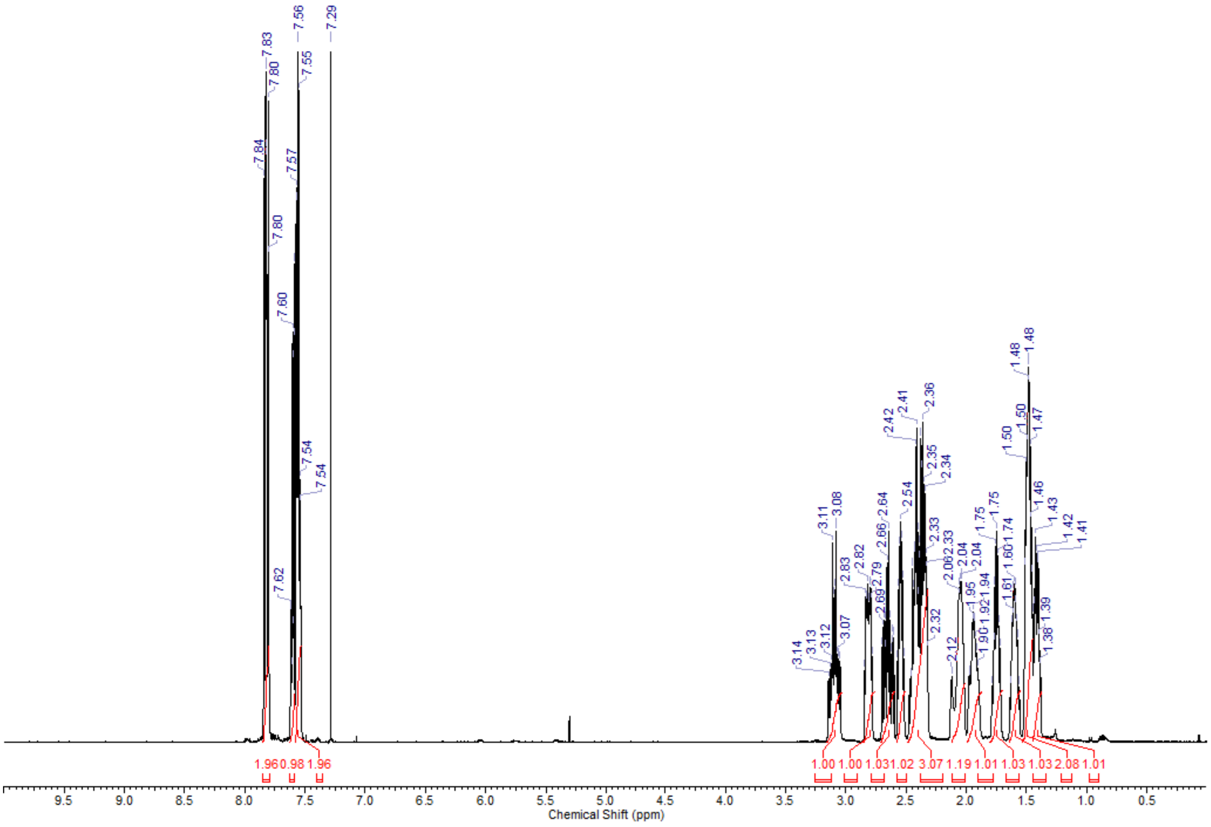

Fig. S37. ^{1}H NMR spectrum of (*R*P)-*cis*-2-phenyl-2-phosphabicyclo[4.4.0]dec-5-one 2-oxide ((***R*P)-*cis*-19**) ($CDCl_3$, 500 MHz).

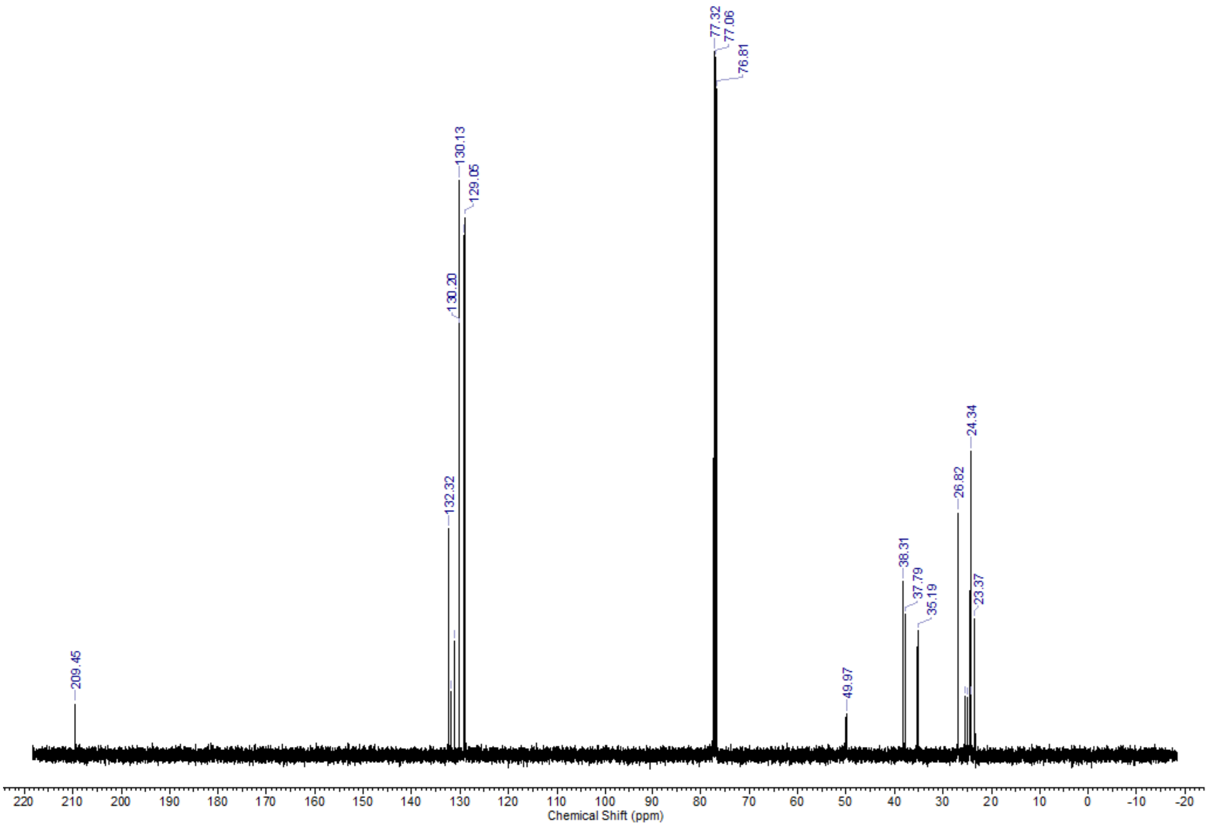

Fig. S38. ^{13}C NMR spectrum of (*R*P)-*cis*-2-phenyl-2-phosphabicyclo[4.4.0]dec-5-one 2-oxide ((*R*P)-*cis*-**19**) ($CDCl_3$, 126 MHz).

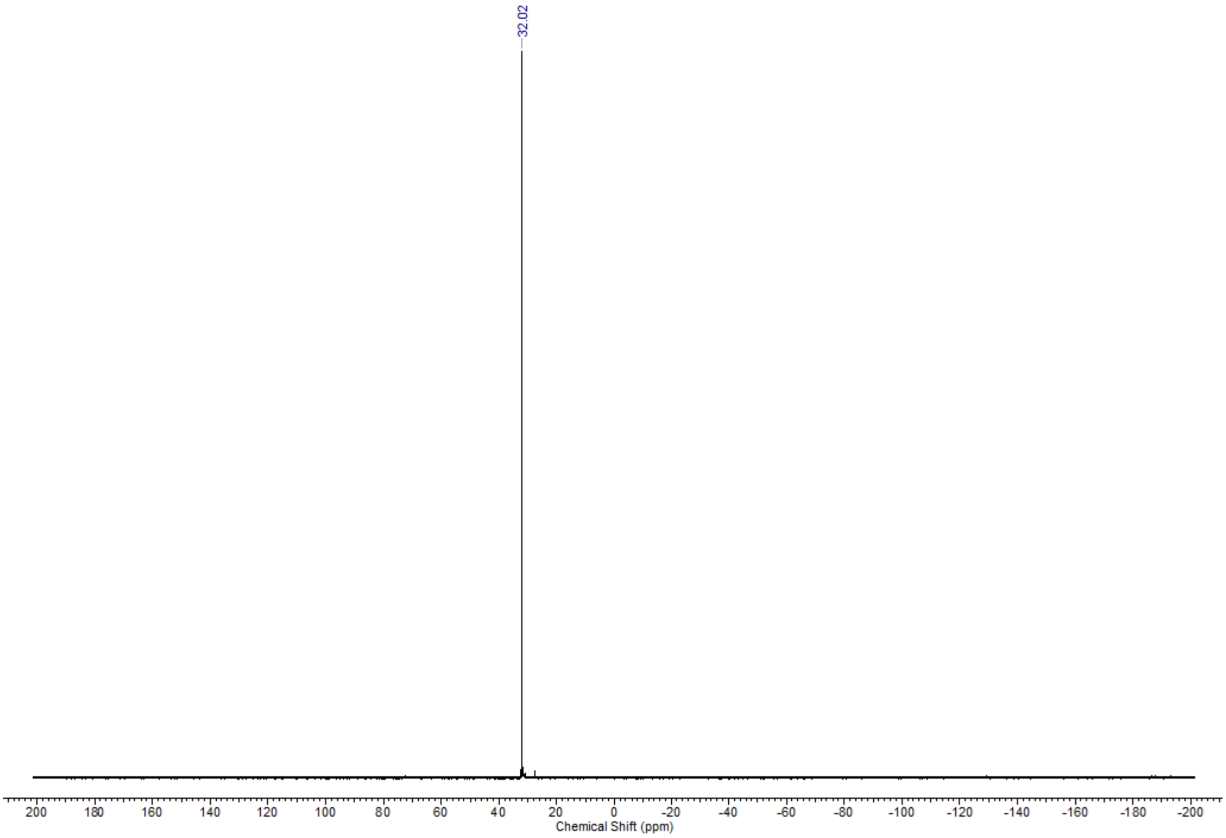

Fig. S39. ^{31}P NMR spectrum of (R_P)-*cis*-2-phenyl-2-phosphabicyclo[4.4.0]dec-5-one 2-oxide ((R_P)-*cis*-**19**) ($CDCl_3$, 202 MHz).

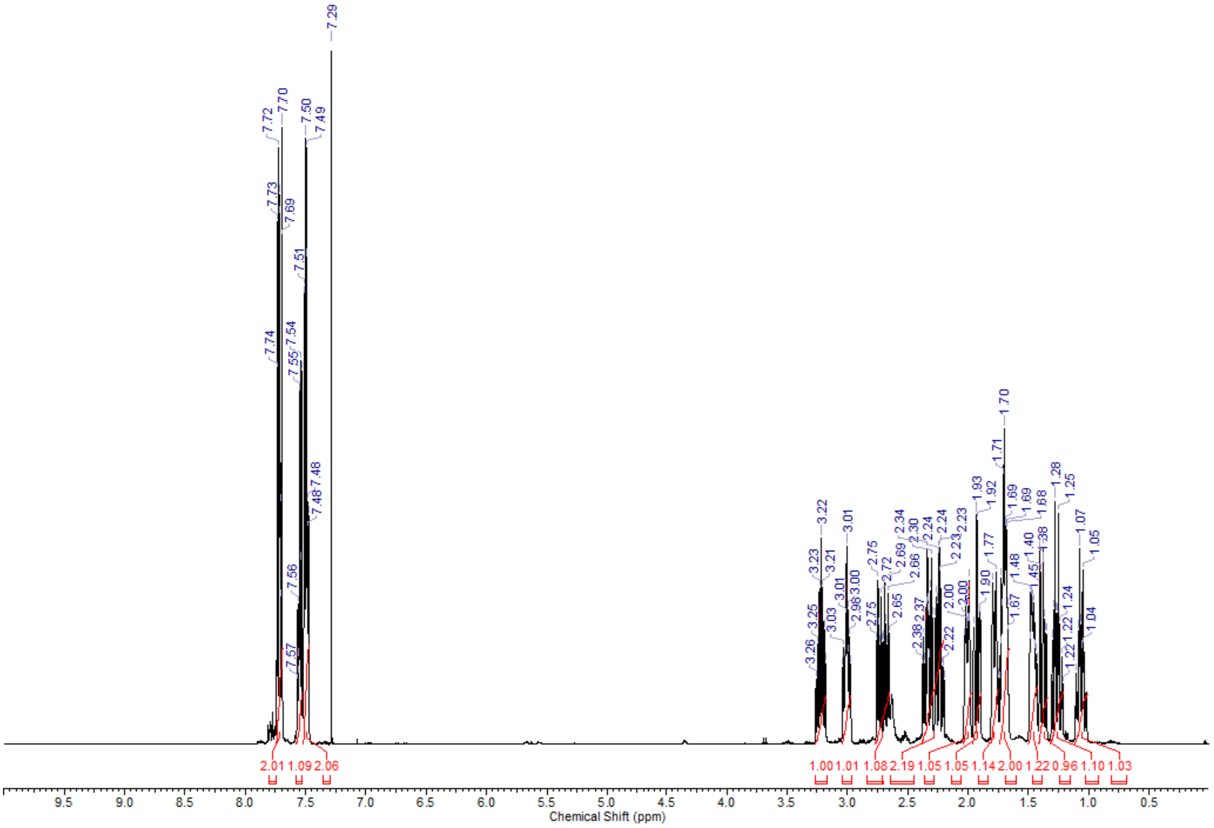

Fig. S40. ^{1}H NMR spectrum of (R_P)-*trans*-2-phenyl-2-phosphabicyclo[4.4.0]dec-5-one 2-oxide ((R_P)-*trans*-**19**) ($CDCl_3$, 500 MHz).

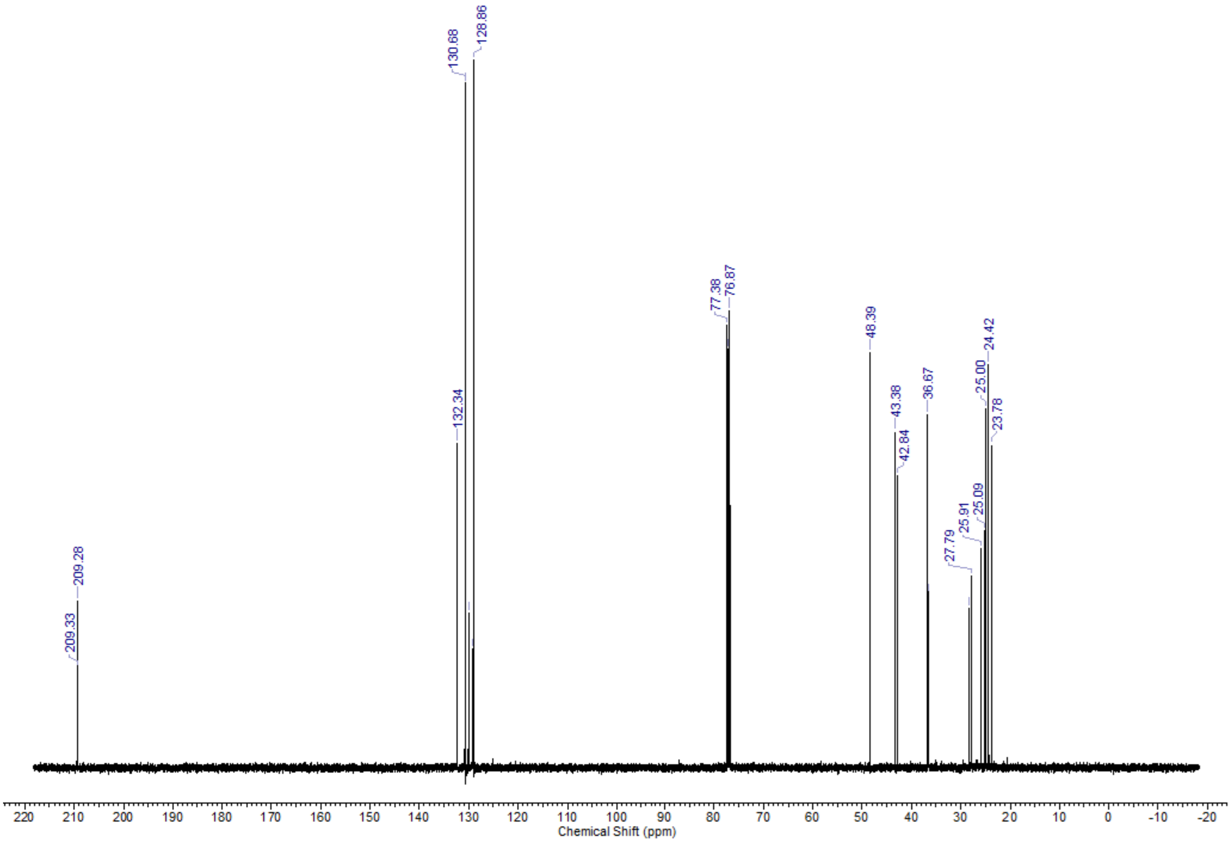

Fig. S41. ^{13}C NMR spectrum of (R_P)-*trans*-2-phenyl-2-phosphabicyclo[4.4.0]dec-5-one 2-oxide ((R_P)-*trans*-**19**) ($CDCl_3$, 126 MHz).

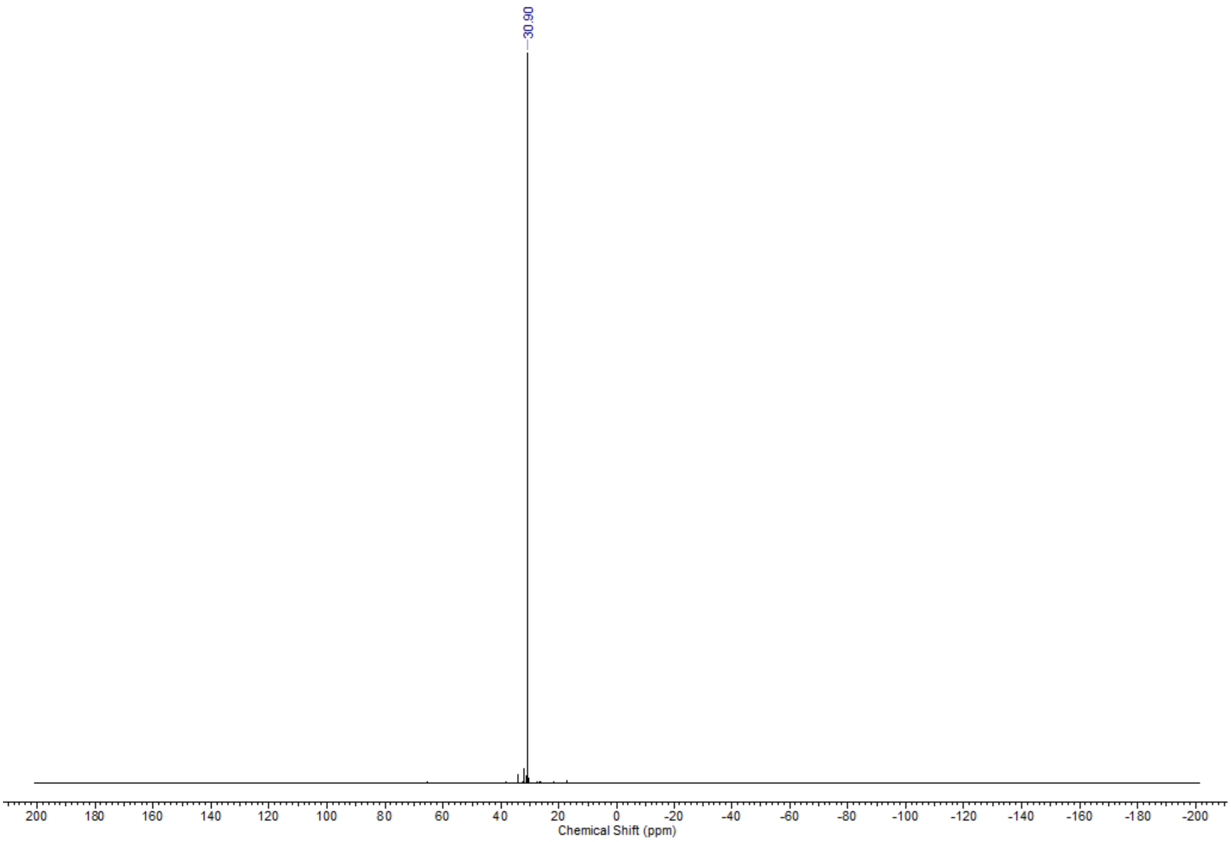

Fig. S42. ^{31}P NMR spectrum of (R_P)-*trans*-2-phenyl-2-phosphabicyclo[4.4.0]dec-5-one 2-oxide ((R_P)-*trans*-**19**) ($CDCl_3$, 202 MHz).

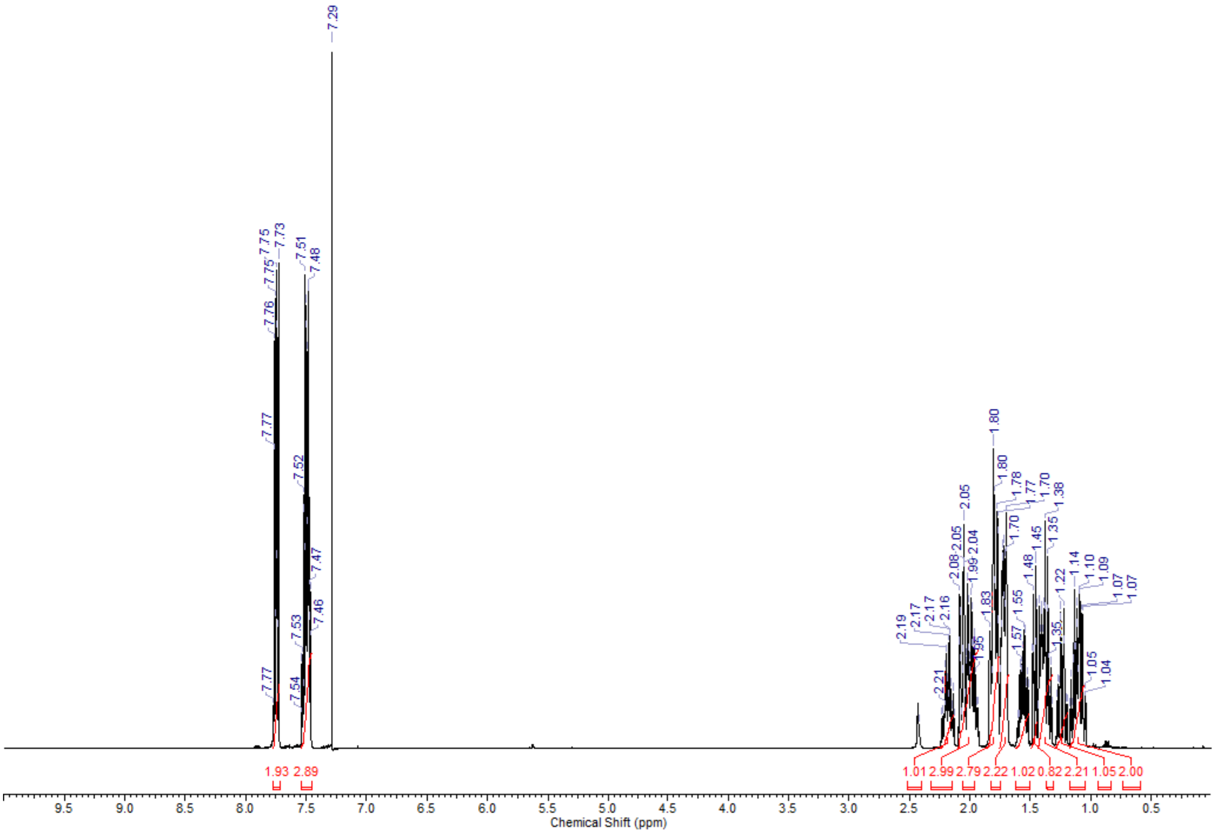

Fig. S43. ^{1}H NMR spectrum of (R_{P})-*trans*-2-phenyl-2-phosphabicyclo[4.4.0]decane 2-oxide ((R_{P})-*trans*-**20**) ($CDCl_3$, 500 MHz).

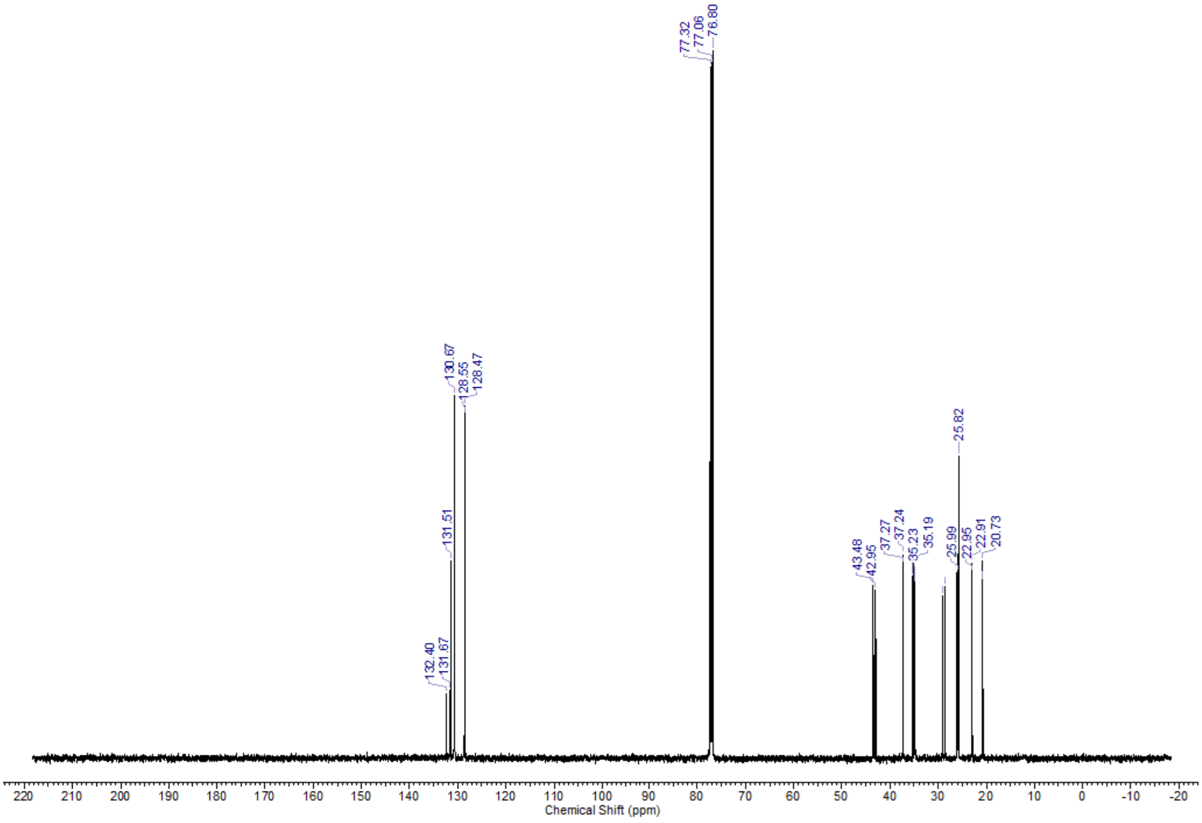

Fig. S44. ^{13}C NMR spectrum of (R_{P})-*trans*-2-phenyl-2-phosphabicyclo[4.4.0]decane 2-oxide ((R_{P})-*trans*-**20**) ($CDCl_3$, 126 MHz).

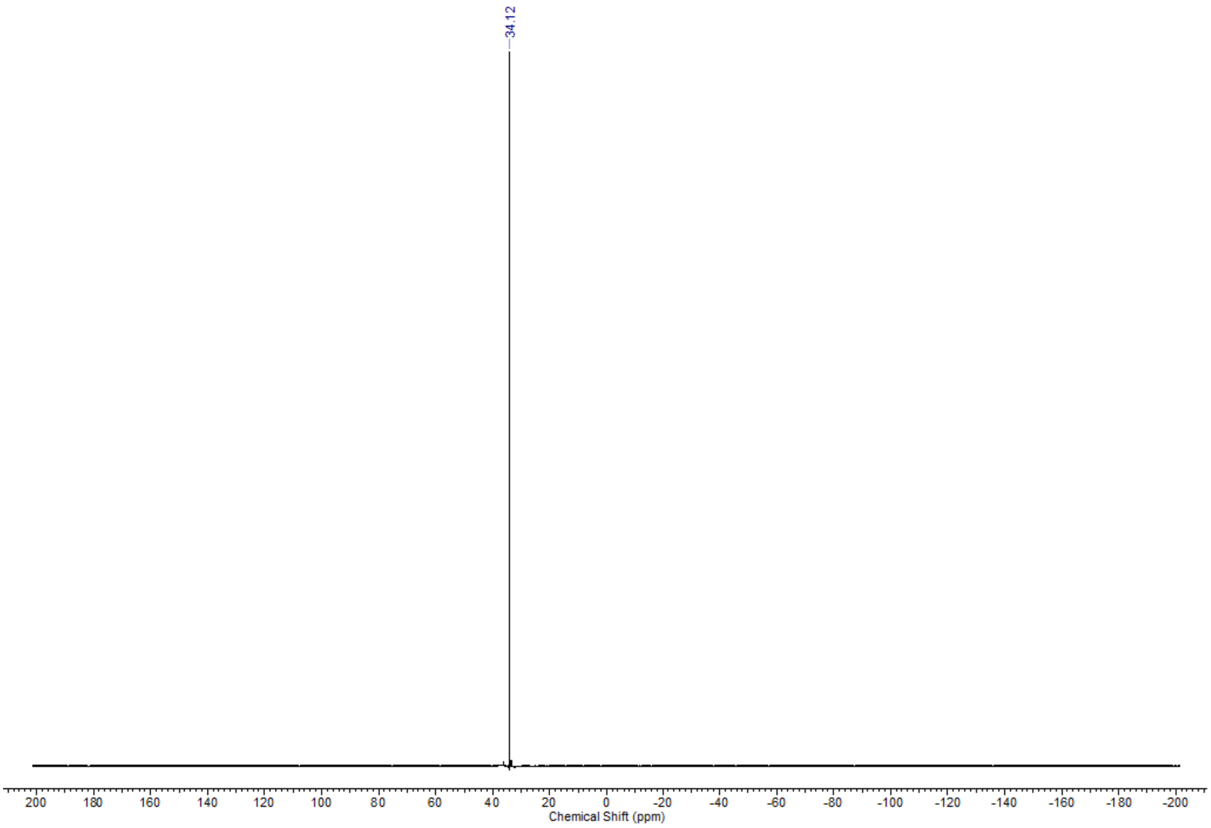

Fig. S45. ^{31}P NMR spectrum of (R_P)-*trans*-2-phenyl-2-phosphabicyclo[4.4.0]decane 2-oxide ((R_P)-*trans*-**20**) ($CDCl_3$, 202 MHz).
